# Mechanistic ODE modelling of ovarian tumour microenvironment dynamics under hesperidin exposure

**DOI:** 10.1098/rsos.260368

**Published:** 2026-10-28

**Authors:** Radosław Dzik

**Affiliations:** Department of Clinical Engineering, Academy of Silesia, Katowice, Poland

**Keywords:** ovarian cancer, PA-1, hesperidin, ODE, mechanistic model

## Abstract

Ovarian cancer progression is driven by complex interactions among tumour, immune, stromal, metabolic and angiogenic processes. Although hesperidin exhibits anti-cancer and immunomodulatory activities, its integrated effects on the tumour microenvironment (TME) remain insufficiently understood. This study presents a mechanistic ODE-based framework integrating tumour growth, immune regulation, macrophage polarization, cancer-associated fibroblast (CAF)-mediated extracellular matrix (ECM) remodelling, hypoxia-inducible factor (HIF)−1α/ vascular endothelial growth factor (VEGF)-dependent angiogenesis, glucose–lactate metabolism and redox signalling within a hypothetical ovarian TME centred on PA-1 cells. Simulations across multiple hesperidin concentrations predicted dose-dependent suppression of tumour growth, reduced HIF−1α accumulation, angiogenesis, ECM stiffening and lactate production, accompanied by enhanced anti-tumour immune activity. Local one-at-a-time (OAT) and global Sobol’ sensitivity analyses identified tumour growth kinetics, CAF–ECM interactions, metabolic regulation and selected hesperidin-response parameters as principal determinants of system behaviour. Although not calibrated for quantitative prediction, the proposed framework provides mechanistic insight into how hesperidin-mediated perturbations propagate through interconnected tumour–immune–stromal networks. The model should be regarded as an exploratory, hypothesis-generating platform supporting future experimental validation of hesperidin-mediated mechanisms in ovarian cancer.

## 1. Introduction

Natural compounds remain an important source of therapeutic agents, with many plant-derived molecules undergoing chemical or biotechnological refinement for use in endocrine, cardiovascular and oncological treatment. Polyphenols and flavonoids exert well-documented antioxidant, anti-inflammatory and anti-proliferative effects, modulating key signalling pathways such as nuclear factor (NF)−κB, PI3K/AKT, transforming growth factor (TGF)−β and p53 [1–5]. Compounds, including caffeic acid, its phenethyl ester caffeic acid phenethyl ester (CAPE), apigenin, chrysin, kaempferol, luteolin, genistein and hesperidin, have therefore attracted attention as candidates for anti-cancer intervention. Hesperidin, a natural citrus flavonoid, has shown chemopreventive and chemosensitizing properties, regulating inflammation, proliferation and angiogenesis and improving cancer-cell susceptibility to therapy. Its reported effects on immune cells, extracellular matrix (ECM) regulation and angiogenic signalling suggest a broader role as a tumour microenvironment (TME) modulator [6], with possible relevance for ovarian cancer. Ovarian cancer presents a major global burden: GLOBOCAN 2022 predicts a greater than 55% increase in cases and a 70% rise in annual deaths by 2050 [7]. Despite advances in diagnostics and treatment, 5-year survival remains below 45% in most regions, largely because the disease is detected at advanced stages and lacks effective screening [8]. Genomic instability, microenvironmental alterations and chemoresistance make ovarian cancer particularly suitable for computational analysis of tumour–immune–stromal interactions. The PA-1 ovarian teratocarcinoma line is widely used to study natural-compound effects on molecular pathways, protein expression and cell–microenvironment interactions. As a pluripotent germ-cell-derived tumour representing 2–3% of ovarian malignancies and demonstrating high aggressiveness [9], PA-1 offers a relevant system for mechanistic modelling.

Beyond tumour-intrinsic genetic alterations, ovarian cancer progression is strongly influenced by the TME, which comprises immune cells, cancer-associated fibroblasts (CAFs), ECM components, vascular networks and diverse soluble signalling mediators. Extensive evidence indicates that reciprocal interactions between tumour and stromal compartments contribute to immune evasion, angiogenesis, metabolic reprogramming, ECM remodelling and therapeutic resistance. In particular, immunosuppressive macrophages, regulatory T cells, CAFs and hypoxia-associated signalling pathways have emerged as important regulators of disease progression and treatment outcome in ovarian cancer. Consequently, understanding the dynamic interactions among these components has become a major objective of contemporary ovarian cancer research [10–12].

Hesperidin has attracted increasing interest in oncology because its biological activity extends beyond direct cytotoxicity. Experimental studies have demonstrated that hesperidin and its aglycone hesperetin can influence multiple hallmarks of cancer, including cell proliferation, apoptosis, oxidative-stress regulation, angiogenesis, invasion and metastatic potential. Mechanistically, hesperidin has been reported to modulate signalling pathways involving Phosphoinositide 3-kinase/protein kinase B PI3K/AKT, mitogen-activated protein kinase/extracellular signal-regulated kinase (MAPK/ERK), NF kappa-light-chain-enhancer of activated B cells NF−κB, signal transducer and activator of transcription 3 (STAT3), vascular endothelial growth factor (VEGF) and hypoxia-inducible factor (HIF)−1α, while also affecting redox homoeostasis through regulation of reactive oxygen species (ROS)-dependent pathways [4,6,13]. In addition, emerging evidence suggests that hesperidin may influence immune-cell activity, macrophage polarization, ECM remodelling and tumour metabolism, indicating that its biological effects are not restricted to cancer cells alone but may extend to multiple components of the TME [6,13,14]. These pleiotropic properties make hesperidin an attractive candidate for systems-level investigation using mechanistic modelling approaches.

Recent advances in computational oncology have highlighted the potential of mechanistic mathematical models to integrate biological knowledge across multiple scales of tumour organization. Unlike purely data-driven approaches, mechanistic frameworks explicitly represent biological processes and interactions, enabling the exploration of causal relationships, hypothesis generation and evaluation of potential therapeutic interventions under controlled *in silico* conditions. Such approaches are increasingly recognized as valuable tools for investigating tumour growth, treatment response and microenvironmental regulation, particularly in settings where comprehensive experimental measurements remain difficult to obtain [15–20].

Despite growing evidence describing the anti-cancer activity of hesperidin, its integrated effects across multiple interacting components of the ovarian TME remain insufficiently understood, particularly from a systems-level perspective.

Given the reported multi-target activity of hesperidin and the highly interconnected nature of the ovarian TME, a systems-level modelling approach may provide insights that cannot be readily obtained from isolated experimental observations. Therefore this work develops an ODE-based framework of the ovarian cancer microenvironment incorporating tumour cells, immune subsets, CAFs, ECM dynamics, vasculature, metabolic regulation and redox signalling together with the reported modulatory actions of hesperidin. The framework was designed to explore how perturbations induced by hesperidin may propagate through interconnected biological subsystems and influence overall tumour ecosystem behaviour [21]. The objective of the present study was not to develop a quantitatively calibrated predictive model of ovarian cancer progression, but rather to construct a mechanistic framework integrating key tumour–immune–stromal interactions and reported biological effects of hesperidin. Consequently, the simulations should be interpreted as exploratory and hypothesis-generating, providing qualitative insights into potential mechanisms through which hesperidin may modulate the ovarian TME. Rather than representing a PA-1 monoculture, the proposed framework models a hypothetical ovarian TME centred on PA-1 tumour cells and incorporates key immune, stromal, angiogenic, metabolic and ECM interactions characteristic of ovarian cancer progression.

The present study combines mechanistic modelling and systems-level analysis to investigate how hesperidin-mediated perturbations may influence interconnected tumour, immune, stromal, metabolic, angiogenic and redox processes within an ovarian cancer microenvironment. The manuscript is organized as follows. Section 2 describes the mathematical framework, parametrization strategy, scaling assumptions and sensitivity-analysis procedures. Section 3 presents the simulation results and identifies the dominant mechanisms governing model behaviour. Section 4 discusses the biological interpretation of the findings, their relationship to existing experimental evidence and the limitations of the proposed framework. Finally, §5 summarizes the principal conclusions and outlines directions for future experimental validation and model refinement.

## 2. Material and methods

### 2.1. Modelled ovarian cancer cells

Simulations were based on the adherent PA-1 (CRL-1572) ovarian teratocarcinoma cell line, derived from the ascitic fluid of a 12-year-old patient. PA-1 was selected because it is a highly pluripotent and aggressive germ cell-derived tumour model capable of differentiating into all three germ layers, making it suitable for studying microenvironmental signalling and drug responses in ovarian cancer. The model was developed using PA-1 ovarian cancer cells as a biological reference context for interpretation of simulation outcomes.

The tumour compartment of the model was conceptually linked to PA-1 ovarian cancer cells; however, the framework does not represent a PA-1 monoculture system. Instead, it describes a hypothetical ovarian TME in which PA-1 cells interact with immune, stromal, vascular, metabolic and ECM components represented as aggregate state variables. These additional compartments were included to capture biologically relevant processes known to influence ovarian cancer progression *in vivo*.

### 2.2. Flavonoid: hesperidin

Hesperidin (PubChem CID: 10621), a citrus-derived flavanone glycoside, was modelled owing to its reported pro-apoptotic, anti-inflammatory, antioxidant and anti-angiogenic activities. In the computational framework, hesperidin was represented through a dose-response function D(t), implemented via a Hill equation using ${\text{IC}}_{50}$ and $n_{H}$ parameters. This allowed dose-dependent modulation of immune activity, macrophage polarization, cytokine release, metabolic fluxes and hypoxia-related responses across a typical *in vitro* range (10–100 µM). The hesperidin concentrations investigated in this study (10–100 µM) were selected based on concentration ranges frequently reported in experimental studies evaluating the anti-cancer, antioxidant and microenvironment-modulating effects of hesperidin and related flavonoids. These doses were used to explore the qualitative response of the model across increasing levels of biological modulation rather than to reproduce pharmacokinetically validated tumour exposures.

Hesperidin concentration (D) was treated as a constant model input, representing a fixed exposure condition. Consequently, no pharmacokinetic processes such as absorption, distribution, metabolism, elimination or concentration decay were incorporated into the framework. The investigated concentrations (10–100 µM) therefore correspond to hypothetical exposure levels used to explore qualitative dose-dependent system behaviour.

### 2.3. Simulations

All simulations were performed in MATLAB R2025a (MathWorks, USA) using the adaptive ODE solver ode45 (relative tolerance $10^{-6}$). Baseline conditions corresponded to untreated PA-1 cells. Parameter values were sourced from published *in vitro*/*in silico* literature on tumour–immune–stromal interactions.

### 2.4. Model overview

A mechanistic ODE model was developed to simulate tumour–immune–stromal interactions in the ovarian cancer microenvironment under hesperidin exposure. The model consisted of five coupled modules, each formulated with nonlinear ODEs and Hill-type regulatory kinetics:

— Tumour–immune dynamics.
  Logistic tumour growth with killing by effector T cells (E) and M1 macrophages, and suppression by regulatory T cells (R) and M2 macrophages. Cytokines (IL−10, TGF−β, interferon IFN−γ) and PD−1/PD−L1 checkpoint activity modulate these interactions.
— Macrophage polarization and immunoregulation.
  M1/M2 balance governed by lactate and TGF−β (promoting M2) versus interferon and hesperidin (promoting M1), with feedback through cytokine production and NF−κB/STAT1 signalling.
— Stromal and ECM remodelling.
  CAF expansion, ECM deposition and matrix metalloproteinase (MMP)-driven matrix degradation were modelled, with Yes-associated protein 1/Transcriptional coactivator with PDZ-binding motif (YAP/TAZ) mechanotransduction linking tumour pressure to fibroblast activation and stiffness changes.
— Hypoxia and angiogenesis.
  O${}_{2}$-dependent activation of HIF−1α, VEGF-mediated angiogenic signalling and vascular expansion (V), influencing oxygen and nutrient delivery.
— Metabolic and redox control.
  Glucose uptake, lactate production and NRF2 antioxidant responses, with hesperidin implemented as a modulator of ROS generation and glycolytic flux.

By integrating PD−1/PD−L1 immune regulation, YAP/TAZ mechanotransduction, NRF2 redox control, TGF−β immunosuppression and HIF−1α/VEGF angiogenesis, the proposed model captures multi-scale crosstalk in ovarian tumour progression and enables simulation of hesperidin-induced microenvironmental reprogramming.

The selection of functional forms represented a compromise between biological realism and model complexity. Saturating terms were employed where biological evidence strongly supported bounded responses, whereas linear or mass-action interactions were retained as phenomenological approximations for system-level regulatory processes. This approach reduced the number of additional parameters requiring estimation while preserving the principal biological mechanisms investigated in the study.

All simulations were performed over a 200-hour time horizon, which was selected to provide a common observation window allowing simultaneous evaluation of tumour, immune, stromal, angiogenic, metabolic and redox responses to hesperidin exposure. The selected time horizon was consistent with typical *in vitro* exposure studies of natural compounds, which commonly evaluate biological responses over 24–240 hours.

For clarity, table 6 in appendix A lists an overview of all state variables and auxiliary signals used throughout the model, including their biological meaning and scaling conventions.

**Table 1.** Tumour growth and immune-cell range of initial parameters used in the ODE-based framework.

parameter	description	units	range	evidence source
$r_{T}$	tumour intrinsic growth rate	h${}^{-1}$	0.02–0.05 [35,36]	mechanistic modelling, ovarian
$K_{T}$	tumour carrying capacity	cells	$10^{6}$–$10^{9}$ [37]	mechanistic modelling
$k_{E}$	effector T-cell killing rate	h${}^{-1}$ cells${}^{-1}$	0.1–1 [38]	mechanistic + B16F10 validation
$K_{E}$	half-saturation constant for T-cell killing	cells	$10^{4}$–$10^{6}$ [37]	mechanistic modelling
$s_{E}$	basal effector T-cell influx	cells h${}^{-1}$	10–100 [39]	mechanistic modelling
$d_{E}$	effector T-cell decay rate	h${}^{-1}$	0.005–0.02 [40]	mechanistic modelling
$s_{R}$	basal Treg influx rate	cells h${}^{-1}$	1–20 [41]	mechanistic modelling, ovarian
$a_{R}$	cytokine-driven Treg activation	h${}^{-1}$	0.1–1 [42,43]	mechanistic modelling
$s_{M1}$	basal M1 macrophage influx	cells h${}^{-1}$	5–20 [44]	mechanistic, tumour biology
$d_{M1}$	M1 decay rate	h${}^{-1}$	0.01–0.05 [44]	mechanistic, tumour biology
$s_{M2}$	basal M2 macrophage influx	cells h${}^{-1}$	5–20 [44]	mechanistic, tumour biology
$d_{M2}$	M2 decay rate	h${}^{-1}$	0.01–0.05 [44]	mechanistic, tumour biology

*Note*: Parameter ranges represent approximate physiological estimates used for baseline calibration.

**Table 2.** Stromal, ECM and angiogenesis-related range of initial parameters.

parameter	description	units	range	evidence source
$s_{F}$	basal CAF source rate	cells h${}^{-1}$	1–10 [45]	mechanistic, tumour biology
$a_{F}$	tumour-driven CAF activation	h${}^{-1}$ cells${}^{-1}$	$10^{-4}$–$10^{-2}$ [46]	ovarian
$d_{F}$	CAF decay rate	h${}^{-1}$	0.01–0.03 [47]	mechanistic, tumour biology
$\beta_{1}$	CAF-driven ECM stiffness deposition	h${}^{-1}$ cells${}^{-1}$	$10^{-3}$–$10^{-1}$ [48]	mechanistic modelling, ovarian
$\beta_{2}$	ECM stiffness positive feedback	h${}^{-1}$	$10^{-4}$–$10^{-2}$ [48]	mechanistic modelling, ovarian
$\mu_{S}$	ECM stiffness decay	h${}^{-1}$	$10^{-3}$–$10^{-1}$ [49]	mechanistic modelling, ovarian
$\gamma_{T}$	tumour-derived MMP production	h${}^{-1}$ cells${}^{-1}$	$10^{-4}$–$10^{-2}$ [50,51]	mechanistic modelling, ovarian
$\gamma_{F}$	CAF-derived MMP production	h${}^{-1}$ cells${}^{-1}$	$10^{-4}$–$10^{-2}$ [45]	mechanistic modelling, ovarian
$\delta_{M}$	MMP decay rate	h${}^{-1}$	$10^{-3}$–$10^{-1}$ [52]	mechanistic, tumour biology
$k_{F}^{\mathrm{HES}}$	hesperidin effect on CAF proliferation	h${}^{-1}$ µM${}^{-1}$	$10^{-5}$–$10^{-3}$ [53]	mechanistic, fibroblast evidence
$k_{H}^{\mathrm{HES}}$	HIF−1α inhibition by HES	h${}^{-1}$ µM${}^{-1}$	$10^{-5}$–$10^{-3}$ [54,55]	mechanistic modelling, ovarian
$k_{V}^{\mathrm{HES}}$	HES-driven angiogenesis inhibition	h${}^{-1}$ µM${}^{-1}$	$10^{-5}$–$10^{-3}$ [14,54,55]	ovarian (pathway support)

*Note*: Parameter ranges represent approximate physiological estimates used for baseline calibration.

**Table 3.** Metabolic and redox-regulation range of initial parameters.

parameter	description	units	range	evidence source
$k_{L}^{\mathrm{HES}}$	HES-mediated lactate suppression	h${}^{-1}$ µM${}^{-1}$	$10^{-5}$–$10^{-3}$ [56]	colorectal/metabolic evidence
$s_{N}$	basal NRF2 synthesis	arb. units h${}^{-1}$	$10^{2}$–$10^{4}$ [57]	ovarian (pathway support)
$d_{N}$	effective NRF2 deactivation rate	h${}^{-1}$	0.01–0.03 [58,59]	mechanistic, tumour biology
$a_{R}^{\mathrm{ROS}}$	ROS-driven NRF2 activation	h${}^{-1}$ (ROS)${}^{-1}$	$10^{-4}$–$10^{-2}$ [57,60]	ovarian (mechanistic/pathway support)
$k_{N}^{\mathrm{HES}}$	HES inhibition of NRF2 overactivation	h${}^{-1}$ µM${}^{-1}$	$10^{-5}$–$10^{-3}$ [61]	mechanistic modelling, ovarian
$R_{O}$	ROS concentration activity level; model state variable	arb. units	– [62]	ovarian
$k_{O}$	antioxidant ROS clearance rate mediated by NRF2	h${}^{-1}$	$10^{-4}$–$10^{-2}$ [63]	mechanistic redox biology (pan-cancer)

*Note*: Parameter ranges represent approximate physiological estimates used for baseline calibration.

**Table 4.** State variables and representative initial conditions used in the ovarian TME model.

variable	description	units/scaling	initial value
T	tumour cell abundance	cells (scaled)	$1\times 10^{5}$
E	effector T-cell abundance	cells (scaled)	$1\times 10^{3}$
R	regulatory T-cell abundance	cells (scaled)	$3\times 10^{2}$
$M_{1}$	M1 macrophage abundance	cells (scaled)	$2\times 10^{3}$
$M_{2}$	M2 macrophage abundance	cells (scaled)	$2\times 10^{3}$
F	CAF abundance	cells (scaled)	$10^{2}$–$5\times 10^{2}$
H	relative HIF-1α activity index	arb. units	0.1
V	relative vasculature index	arb. units	1.0
S	relative ECM stiffness index	arb. units	0.1
M	relative MMP activity	arb. units	1.0
G	relative glucose concentration index	arb. units	1.0
L	relative lactate concentration index	arb. units	0.5
N	relative NRF2 activity index	arb. units	0.2

*Note*: Initial conditions represent biologically plausible baseline states used for exploratory simulations and were not estimated from PA-1-specific experimental measurements. Variables represented as relative biological activities or concentration proxies are reported in arbitrary units (arb. units). Where variables were analysed within separate subsystem simulations, initial values were adjusted to reflect the biological context of the corresponding module.

### 2.5. Tumour compartment

To accurately represent tumour–immune–microenvironment dynamics, the tumour cell population (T) is modelled via logistic growth, immune-mediated cytotoxicity, macrophage subtype contributions and dose-dependent modulation by the natural compound hesperidin. Multi-scale models of tumour–immune interaction are increasingly recognized as essential tools for dissecting microenvironmental regulation [22]:

(2.1) $$\begin{array}{r} \frac{\text{d}T}{\text{d}t}=r_{T}\;T\left(1-\frac{T}{K_{T}}\right)-k_{E}\;D_{\phi }(D)\;\frac{E\;T}{T+K_{E}}-k_{M1T}\;\frac{M_{1}\;T}{T+K_{M1}}+k_{M2T}\;M_{2}T, \end{array}$$

with T: effective scaled abundance of tumour cells; $r_{T}$: intrinsic tumour proliferation rate constant (units: ${\text{h}}^{-1}$); $K_{T}$: carrying capacity of tumour cell population (cells or $\text{cells}\;{\text{volume}}^{-1}$); $k_{E}$: rate constant for effector T-cell mediated tumour cell killing (units: ${\text{h}}^{-1}$); $D_{\phi }(D)$: Hill-type dose-effect function of hesperidin on immune killing (dimensionless; 0–1); E: effective scaled abundance of cytotoxic effector T-cells; $K_{E}$: half-saturation constant for effector T-cell-tumour interaction ($\text{cells}\;{\text{volume}}^{-1}$); $k_{M1T}$: rate constant for M1 macrophage-mediated tumour killing (${\text{h}}^{-1}$); $M_{1}$: effective scaled abundance of M1-polarized macrophages; $K_{M1}$: half-saturation constant for M1 macrophage–tumour interaction ($\text{cells}\;{\text{volume}}^{-1}$); $k_{M2T}$: rate constant representing M2 macrophage promotion of tumour growth (${\text{h}}^{-1}$); $M_{2}$: effective scaled abundance of M2-polarized macrophages.

### 2.6. Effector T-cell dynamics

The effector T-cell population (E) is modelled to capture baseline production, tumour-stimulated proliferation (modulated by hesperidin dose via $D_{\eta }(D)$), natural death and suppression by regulatory T-cells (R) and tumour-mediated checkpoint effects (P). This form builds on established tumour–immune ODE frameworks [23]:

(2.2) $$\begin{array}{r} \frac{\text{d}E}{\text{d}t}\;=\;s_{E}\;+\;p_{E}\;D_{\eta }(D)\;\frac{T}{T+K_{E_{\text{act}}}}\;E\;-\;d_{E}\;E\;-\;\alpha_{R}\;R\;E\;-\;\alpha_{P}\;P\;E \end{array},$$

with E: effective scaled abundance of cytotoxic effector T-cells; $s_{E}$: baseline source rate of effector T-cells ($\text{cells}\;{\text{h}}^{-1}$); $p_{E}$: proliferation rate constant of effector T-cells stimulated by tumour presence (${\text{h}}^{-1}$); $D_{\eta }(D)$: Hill-type function representing hesperidin’s enhancement of effector proliferation (dimensionless); T: tumour cell count (from equation (2.1)); $K_{E_{\text{act}}}$: half-saturation constant for tumour-driven effector activation ($\text{cells}\;{\text{volume}}^{-1}$); $d_{E}$: death (or clearance) rate constant of effector T-cells (${\text{h}}^{-1}$); $\alpha_{R}$: suppression constant representing Treg-mediated inhibition of effector T-cells (${\text{h}}^{-1}\;{\text{cells}}^{-1}$); R: regulatory T-cell count (equation (2.3)); $\alpha_{P}$: suppression constant representing checkpoint-mediated inhibition by tumour/PD-L1 axis (${\text{h}}^{-1}\;{\text{cells}}^{-1}$); P: effective tumour-checkpoint factor (dimensionless or $\text{cells}\;{\text{volume}}^{-1}$) representing PD−1/PD−L1 activity.

### 2.7. Regulatory T-cell (Treg) dynamics

Regulatory T-cells (R) are modelled as being produced at a basal rate and stimulated by suppressive cytokines (C) and lactate accumulation (L), both signatures of the immunosuppressive microenvironment; decay occurs at rate $d_{R}$. This captures how metabolic and cytokine cues shape immune suppression in the TME [24]:

(2.3) $$\begin{array}{r} \frac{\text{d}R}{\text{d}t}\;=\;s_{R}\;+\;a_{R}\;C\;+\;b_{R}\;L\;-\;d_{R}\;R \end{array},$$

with R: effective scaled abundance of regulatory T-cells (Tregs); $s_{R}$: basal source rate of Tregs ($\text{cells}\;{\text{h}}^{-1}$); $a_{R}$: rate constant for cytokine-driven Treg induction (${\text{h}}^{-1}\;{\text{signal}}^{-1}$); C: concentration (or level) of suppressive cytokines (e.g. IL−10, TGF−β) (arbitrary units); $b_{R}$: rate constant for lactate-driven Treg induction (${\text{h}}^{-1}\;{\text{lactate}}^{-1}$); L: lactate concentration (or surrogate thereof) from metabolic module (arbitrary units); $d_{R}$: death (or clearance) rate constant of Tregs (${\text{h}}^{-1}$).

### 2.8. M1 macrophage dynamics

To capture how pro-inflammatory M1 macrophages are generated, function and decline within the TME, the model includes baseline source, effector T-cell induced activation (boosted by the compound dose/D), lactate-mediated inhibition, natural decay and repolarization of M2→M1 induced by treatment. Studies of macrophage plasticity in tumour systems underpin this structure [25]:

(2.4) $$\begin{array}{r} \frac{\text{d}M_{1}}{\text{d}t}=s_{M1}\;+\;a_{M1}^{\mathrm{eff}}(D)\;E\;-\;b_{L1}\;L\;M_{1}\;-\;d_{M1}\;M_{1}\;+\;k_{\mathrm{pol}}(D)\;M_{2} \end{array},$$

with $M_{1}$: effective scaled abundance of M1-polarized macrophages; $s_{M1}$: baseline source/production rate of M1 macrophages ($\text{cells}\;{\text{h}}^{-1}$); $a_{M1}^{\mathrm{eff}}(D)$: effective activation rate of M1 by effector T-cells, modulated by treatment dose D (${\text{h}}^{-1}$); E: effector T-cell count (from equation (2.2)); $b_{L1}$: rate constant for lactate-mediated suppression of M1 macrophages (${\text{h}}^{-1}\;{\text{lactate}}^{-1}$); L: lactate concentration in the microenvironment (arbitrary units); $d_{M1}$: decay/clearance rate constant of M1 macrophages ($h^{-1}$); $k_{\mathrm{pol}}(D)$: rate constant for repolarization of M2→M1, modulated by dose D (${\text{h}}^{-1}$); $M_{2}$: effective scaled abundance of M2-polarized macrophages (from equation (2.5)).

### 2.9. M2 macrophage dynamics

Because tumour-associated macrophages (TAMs) often shift to an immunosuppressive M2 phenotype under conditions of lactate accumulation and suppressive cytokines (C), the model reflects this by including baseline production, dose-modulated lactate and cytokine driving terms, effector T-cell mediated elimination (enhanced by treatment), natural decay and repolarization out of M2 [26]:

(2.5) $$\begin{array}{r} \frac{\text{d}M_{2}}{\text{d}t}=s_{M2}\;+\;b_{L2}^{\mathrm{eff}}(D)\;L\;+\;g_{C2}^{\mathrm{eff}}(D)\;C\;-\;a_{E2}^{\mathrm{eff}}(D)\;E\;M_{2}\;-\;d_{M2}\;M_{2}\;-\;k_{\mathrm{pol}}(D)\;M_{2} \end{array},$$

with $M_{2}$: effective scaled abundance of M2-polarized macrophages; $s_{M2}$: baseline source rate of M2 macrophages ($\text{cells}\;{\text{h}}^{-1}$); $b_{L2}^{\mathrm{eff}}(D)$: effective rate constant of lactate-driven induction of M2, modulated by dose D (${\text{h}}^{-1}$); L: lactate concentration (as above); $g_{C2}^{\mathrm{eff}}(D)$: effective rate constant of cytokine-driven induction of M2, modulated by dose D (${\text{h}}^{-1}$); C: suppressive cytokine concentration (TGF−β, IL−10) (arbitrary units); $a_{E2}^{\mathrm{eff}}(D)$: rate constant of effector T-cell mediated killing or suppression of M2, modulated by dose D (${\text{h}}^{-1}\;{\text{cells}}^{-1}$); E: effector T-cell count; $d_{M2}$: decay/clearance rate constant of M2 macrophages (${\text{h}}^{-1}$); $k_{\mathrm{pol}}(D)$: rate constant for repolarization of M2→M1 (as above).

### 2.10. Hypoxia-induced HIF−1α dynamics

To capture how tumour hypoxia activates HIF−1α (H) and drives downstream angiogenic signalling, this equation models basal production, oxygen-deficit induced activation, natural decay and suppression by hesperidin [27]:

(2.6) $$\begin{array}{r} \frac{\text{d}H}{\text{d}t}=s_{H}+a_{H}\;\frac{O_{0}-O}{O_{0}}\;H\;-\;d_{H}\;H\;-\;k_{H}^{\mathrm{HES}}\;D\;H \end{array},$$

with H: level or concentration of HIF−1α (arbitrary units); $s_{H}$: basal production rate of HIF−1α (units: ${\text{h}}^{-1}$); $a_{H}$: activation rate constant of HIF−1α driven by relative oxygen deficit $\frac{O_{0}-O}{O_{0}}$ ($h^{-1}$); O: local oxygen concentration (units: arbitrary); $O_{0}$: reference (normoxic) oxygen concentration; $d_{H}$: decay rate constant of HIF−1α ($h^{-1}$); $k_{H}^{\mathrm{HES}}$: suppression constant of HIF−1α by hesperidin dose D (${\text{h}}^{-1}\;µ{\text{M}}^{-1}$); D: hesperidin dose (µM).

### 2.11. Angiogenesis/vasculature (V) dynamics

Angiogenic vasculature (V) is stimulated by HIF−1α signalling in response to hypoxia, and is modulated by hesperidin which reduces vessel formation; modelling V enables simulation of treatment-induced vascular changes in the TME [28]:

(2.7) $$\begin{array}{r} \frac{\text{d}V}{\text{d}t}=s_{V}+a_{V}\;H\;-\;d_{V}\;V\;-\;k_{V}^{\mathrm{HES}}\;D\;V \end{array},$$

with V: amount or functional capacity of tumour vasculature (arbitrary units); $s_{V}$: basal source rate of new vasculature (units: ${\text{h}}^{-1}$); $a_{V}$: rate constant linking HIF−1α (H) to new vessel growth (${\text{h}}^{-1}\;{\text{arb. units}}^{-1}$); H: level of HIF−1α (from equation (2.6)); $d_{V}$: decay or regression rate constant of vasculature (${\text{h}}^{-1}$); $k_{V}^{\mathrm{HES}}$: suppression constant of vasculature formation by hesperidin dose D (${\text{h}}^{-1}\;µ{\text{M}}^{-1}$); D: hesperidin dose (µM).

### 2.12. Cancer-associated fibroblast dynamics

CAFs play a pivotal role in TME remodelling via ECM deposition and signalling feedback; modelling their dynamics allows capturing how the natural compound (hesperidin) influences stromal activation and thus tumour progression. Cells–matrix interplay and CAF-driven stiffness feedback are increasingly highlighted in TME modelling [29,30]:

(2.8) $$\begin{array}{r} \frac{\text{d}F}{\text{d}t}=s_{F}+a_{F}\;T\;-\;d_{F}\;F\;-\;k_{F}^{\mathrm{HES}}\;D\;F \end{array},$$

with F: number or density of CAFs (cells); $s_{F}$: basal source/production rate of CAFs ($\text{cells}\cdot {\text{h}}^{-1}$); $a_{F}$: activation rate constant of CAFs by tumour cells (${\text{h}}^{-1}\;{\text{cells}}^{-1}$); T: tumour cell count from tumour compartment; $d_{F}$: decay or clearance rate constant of CAFs (${\text{h}}^{-1}$); $k_{F}^{\mathrm{HES}}$: effectiveness constant of hesperidin dose D in suppressing CAF proliferation (${\text{h}}^{-1}\;µ{\text{M}}^{-1}$); D: dose (µM) of hesperidin.

### 2.13. Extracellular matrix stiffness (S) dynamics

ECM stiffness is a physical hallmark of the TME that arises from CAF-mediated matrix deposition and cross-linking, and influences tumour behaviour through mechanotransduction (YAP/TAZ). Modelling S allows the integration of mechanical feedback within the model [30]:

(2.9) $$\begin{array}{r} \frac{\text{d}S}{\text{d}t}=\beta_{1}\;F+\beta_{2}\;F\;S\;-\;\mu_{S}\;S \end{array},$$

with S: ECM stiffness index (arbitrary units); $\beta_{1}$: rate constant linking CAF presence (F) to new matrix deposition (${\text{h}}^{-1}\;{\text{cells}}^{-1}$); $\beta_{2}$: positive feedback coefficient representing stiffness-enhanced further deposition (${\text{h}}^{-1}\;{\text{arb. units}}^{-1}$); $\mu_{S}$: natural relaxation or degradation rate of stiffness (${\text{h}}^{-1}$).

### 2.14. Matrix metalloproteinase activity (M) dynamics

MMPs produced by tumour cells and CAFs facilitate ECM degradation and remodelling, thereby affecting matrix stiffness, invasion capacity and mechanical feedback in tumour–stromal interactions. Modelling M is essential for capturing stromal–tumour crosstalk dynamics [30]:

(2.10) $$\begin{array}{r} \frac{\text{d}M}{\text{d}t}=\gamma_{T}\;T+\gamma_{F}\;F\;-\;\delta_{M}\;M \end{array},$$

with M: MMP activity level (arbitrary units); $\gamma_{T}$: rate constant of tumour-cell induced MMP production (${\text{h}}^{-1}\;{\text{cells}}^{-1}$); $\gamma_{F}$: rate constant of CAF-induced MMP production (${\text{h}}^{-1}\;{\text{cells}}^{-1}$); $\delta_{M}$: decay or clearance rate constant of MMPs (${\text{h}}^{-1}$).

### 2.15. Glucose dynamics (G)

Tumour cells and effector immune cells compete for glucose in the TME, and modelling glucose (G) dynamics—including tumour uptake, immune cell uptake and basal supply/clearance—is vital for capturing metabolic crosstalk and resource limitation as part of the ODE framework [31]:

(2.11) $$\begin{array}{r} \frac{\text{d}G}{\text{d}t}=s_{G}-k_{u}\;T\;\frac{G}{G+K_{G}}-k_{c}\;E\;\frac{G}{G+K_{G,E}}-d_{G}\;G \end{array},$$

with G: glucose concentration in the local microenvironment (arbitrary units); $s_{G}$: basal supply rate of glucose (units: $\text{concentration}\;{\text{h}}^{-1}$); $k_{u}$: rate constant of tumour-cell glucose uptake (${\text{h}}^{-1}\;{\text{cells}}^{-1}$); T: tumour-cell count (from equation (2.1)); $K_{G}$: half-saturation constant for tumour glucose uptake (concentration units); $k_{c}$: rate constant of effector T-cell glucose uptake (${\text{h}}^{-1}\;{\text{cells}}^{-1}$); E: effector T-cell count (from equation (2.2)); $K_{G,E}$: half-saturation constant for effector T-cell glucose uptake (concentration units); $d_{G}$: natural clearance/degradation rate of glucose (${\text{h}}^{-1}$).

### 2.16. Lactate dynamics (L)

Lactate (L) accumulation is a hallmark of the glycolytic shift (Warburg effect) in tumours, and acts as both a metabolic waste product and a signalling molecule driving immunosuppression and stromal reprogramming. Capturing lactate production by tumour cells and CAFs, as well as its clearance and modulation by hesperidin (HES), is essential for representing metabolic–immune–stromal interplay in the framework [32]:

(2.12) $$\begin{array}{r} \frac{\text{d}L}{\text{d}t}=\alpha_{T}\;T\;\frac{G}{G+K_{L}}+\alpha_{F}\;F-d_{L}\;L-k_{L}^{\mathrm{HES}}\;D\;L \end{array},$$

with L: lactate concentration in the microenvironment (arbitrary units); $\alpha_{T}$: rate constant of tumour-cell lactate production per glucose uptake (${\text{h}}^{-1}\;{\text{cells}}^{-1}$); T: tumour-cell count; G: glucose concentration; $K_{L}$: half-saturation constant for glucose to lactate conversion (concentration units); $\alpha_{F}$: rate constant of lactate production by CAFs (${\text{h}}^{-1}\;{\text{cells}}^{-1}$); F: CAF count (from equation (2.8)); $d_{L}$: natural clearance/decay rate of lactate (${\text{h}}^{-1}$); $k_{L}^{\mathrm{HES}}$: effectiveness constant of hesperidin dose D in increasing lactate clearance or reducing production (${\text{h}}^{-1}\;µ{\text{M}}^{-1}$); D: dose (µM) of hesperidin treatment.

### 2.17. NRF2-mediated redox regulation (N)

NRF2 is a key transcription factor regulating cellular antioxidant responses. It is activated under elevated ROS and remains low under balanced redox conditions. In the model, NRF2 dynamics represents redox regulation within the TME, with hesperidin implemented as a dose-dependent inhibitor that reduces excessive NRF2 activation and reflects its antioxidant and chemosensitizing activity [33,34]:

(2.13) $$\begin{array}{r} \frac{\text{d}N}{\text{d}t}=s_{N}+\alpha_{\;\text{ROS}}\;\frac{O}{R+K_{O}}-d_{N}\;N-k_{N}^{\mathrm{HES}}\;D\;N \end{array}$$

with N: NRF2 activity or nuclear concentration (arbitrary units); O: ROS level (arbitrary units); $s_{N}$: basal synthesis rate of NRF2 (arb. units ${\text{h}}^{-1}$); $\alpha_{\;\text{ROS}}$: maximum ROS-dependent activation rate of NRF2 (arb. units ${\text{h}}^{-1}$); $K_{R}$: half-saturation constant for ROS-driven NRF2 activation (arbitrary units); $d_{N}$: decay/deactivation rate of NRF2 (${\text{h}}^{-1}$); $k_{N}^{\mathrm{HES}}$: effectiveness constant of hesperidin dose D in suppressing NRF2 hyperactivation (${\text{h}}^{-1}\;µ{\text{M}}^{-1}$); D: hesperidin concentration (µM).

TMEs exhibit episodic oxidative stress: bursts of ROS occur owing to hypoxia–reoxygenation cycles, mitochondrial dysfunction, or immune-cell activity [34]. Cells respond dynamically—ROS rises rapidly, triggering NRF2 activation, which then induces antioxidant genes. Over time, as these defences restore redox balance, ROS levels gradually decline. Therefore, the ROS(t) profile is generated via

(2.14) $$\begin{array}{r} R(t)=\left\{ \begin{array}{clclc} & R_{\text{base}}+\frac{R_{\text{amp}}\;t}{t_{\text{rise}}}, & \; & t\leq t_{\text{rise}}, & \\ & R_{\text{base}}+R_{\text{amp}}\;e^{-(t-t_{\text{rise}})/t_{\text{decay}}}, & \; & t>t_{\text{rise}}, & \end{array}\right. \end{array}$$

with

— $R_{\text{base}}=0.15\to$ basal steady oxidative level in cancer cells,
— $R_{\text{amp}}=0.9\to$ transient stress amplitude, and
— $t_{\text{rise}}=24\;\text{h}$, $t_{\text{decay}}=90\;\text{h}\to$ capture fast onset and slow resolution.

### 2.18. Parameter selection and biological context

Model parameters were selected to represent the principal biological processes governing ovarian cancer progression and TME dynamics, including tumour growth, immune-cell recruitment and regulation, macrophage polarization, CAF activation, ECM remodelling, angiogenesis, metabolic adaptation and oxidative stress responses. Parameter values and admissible ranges were obtained from published experimental and computational studies describing ovarian cancer biology whenever available. For biological processes lacking ovarian cancer-specific quantitative estimates, parameter ranges were derived from mechanistic studies of conserved tumour pathways or established systems biology models and were used as biologically plausible approximations. The PA-1 ovarian cancer cell line was selected as a biologically relevant reference context because it is widely used in studies investigating ovarian cancer metabolism, oxidative stress and responses to natural compounds, including flavonoids. Where available, biological observations reported for PA-1 cells informed model assumptions and the qualitative interpretation of simulation outcomes. However, the model was not calibrated against a dedicated PA-1 experimental dataset, and no formal parameter estimation procedure was performed using PA-1-specific measurements. Consequently, the proposed framework should be regarded as a mechanistic, hypothesis-generating model rather than a quantitatively calibrated predictive model of PA-1 biology.

To improve transparency, the origin of supporting evidence for each parameter is listed in tables 1, 2 and 3, distinguishing ovarian cancer-specific evidence from mechanistic pathway studies and parameters adopted from broader tumour biology literature. Furthermore, the robustness of model predictions to parameter uncertainty was systematically evaluated using one-at-a-time (OAT) and global Sobol’ sensitivity analyses.

The biological effects of hesperidin incorporated into the model were not assumed to have equal levels of experimental support. Some mechanisms are supported by studies performed in ovarian cancer models, whereas others were inferred from evidence obtained in different tumour types or from general cancer biology literature. To explicitly document these assumptions, table 5 in appendix A lists a structured summary of the hesperidin-dependent mechanisms incorporated into the model, their biological interpretation, supporting references and evidence level. When multiple literature values were available, representative mid-range values were selected for baseline simulations, while reported ranges were used for sensitivity analyses.

The model was developed as a mechanistic systems-level framework and was not calibrated against a dedicated PA-1 experimental dataset. Parameter values were selected from published literature and constrained within biologically plausible ranges. Therefore, the model was intended to investigate qualitative system behaviour and mechanistic interactions rather than to reproduce specific quantitative experimental measurements. The resulting simulations should be interpreted as hypothesis-generating predictions requiring future experimental validation.

All state variables were scaled to biologically plausible ranges and analysed in relative units; therefore, model output should be interpreted as relative trends rather than absolute physiological quantities.

Representative initial conditions used in the simulations are summarized in table 4.

### 2.19. Variable scaling and units

The proposed framework integrates biological processes operating at different organizational levels, including tumour growth, immune-cell regulation, stromal remodelling, angiogenesis, metabolism and oxidative-stress responses. Consequently, the model was formulated as a semi-quantitative mechanistic system rather than a fully dimensional biophysical model. The primary objective was to capture biologically plausible interactions and relative system dynamics rather than to reproduce absolute physiological quantities.

TState variables were grouped into four categories according to their biological interpretation. Tumour cells (T), effector T cells (E), regulatory T cells (R), M1 macrophages ($M_{1}$), M2 macrophages ($M_{2}$) and CAFs (F) were represented as effective cell-abundance variables. These variables do not correspond to exact cell counts measured in a specific experimental system but rather to scaled population sizes describing the relative abundance of each cellular compartment.

TGlucose (G) and lactate (L) were represented as relative concentration indices describing the metabolic state of the TME. Similarly, HIF-1α (H), vascularization (V), NRF2 activity (N) and ROS (O) were represented as dimensionless activity variables or biological proxies expressed in arbitrary units. ECM stiffness (S) and MMP activity (M) were modelled as relative biomechanical and remodelling indices reflecting changes in ECM organization rather than directly measured physical quantities.

Because the model combines variables with different biological meanings and scales, the associated parameters should be interpreted as effective interaction strengths and regulatory coefficients rather than directly measurable kinetic constants. The adopted scaling ensures internal consistency of the governing equations and permits comparison of relative changes across biological subsystems under different hesperidin doses. Consequently, model output should be interpreted as qualitative or semi-quantitative trends describing system behaviour rather than absolute biological measurements.

To evaluate the robustness of model predictions despite uncertainty in parameter values and scaling assumptions, both local (OAT) and global (Sobol’) sensitivity analyses were performed.

### 2.20. Sensitivity analysis

To assess the influence of kinetic and drug-modulated parameters on model behaviour, a two-stage global sensitivity workflow was applied. In the first stage, a Morris OAT screening identified key parameters driving the variability of tumour–microenvironment dynamics across physiologically plausible ranges. Each parameter was perturbed within a log-uniform range of ±1 order of magnitude around its nominal value. Elementary effects were computed for 10 to 15 random trajectories with eight discretization levels. The mean absolute elementary effect ($\mu^{*}$) quantified overall parameter influence, while its standard deviation (σ) captured nonlinear and interaction effects [64,65]. Model output included tumour burden ($T_{\text{AUC}}$), immune balance ($M_{1}/M_{2}$), extracellular stiffness ($S_{\text{AUC}}$), hypoxia ($H_{\max}$), angiogenic signalling ($V_{\max}$) and metabolic acidity ($L_{\text{AUC}}$) at 200 hours.

In the second stage, parameters exhibiting the highest $\mu^{*}$ values were further analysed using variance-based Sobol’ sensitivity indices [66,67]. First-order ($S_{i}$) and total-order ($S_{Ti}$) indices were estimated from quasi-Monte Carlo (Saltelli) sampling with 1500–2000 base points (depending on computing cost), quantifying both main and interaction effects. Sensitivity was evaluated for hesperidin concentration (50 µM).

For interpretability, a complementary local elasticity analysis was performed by perturbing each parameter ±5% around its nominal value and computing normalized sensitivities (equation (2.15)):

(2.15) $$\begin{array}{r} S_{p}^{Y}=\frac{\partial \ln Y}{\partial \ln p}\approx \frac{Y(p(1+\epsilon ))-Y(p(1-\epsilon ))}{2\epsilon \;Y(p)},\;\epsilon =0.05, \end{array}$$

where:

— $S_{p}^{Y}$ : normalized local sensitivity (elasticity) of output variable Y with respect to parameter p,
— ln⁡Y and ln⁡p : natural logarithms of Y and p, respectively, converting changes into percentage terms,
— $\frac{\partial \ln Y}{\partial \ln p}$ : the derivative of ln⁡Y with respect to ln⁡p, which gives the proportional change in Y for a proportional change in p,
— Y(p(1+ϵ)) : the model output when parameter p is increased by a small fraction ϵ,
— Y(p(1−ϵ)) : the model output when parameter p is decreased by ϵ, and
— ϵ : a small perturbation size (in the method we chose, ϵ=0.05, ±5%).

To evaluate emergent behaviour arising from interactions among biological subsystems, an integrated response analysis was performed. Representative output from tumour, immune, stromal, angiogenic, metabolic and redox modules were normalized to untreated control values and summarized in a heatmap to assess coordinated system-level responses to increasing hesperidin exposure.

### 2.21. Numerical implementation and reproducibility

All simulations were implemented in MATLAB R2025a (MathWorks, Natick, MA, USA). Time-course simulations were performed over a 200-hour interval for fixed hesperidin exposure levels of 0, 10, 25, 50 and 100 µM. Hesperidin concentration (D) was treated as a constant external model input during each simulation, without pharmacokinetic decay. The baseline time-course simulations for equations (2.1)–(2.13) were solved using the MATLAB ode45 variable-step Runge–Kutta solver. For sensitivity analyses and selected numerically stiffer subsystems, the ode23s solver was used with RelTol=1e-6, AbsTol=1e-9, and, where required, MaxStep=2. The HIF−1α Sobol’ analysis was solved using ode45 with RelTol=1e-7 and AbsTol=1e-10. Local OAT sensitivity analyses were performed by perturbing each parameter by ±5% around its nominal value and calculating the corresponding change in the selected model output. Global Sobol’ sensitivity analyses were performed using Saltelli-type sampling at D=50 µM. The number of base samples ranged from 1600 to 2000 depending on the subsystem that was analysed. Random sampling was initialized using a fixed MATLAB random-number seed, rng(1), to ensure reproducibility.

Parameter values, initial conditions, solver calls, dose definitions and sensitivity-analysis settings are defined directly in the corresponding MATLAB scripts. The full set of ODE scripts for equations (2.1)–(2.13), OAT analyses and Sobol’ analyses is publicly available on Zenodo: http://doi.org/10.5281/zenodo.17664294.

## 3. Results

### 3.1. Simulations

Because the framework was not calibrated to a dedicated experimental dataset, the simulation output presented below should be interpreted as qualitative mechanistic predictions rather than quantitative estimates of treatment efficacy. The aim of this section is therefore not to validate hesperidin activity experimentally, but to examine how the modelled tumour–immune–stromal–metabolic network responds to hesperidin-dependent perturbations and to identify which pathways and parameters most strongly control system behaviour.

Simulation of tumour dynamics (equation (2.1)) demonstrated logistic growth of PA-1 ovarian cancer cells approaching carrying capacity over 200 hours. Without hesperidin, tumour expansion followed baseline proliferative behaviour. Incorporating hesperidin-modulated cytotoxicity through the dose–response term $D_{\phi }(D)$ produced a clear, dose-dependent reduction in tumour size, with 50–100 µM showing the strongest attenuation (figure 1), primarily through enhanced effector T-cell-mediated killing and reduced proliferative drive. The OAT sensitivity analysis (figure 2) identified tumour carrying capacity ($K_{T}$) and intrinsic growth rate ($r_{T}$) as dominant parameters, with $k_{E}$, $\alpha_{\text{kill}}$ and $\beta_{r_{T}}$ exhibiting moderate influence. Sobol’ analysis (figure 3) confirmed that tumour kinetics were governed mainly by $r_{T}$, the Hill coefficient ($n_{H}$) and $K_{T}$, while immune- and hesperidin-related parameters contributed moderately. The small gap between first- and total-order indices indicated weak parameter interactions and stable model behaviour. The results suggest that even modest pharmacodynamic modulation of immune and proliferative parameters by hesperidin can measurably attenuate tumour expansion within the presented model of the ovarian cancer microenvironment.

**Figure 1.**
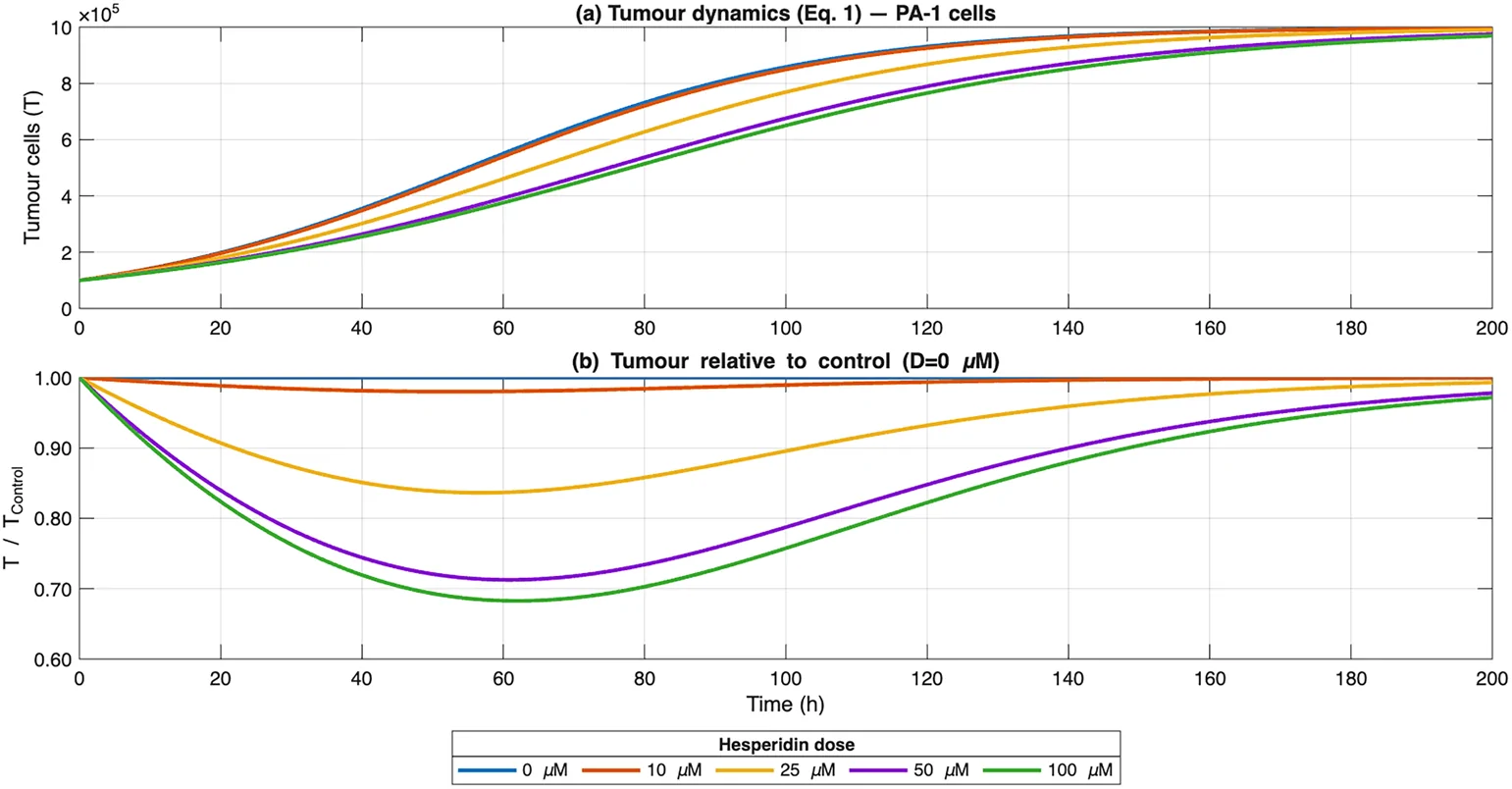
Tumour growth dynamics in the ovarian cancer microenvironment model under hesperidin treatment. (a) Simulated tumour cell dynamics over 200 hours at hesperidin concentrations of 0–100 µM. (b) Tumour size normalized to untreated control (D=0 µM).

**Figure 2.**
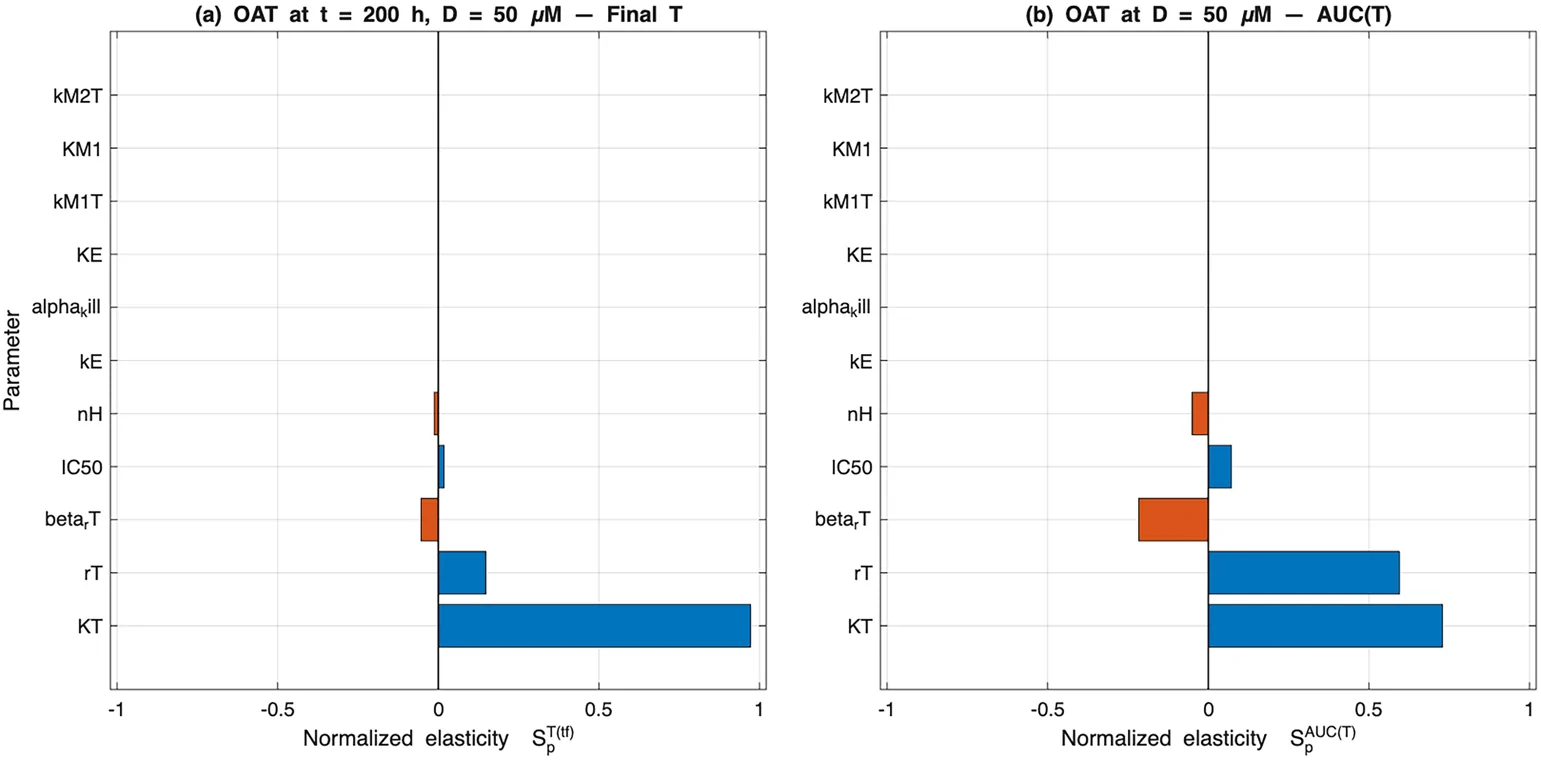
OAT sensitivity analysis of tumour dynamics under hesperidin treatment in PA-1 ovarian cancer cells. Panel (a) shows normalized elasticities $S_{p}^{T(tf)}$ for the final tumour cell count after 200 hours at D=50 µM, while panel (b) presents elasticities $S_{p}^{\text{AUC}(T)}$ for the tumour area under the curve (AUC). Bars show the relative influence of each kinetic or drug-modulated parameter under a ±5% perturbation. Blue bars indicate parameters whose increase amplifies tumour growth, while orange bars indicate those whose increase suppresses it.

**Figure 3.**
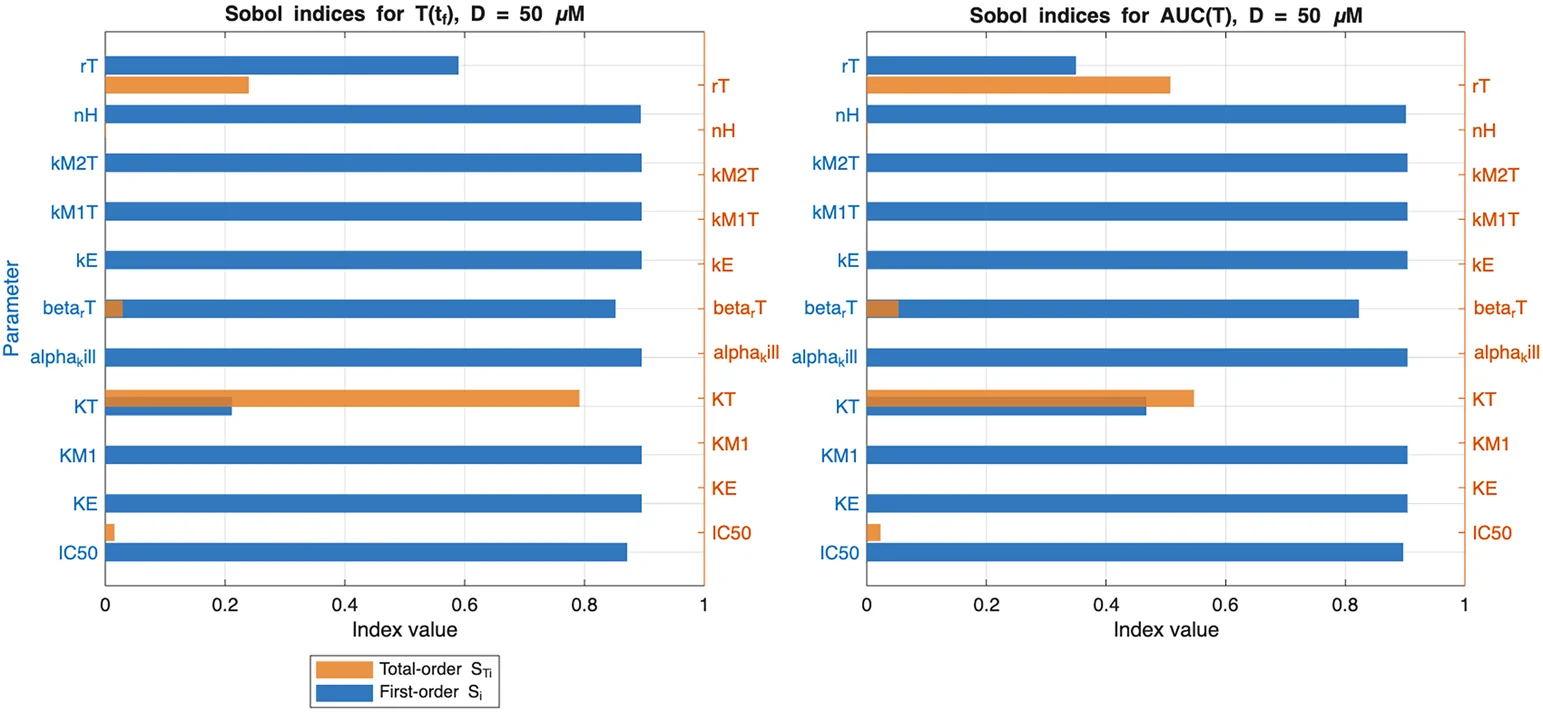
Sobol’ (Saltelli) global sensitivity analysis of tumour dynamics under hesperidin treatment at 50 µM. Panel (a) presents the first-order ($S_{i}$) and total-order ($S_{Ti}$) Sobol’ indices for the final tumour cell count $T(t_{f})$, while panel (b) shows the corresponding indices for the tumour area under the curve (AUC(T)). Blue bars represent main effects ($S_{i}$), and orange bars denote total contributions including parameter interactions ($S_{Ti}$).

Simulation of effector T-cell dynamics (figure 4) showed a clear, dose-dependent immunostimulatory effect of hesperidin. Without treatment, E(t) declined rapidly to a low plateau consistent with T-cell exhaustion under sustained tumour load. The Hill-type function $D_{\eta }(D)$ increased T-cell proliferation in proportion to hesperidin dose, with 50–100 µM producing 1.5–2.5-fold higher E(t) levels than the untreated condition. OAT sensitivity analysis (figure 5) identified recruitment ($s_{E}$) and maximum stimulation ($\eta_{\text{max}}$) as the main positive drivers of effector expansion, while checkpoint-related parameters ($\alpha_{P}$, $P_{\text{chk}}$) exerted the strongest suppressive influence. Sobol’ analysis at 50 µM (figure 6) confirmed that checkpoint modulation and the basal source rate $s_{E}$ dominate effector T-cell behaviour, outweighing intrinsic proliferation terms. The sensitivity structure was consistent across perturbations, indicating stable and robust model dynamics for the tumour–immune module.

**Figure 4.**
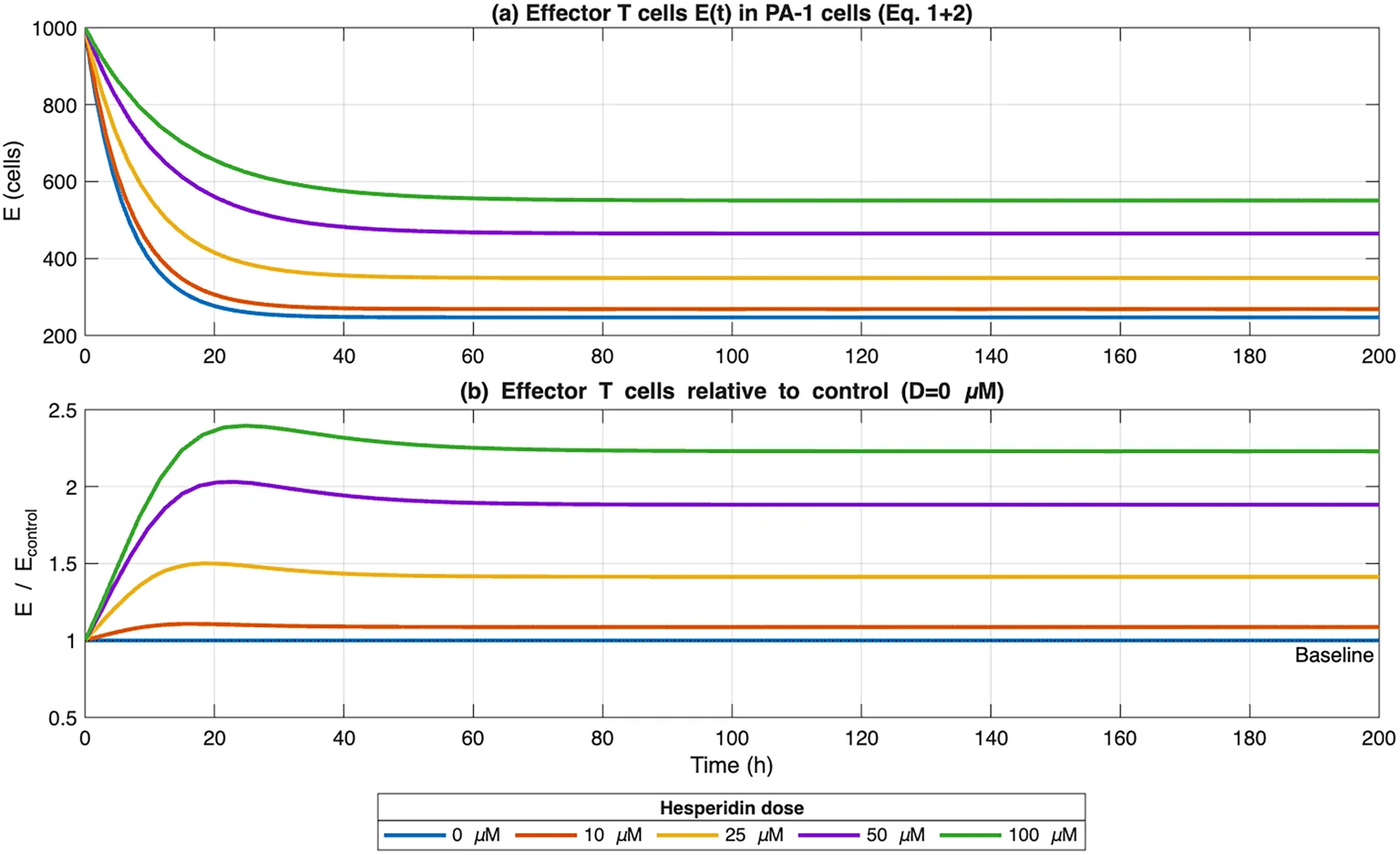
Effector T-cell dynamics under hesperidin modulation in the ovarian cancer microenvironment model. (a) Simulated effector T-cell counts E(t) over 200 hours at hesperidin concentrations of 0–100 µM. (b) Normalized effector T-cell levels ($E/E_{\mathrm{control}}$) relative to untreated control (D=0 µM).

**Figure 5.**
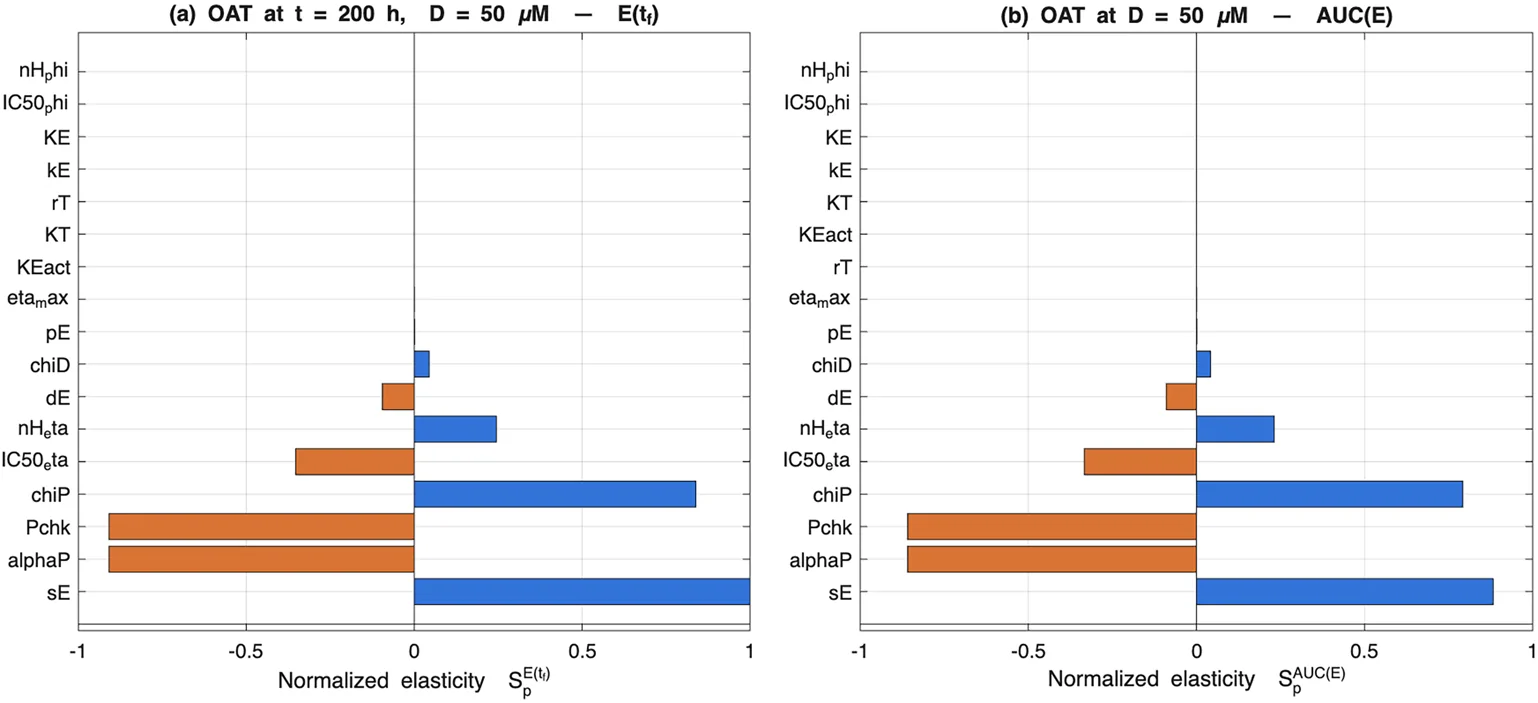
OAT sensitivity analysis for effector T-cell dynamics coupled to tumour growth under 50 µM hesperidin treatment. (a) Normalized elasticities $S_{p}^{E(t_{f})}$ for the final effector T-cell count at 200 hours. (b) Normalized elasticities $S_{p}^{\text{AUC}(E)}$ for the area under the effector T-cell trajectory. Blue bars represent parameters whose increase enhances effector T-cell expansion (positive elasticity), while orange bars correspond to parameters whose increase suppresses effector accumulation (negative elasticity).

**Figure 6.**
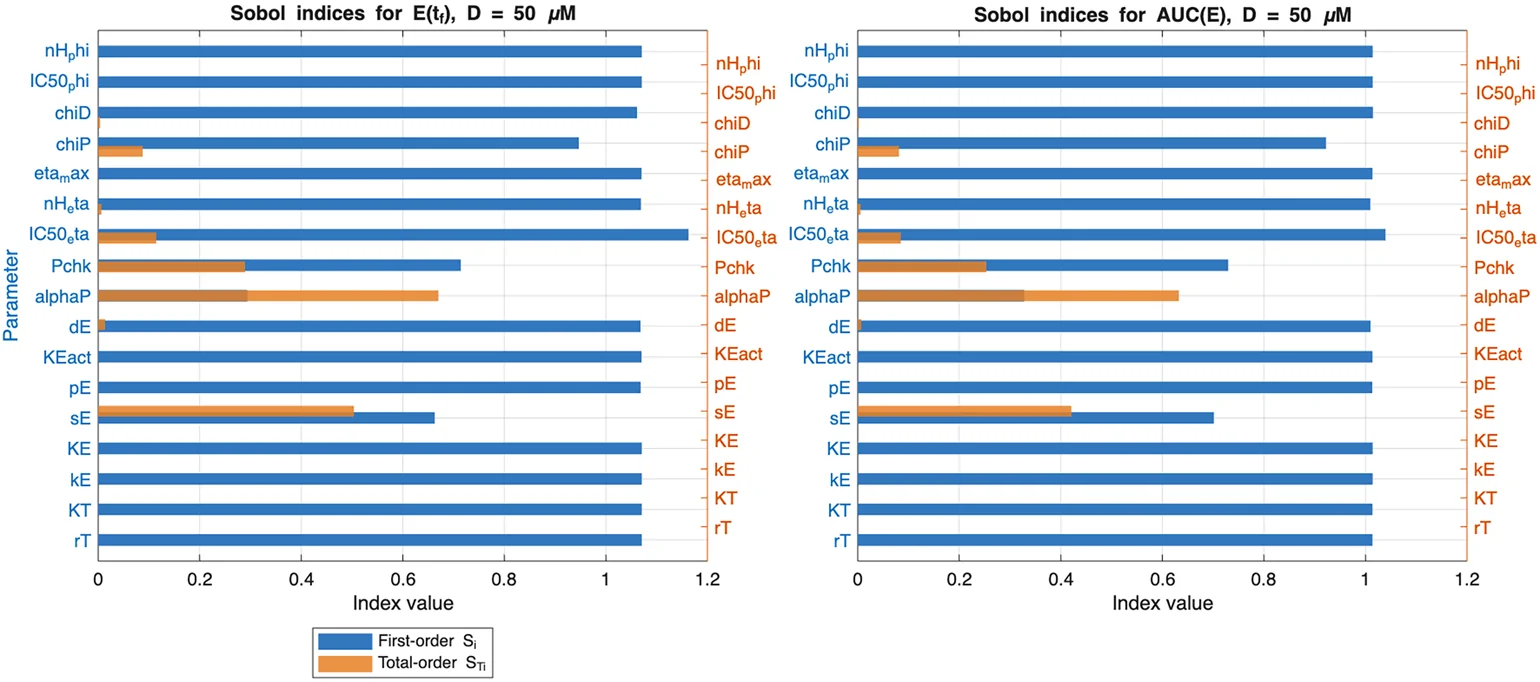
Sobol’ (Saltelli) global sensitivity for effector T-cell dynamics coupled to tumour growth at 50 µM hesperidin. (a) First-order and total-order sensitivity of the terminal effector count $E(t_{\text{end}}$) and (b) the effector exposure AUC(E). Bars show first-order indices $S_{i}$ (blue: main effects) and total-order indices STi (orange: main + interaction effects).

Taken together, the tumour and effector T-cell simulations indicate that tumour suppression in the model is driven primarily by tumour-intrinsic growth kinetics and immune-mediated cytotoxicity, rather than by a single hesperidin-dependent term. Thus, the main mechanistic insight from this module is the relative dominance of $r_{T}$, $K_{T}$, $s_{E}$, checkpoint-related suppression and effector-mediated killing in determining the predicted treatment response.

Simulation of regulatory T-cell dynamics (equation (2.3)) showed a clear dose-dependent reduction in Treg numbers under hesperidin treatment (figure 7). Under control conditions (D=0 µM), R(t) decreased and partially stabilized, whereas increasing hesperidin concentrations (10–100 µM) produced progressively lower steady-state Treg levels. The ratio $R/R_{\text{control}}$ indicated a 15–25% reduction at 50–100 µM. Hesperidin attenuated cytokine-driven activation ($a_{R}C$) and lactate-induced stimulation ($b_{R}L$), leading to reduced effective Treg input and lower R(t) across doses, consistent with diminished immunosuppressive signalling and an environment favouring effector activity (equation (2.2)). The predicted approximately 15--20% decrease in Tregs at high concentrations suggests hesperidin may relieve immunosuppression through attenuation of TGF−β, IL−10 or metabolic competition for IL−2 and lactate-dependent induction pathways, consistent with literature describing flavonoid-mediated reprogramming of the immunosuppressive microenvironment.

**Figure 7.**
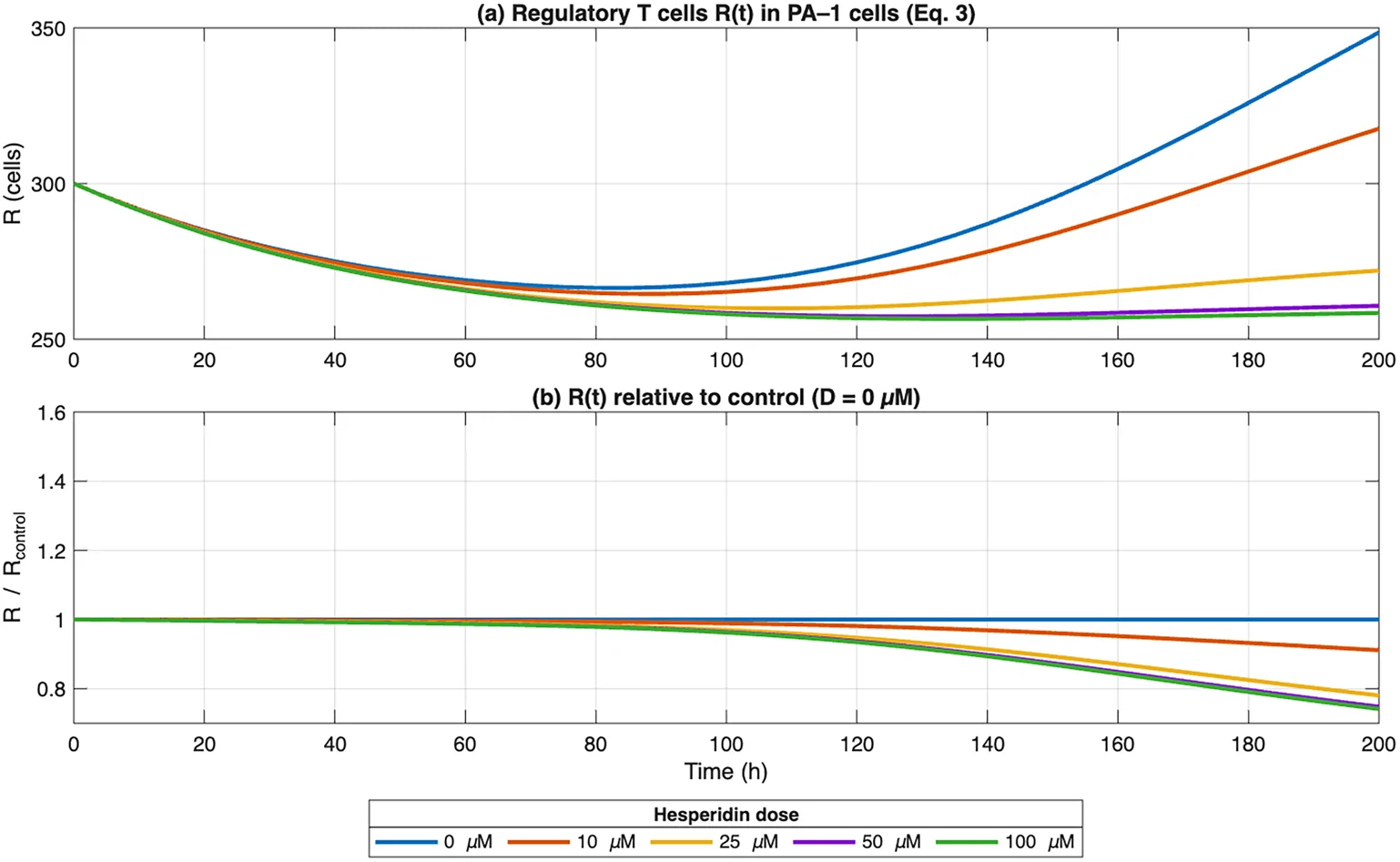
Regulatory T-cell dynamics under hesperidin modulation in the ovarian cancer microenvironment model. (a) Simulated regulatory T-cell populations R(t) over 200 hours at hesperidin concentrations of 0–100 µM. (b) Normalized regulatory T-cell levels ($R/R_{\mathrm{control}}$) relative to untreated control (D=0 µM).

OAT sensitivity analysis (figure 8) identified $d_{R}$, $a_{L}$ and $a_{C}$ as dominant local drivers, with hesperidin-related terms contributing secondarily. Sobol’ analysis at D=50 µM (figure 9) showed that global behaviour was governed primarily by the basal source ($s_{R}$) and decay rate ($d_{R}$), indicating that intrinsic turnover dominates system stability. Overall, tumour metabolic cues ($a_{L}$, $a_{C}$) shape local Treg activation, while $s_{R}$ and $d_{R}$ determine global robustness.

**Figure 8.**
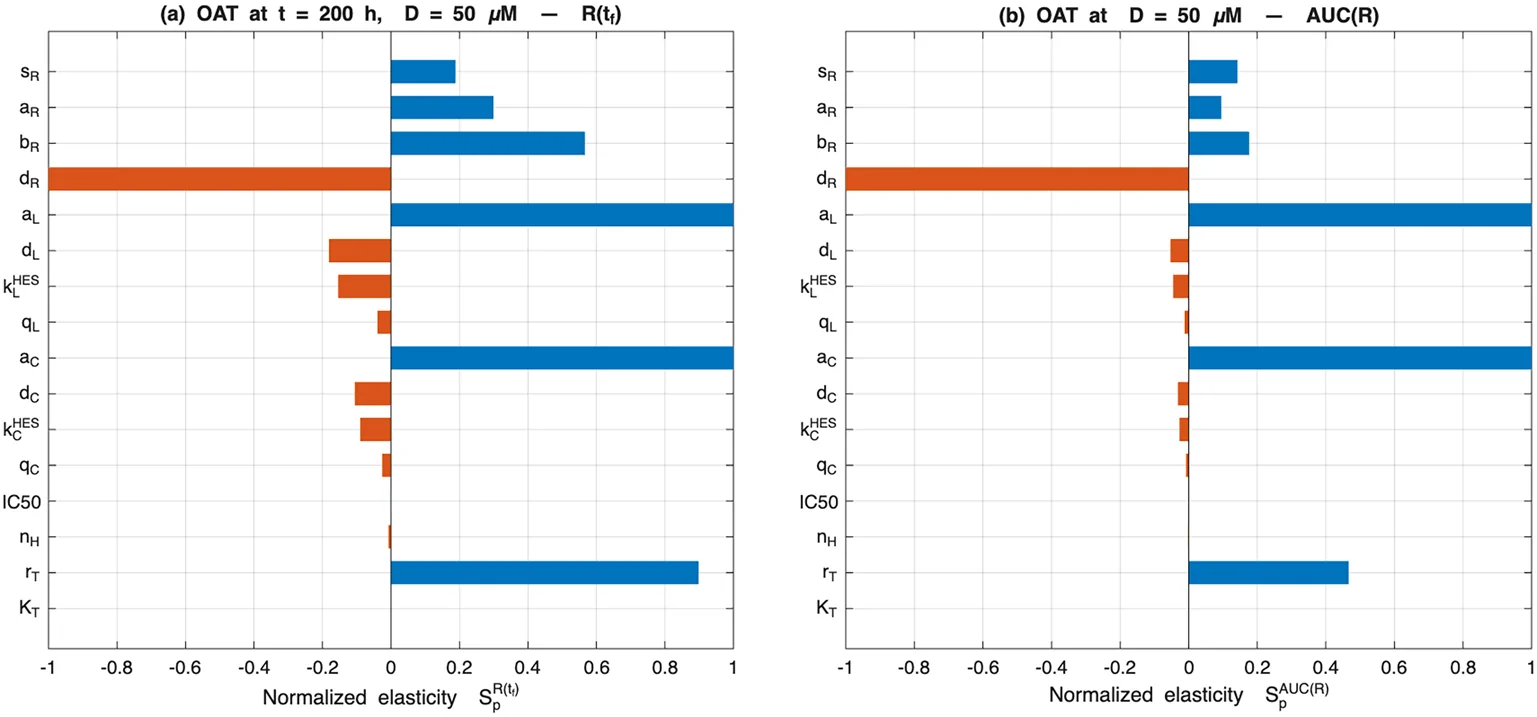
OAT sensitivity analysis of the regulatory T-cell module at D=50 µM. (a) Normalized elasticities $S_{p}^{R(t_{f})}$ for the final regulatory T-cell population $R(t_{f})$ and (b) $S_{p}^{\text{AUC}(R)}$ for the cumulative response AUC(R). Blue bars denote parameters whose increase amplifies R(t) or AUC(R), while orange bars indicate parameters whose increase suppresses output.

**Figure 9.**
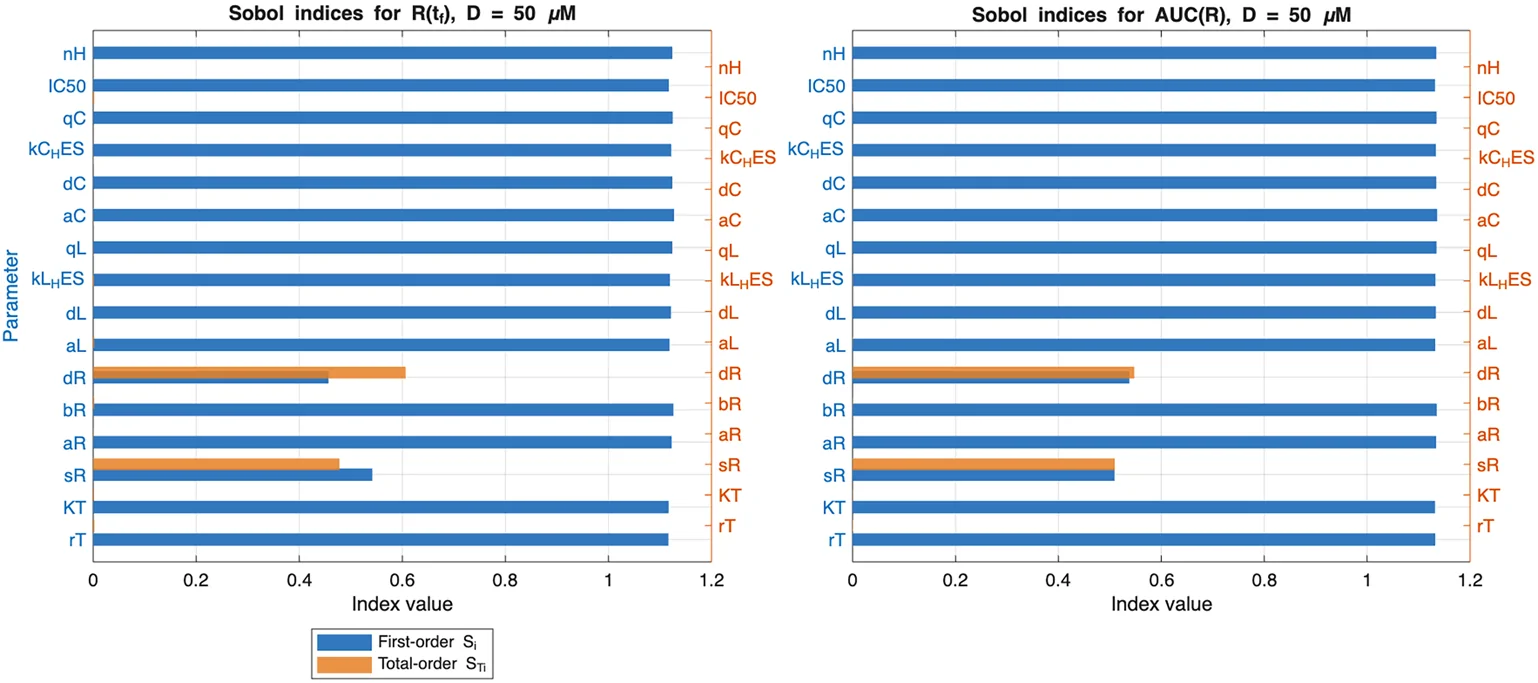
Sobol’ (Saltelli) global sensitivity indices for the regulatory T-cell module under 50 µM hesperidin treatment. (a) Sensitivity of the final regulatory T-cell count $R(t_{f})$ and (b) the total regulatory response represented by AUC(R). Blue bars indicate first-order Sobol’ indices ($S_{i}$) capturing main parameter effects, while orange bars represent total-order indices (STi) including interaction terms.

Simulation of M1 macrophage dynamics (figure 10) showed that untreated $M_{1}(t)$ declined steadily over 200 hours owing to lactate accumulation and cytokine-driven suppression. Hesperidin produced a dose-dependent attenuation of this decline, yielding transient increases and long-term stabilization of $M_{1}$ levels, with 50–100 µM approximately doubling the M1 population relative to control. This effect arose from two mechanisms: enhanced effector T-cell signalling promoting M1 polarization and reduced lactate-mediated inhibition. Together, these shifts counteracted M2-dominated suppression and supported a more immunostimulatory microenvironment. The simulation highlights hesperidin’s potential capacity to promote proinflammatory macrophage polarization and sustain anti-tumour immune activity within the modelling framework. OAT analysis (figure 11) indicated that M1 dynamics was mainly controlled by $a_{M1}$, $b_{L1}$ and $d_{M1}$, with moderate contributions from $k_{\text{pol}0}$ and hesperidin-induced lactate clearance $k_{L}^{\text{HES}}$. Sobol’ analysis (figure 12) confirmed dominant roles for $a_{M1}$, $p_{E}$ and $d_{M1}$, with hesperidin’s modulatory term $\chi_{E}$ providing secondary stabilization. The sensitivity structure remained stable, consistent with robust and physiologically responsive M1/M2 polarization dynamics.

**Figure 10.**
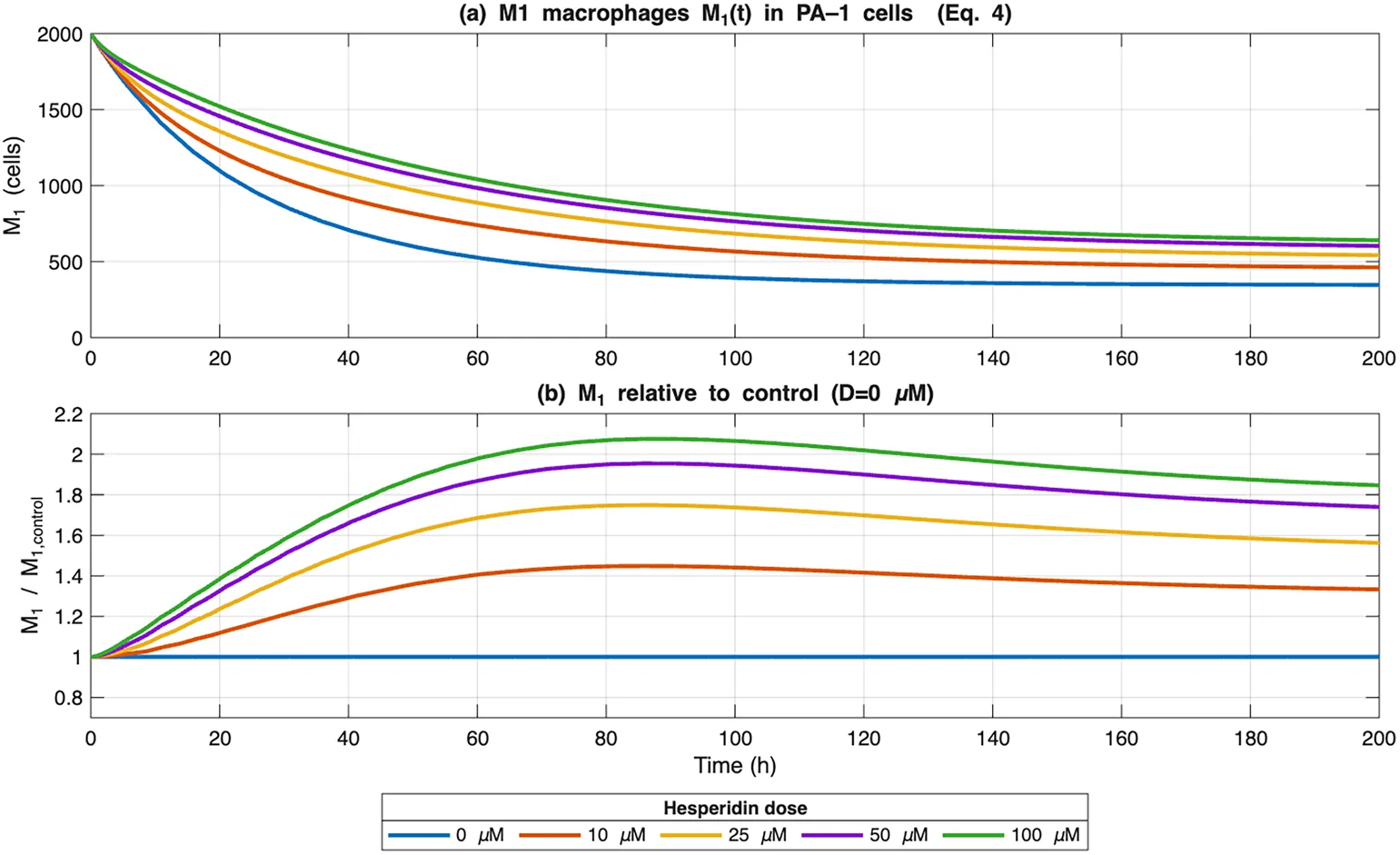
M1 macrophage dynamics under hesperidin modulation in the ovarian cancer microenvironment model. (a) Simulated M1 macrophage populations $M_{1}(t)$ over 200 hours at hesperidin concentrations of 0–100 µM. (b) Normalized M1 macrophage levels relative to untreated control (D=0 µM).

**Figure 11.**
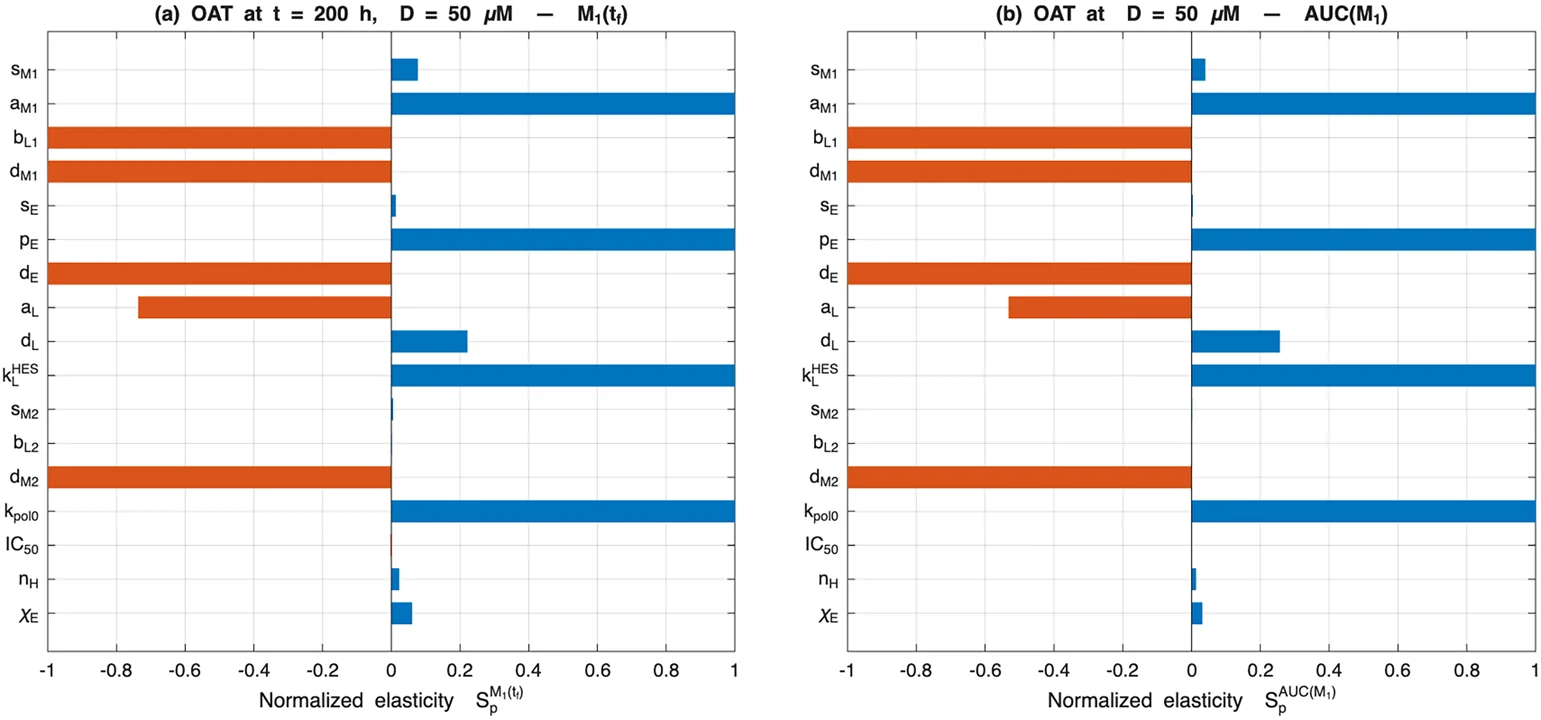
OAT local sensitivity analysis for the M1 macrophage module under D=50 µM hesperidin exposure. (a) Normalized elasticities for the final M1 macrophage count $M_{1}(t_{f})$ and (b) for the integrated macrophage activity $\text{AUC}(M_{1})$. Blue bars represent parameters whose increase enhances M1 accumulation or polarization, whereas orange bars indicate parameters whose increase suppresses it.

**Figure 12.**
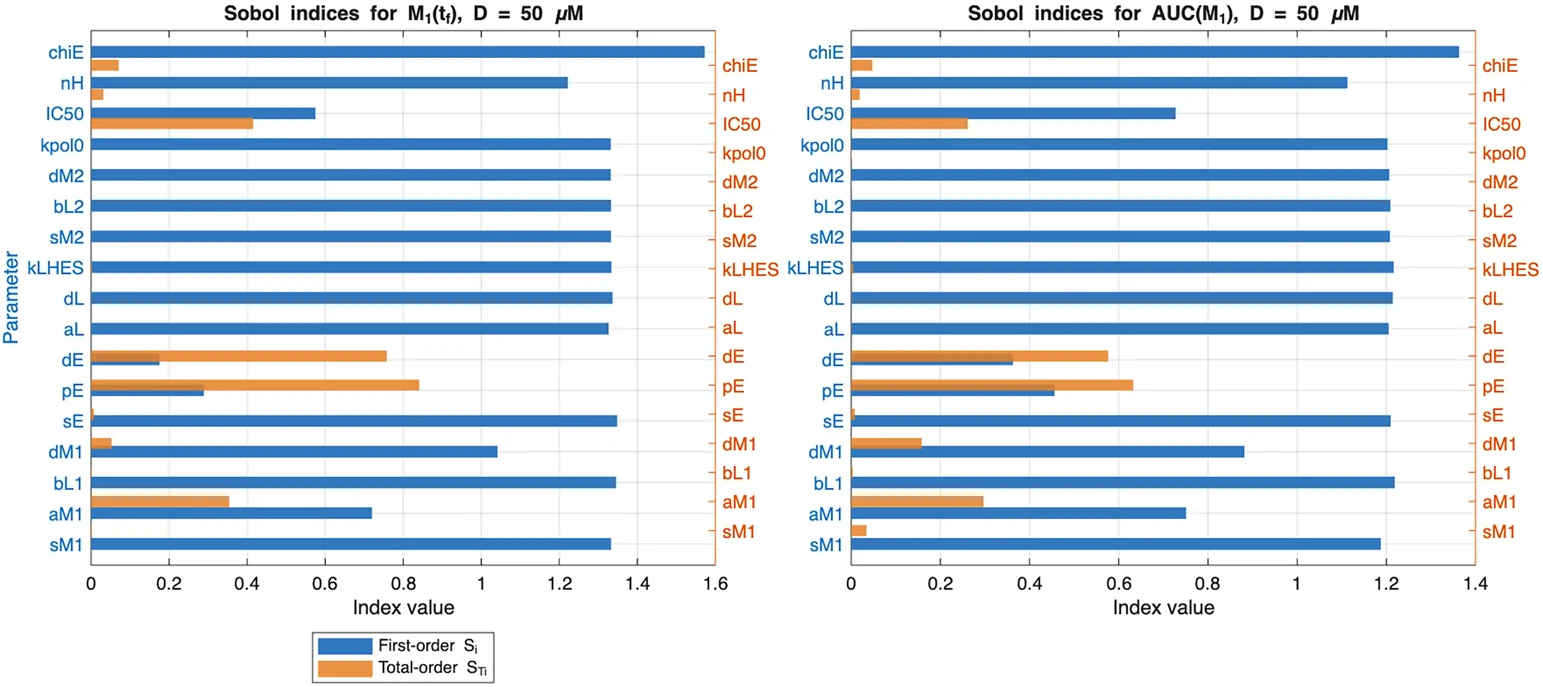
Sobol’ (Saltelli) global sensitivity indices for M1 macrophage dynamics under D=50 µM hesperidin exposure. (a) First-order ($S_{i}$) and total-order ($S_{T_{i}}$) indices for the final macrophage count $M_{1}(t_{f})$ and (b) for the cumulative macrophage activity $\text{AUC}(M_{1})$. Blue bars denote first-order contributions (main effects), while orange bars indicate total-order contributions including parameter interactions.

Simulation of equation (2.5) showed a strong, dose-dependent inhibitory effect of hesperidin on the $M_{2}$ macrophage population (figure 13). Under control conditions (D=0µM), $M_{2}(t)$ declined gradually to a steady state driven by cytokine and lactate inputs balanced by natural decay. With hesperidin, $M_{2}$ numbers decreased more rapidly, with 50–100 µM reducing the population to roughly half of baseline. This reduction resulted from inhibited lactate- and cytokine-mediated polarization and increased effector-mediated elimination along the NF−κB pathway, shifting the microenvironment away from immunosuppressive dominance. This simulation suggests the modelled anti-immunosuppressive effect of hesperidin, reflecting its potential to rebalance macrophage polarization towards a pro-inflammatory M2 phenotype within the TME. OAT analysis (figure 14) indicated that $M_{2}$ behaviour was driven mainly by effector-dependent elimination ($d_{E}$) and basal source ($s_{M2}$), with hesperidin-related polarization ($\chi_{\text{Pol}}$) and clearance terms acting as stabilizing inhibitors. Sobol’ analysis (figure 15) confirmed dominant contributions from $s_{M2}$, $b_{L2}$, $g_{C2}$ and $a_{E2}$, while hesperidin-specific parameters contributed weakly. The sensitivity structure remained stable, indicating a robust and buffered macrophage-regulation subsystem.

**Figure 13.**
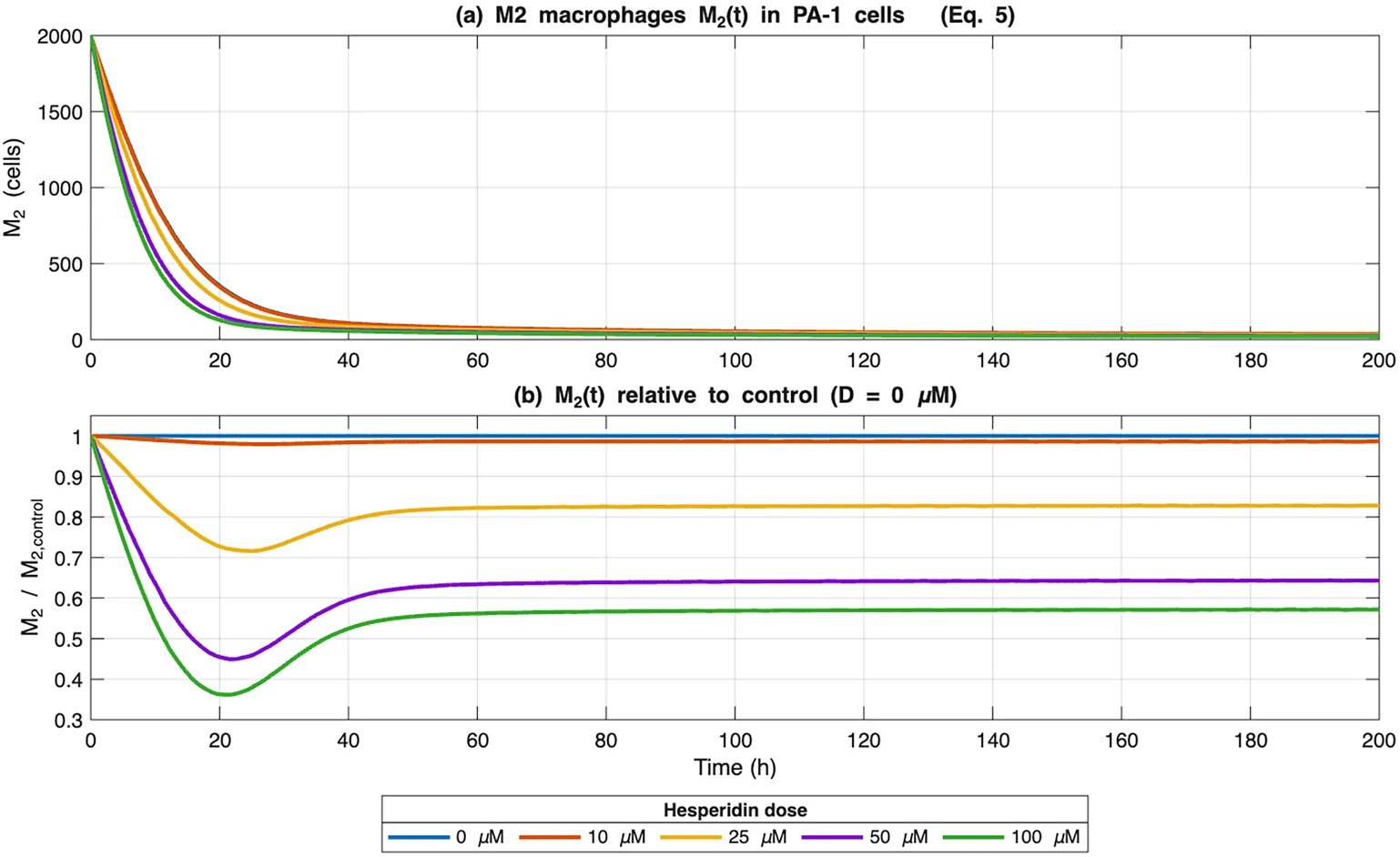
M2 macrophage dynamics under hesperidin modulation in the ovarian cancer microenvironment model. (a) Simulated M2 macrophage populations $M_{2}(t)$ over 200 hours at hesperidin concentrations of 0–100 µM. (b) Normalized M2 macrophage levels relative to untreated control (D=0 µM).

**Figure 14.**
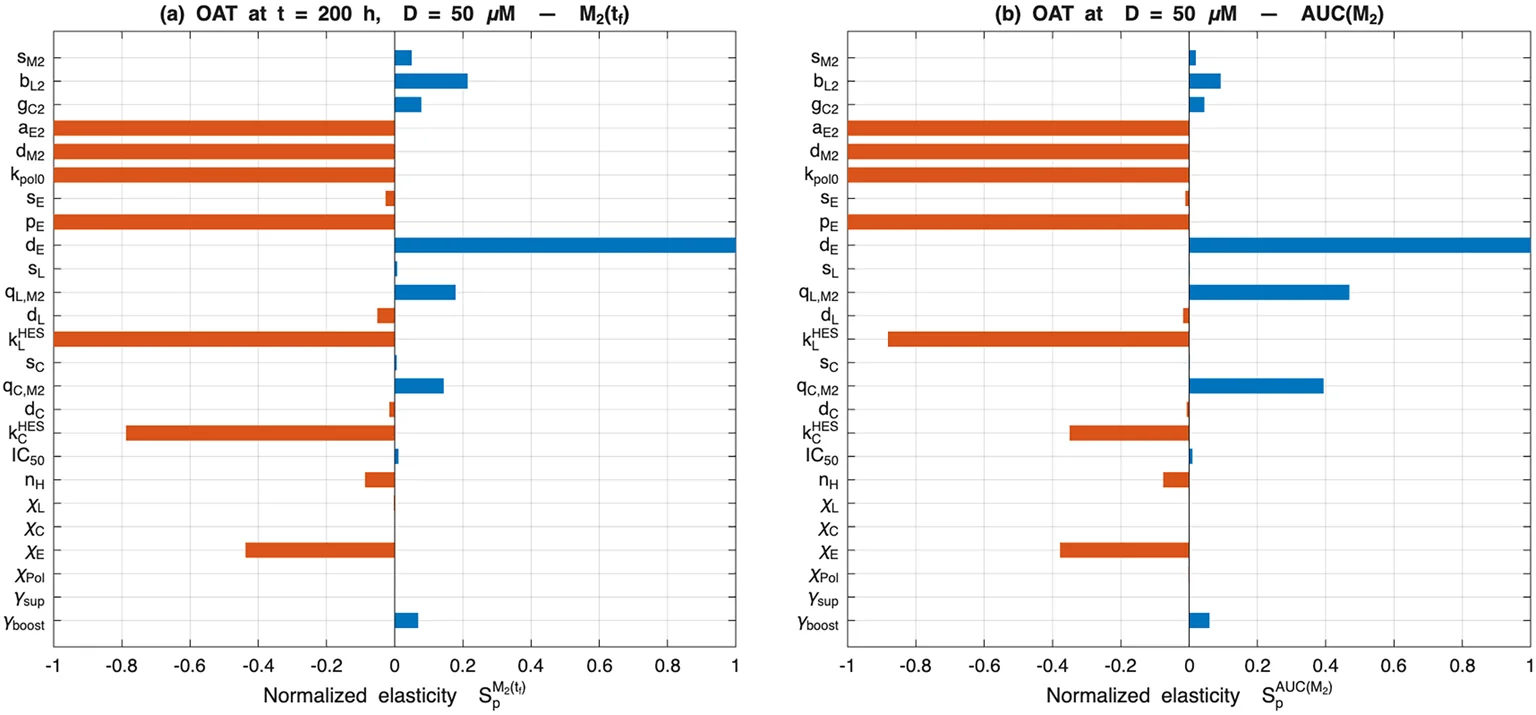
OAT sensitivity analysis of M2 macrophage dynamics under D=50 µM hesperidin exposure. (a) Normalized elasticities $S_{p}^{M_{2}(t_{f})}$ for the final M2 macrophage count and (b) $S_{p}^{\text{AUC}(M_{2})}$ for the cumulative macrophage burden (AUC). Blue bars indicate parameters whose increase promotes M2 accumulation, whereas orange bars denote parameters whose increase suppresses it.

**Figure 15.**
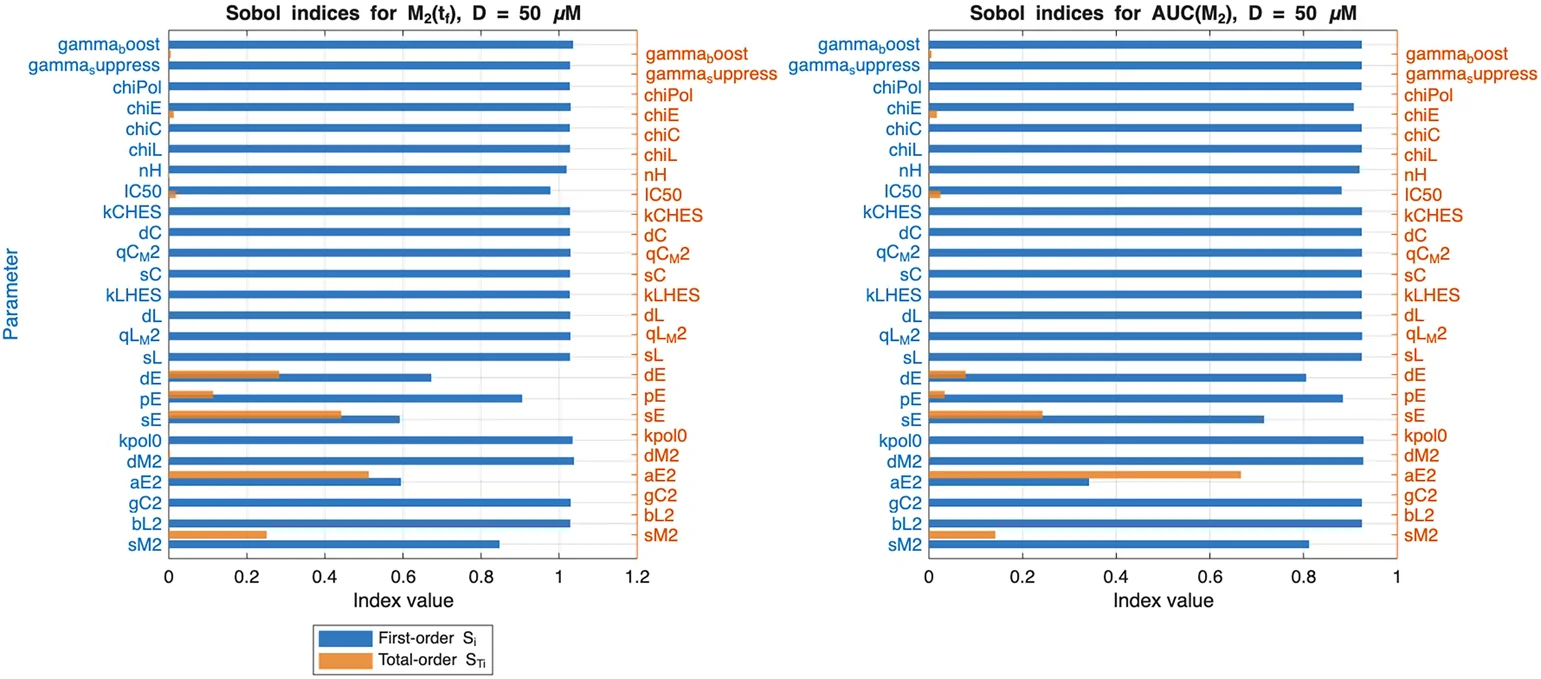
Sobol’ (Saltelli) global sensitivity indices for M2 macrophage dynamics under D=50 µM hesperidin exposure. (a) Sensitivity of the final M2 macrophage count $M_{2}(t_{f})$ and (b) the cumulative macrophage activity ($\text{AUC}(M_{2})$). Bars denote first-order ($S_{i}$, blue) and total-order ($S_{Ti}$, orange) Sobol’ indices, representing main and total (including interaction) parameter effects, respectively.

The immune-subsystem simulations should therefore be interpreted as model-based hypotheses about immune rebalancing rather than direct evidence that hesperidin independently induces these effects in PA-1 cultures. Across the Treg, M1 and M2 modules, the sensitivity analyses indicate that intrinsic immune-cell turnover, lactate/cytokine-driven polarization and effector-mediated suppression are the principal determinants of macrophage and Treg dynamics, whereas hesperidin-dependent terms act mainly as modulators of these pre-existing regulatory axes.

Simulation of equation (2.6) captured the temporal evolution of HIF HIF−1α under varying hesperidin doses (figure 16). In the control condition (D=0 µM), H(t) accumulated continuously owing to oxygen deficit, reflecting strong hypoxia-driven signalling. Increasing hesperidin concentrations progressively reduced HIF−1α levels via the suppression term $k_{H}^{\mathrm{HES}}DH$, yielding lower and smoother trajectories. The strongest attenuation (to approx. 20% of control) occurs at 100 µM hesperidin, indicating potential effective inhibition of hypoxia signalling and downstream reduction of VEGF-driven angiogenic activation within the simulated TME. OAT analysis (figure 17) showed that HIF−1α behaviour is primarily governed by basal production and hypoxic activation, opposed by decay and hesperidin-mediated degradation; the module remained numerically stable and moderately sensitive. Sobol’ analysis (figure 18) confirmed dominant control by hesperidin-driven degradation ($k_{H}^{\mathrm{HES}}$), with minimal contributions from oxygen and production parameters, indicating a well-conditioned, drug-responsive hypoxia subsystem.

**Figure 16.**
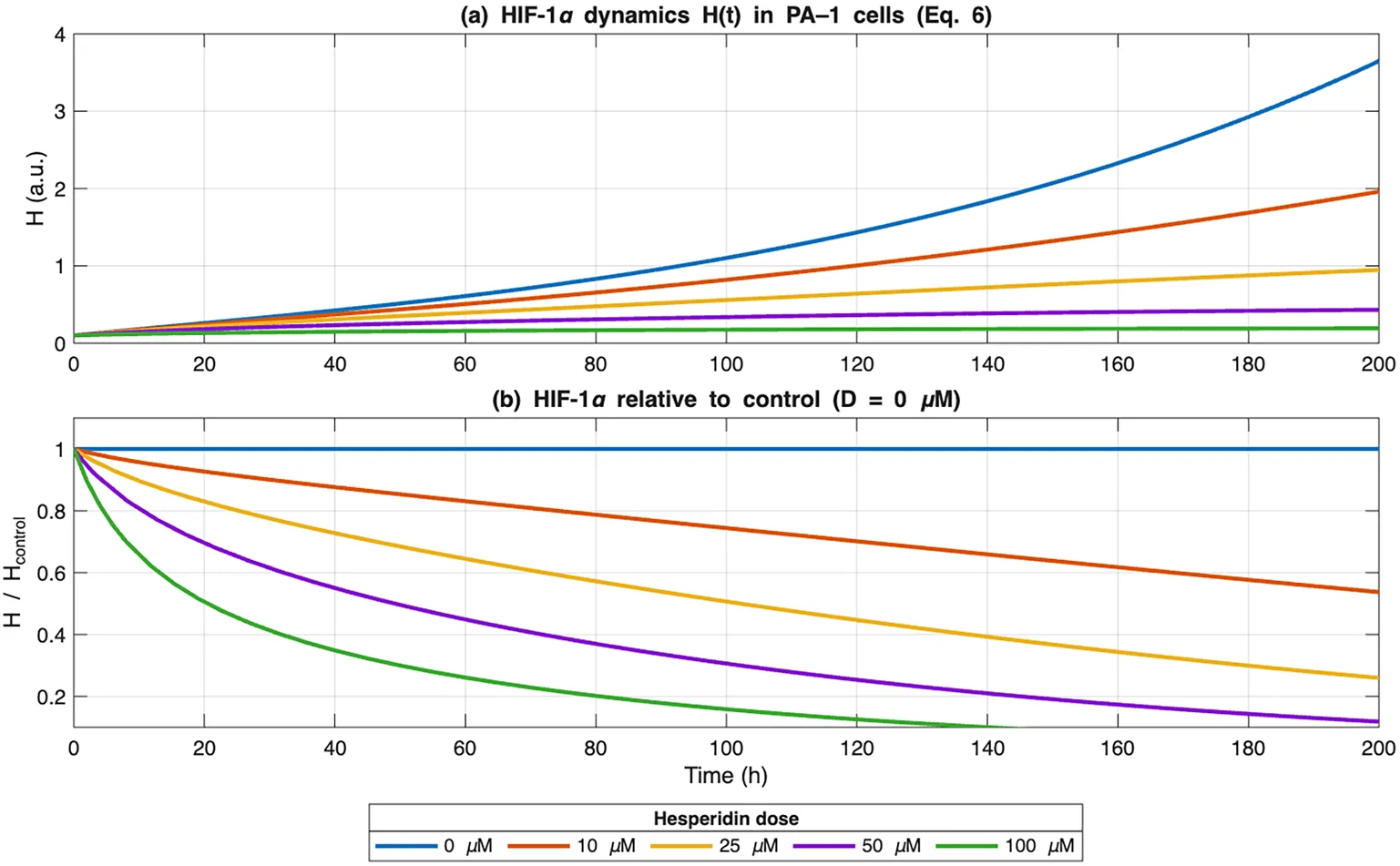
HIF−1α dynamics under hypoxia and hesperidin modulation in the ovarian cancer microenvironment model. (a) Simulated HIF−1α levels H(t) over 200 hours at hesperidin concentrations of 0–100 µM. (b) HIF−1α levels normalized to untreated control (D=0 µM).

**Figure 17.**
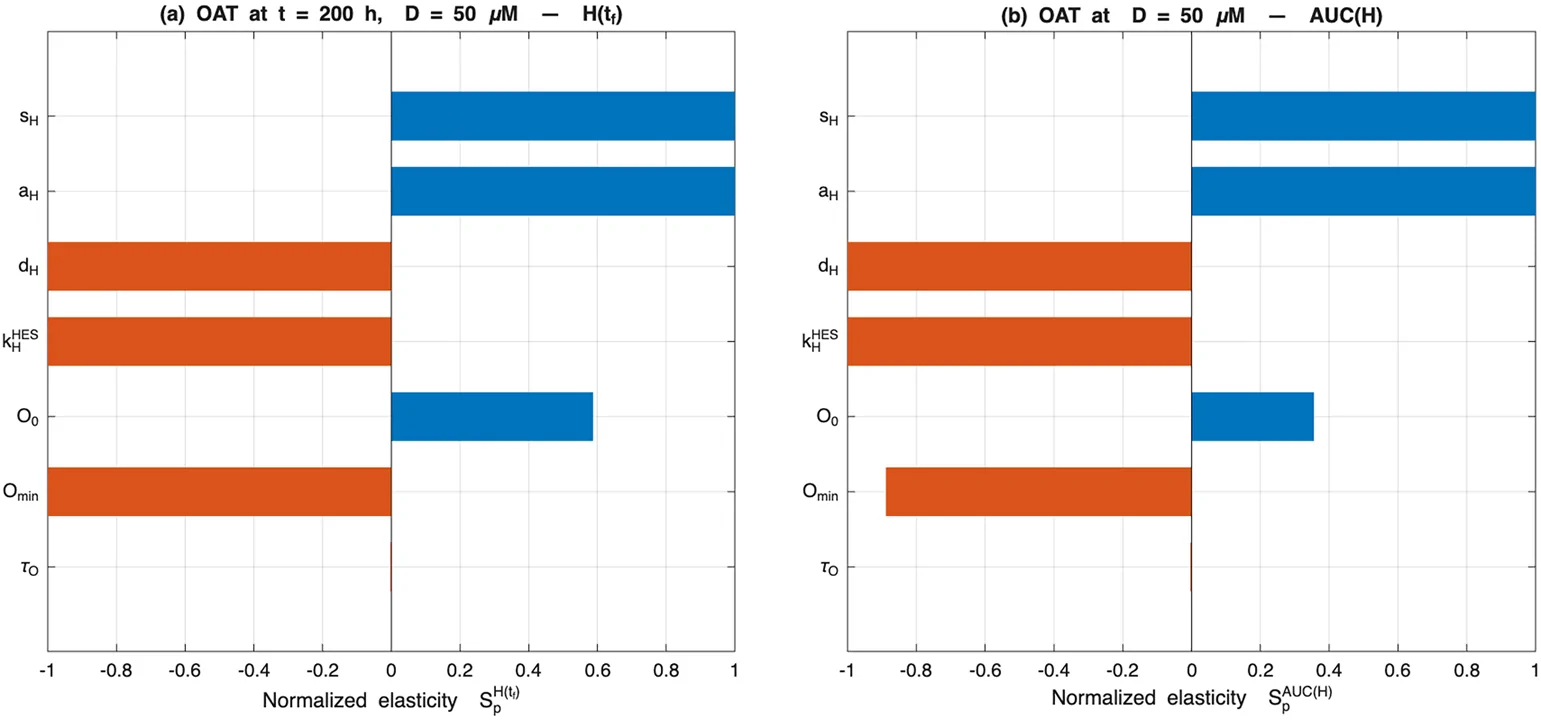
OAT sensitivity analysis of the HIF−1α regulatory model under D=50 µM hesperidin exposure. (a) Normalized elasticities for the final HIF−1α level $H(t_{f})$ and (b) for the cumulative hypoxia signal AUC(H)). Blue bars indicate positive sensitivities ($S_{p}>0$), where an increase in the parameter amplifies the output, whereas orange bars indicate negative sensitivities ($S_{p}<0$), where an increase suppresses HIF−1α accumulation.

**Figure 18.**
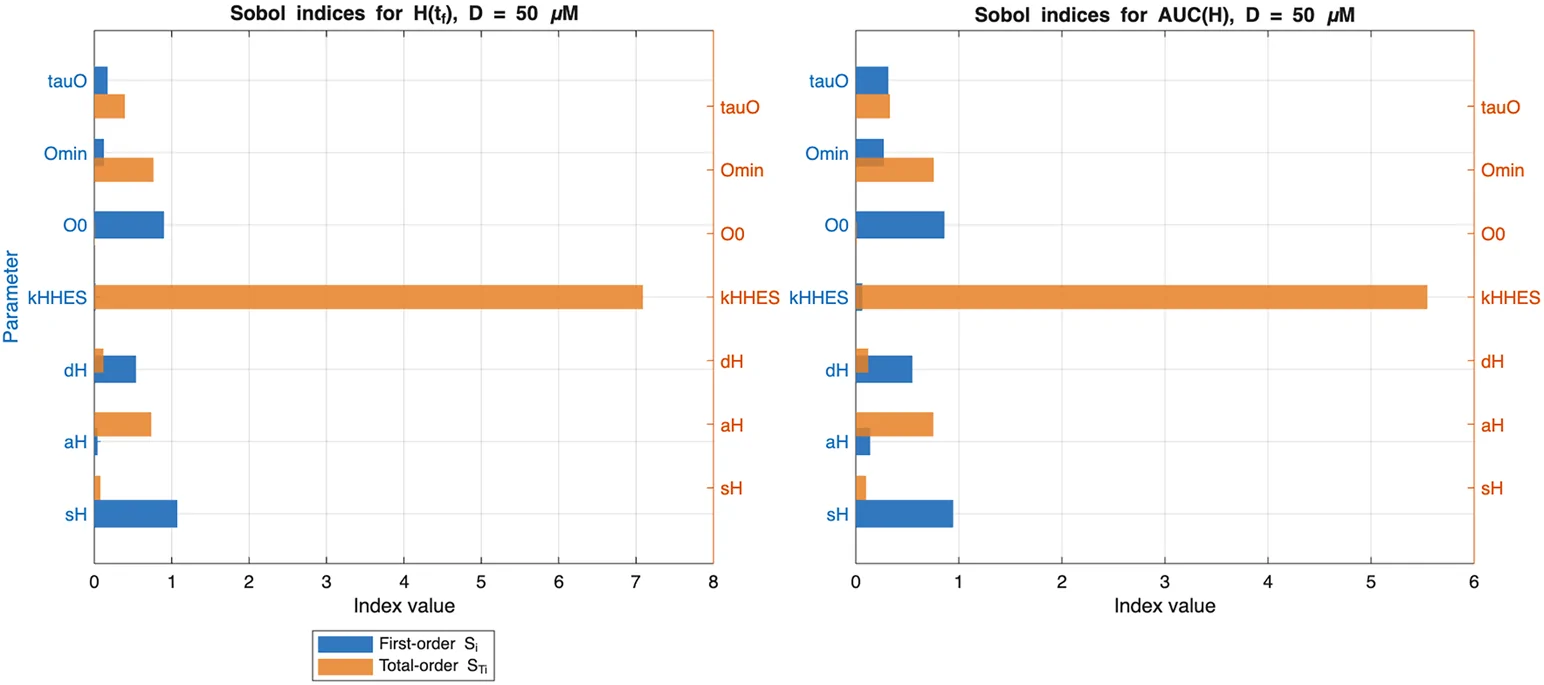
Sobol’ (Saltelli) global sensitivity analysis of the HIF−1α regulation model under D=50 µM hesperidin exposure. (a) First-order ($S_{i}$) and total-order ($S_{Ti}$) sensitivity indices for the final HIF−1α concentration $H(t_{f})$ and (b) corresponding indices for the cumulative hypoxic signal AUC(H). Blue bars indicate first-order contributions, whereas orange bars show total (including interaction) effects.

Simulation of vascular dynamics (figure 19) using equation (2.7) showed a clear dose-dependent reduction of angiogenesis under hesperidin. In the control condition (D=0 µM), V(t) expanded owing to sustained HIF−1α-driven signalling. Increasing hesperidin concentrations progressively suppressed V(t), with partial stabilization at 25–50 µM and near-complete inhibition at 100 µM. The model indicates that higher hesperidin concentrations (50–100 µM) reduce vascular growth by approximately 40–70% compared with control, consistent with the expected anti-angiogenic and microenvironment-normalizing effects of flavonoid treatment. OAT analysis (figure 20) identified $a_{H}$, $a_{V}$ and hypoxia intensity $O_{\text{drop}}$ as the main positive drivers of vascular expansion, while degradation terms ($d_{H}$, $d_{V}$) and hesperidin-related inhibition ($k_{H}^{\text{HES}}$, $k_{V}^{\text{HES}}$) exerted the strongest negative effects. Sobol’ analysis (figure 21) confirmed that vascular outcomes were dominated by hesperidin-driven suppression—particularly $k_{H}^{\mathrm{HES}}$—indicating a stable and robust HIF−1α-dependent angiogenic module under drug modulation.

**Figure 19.**
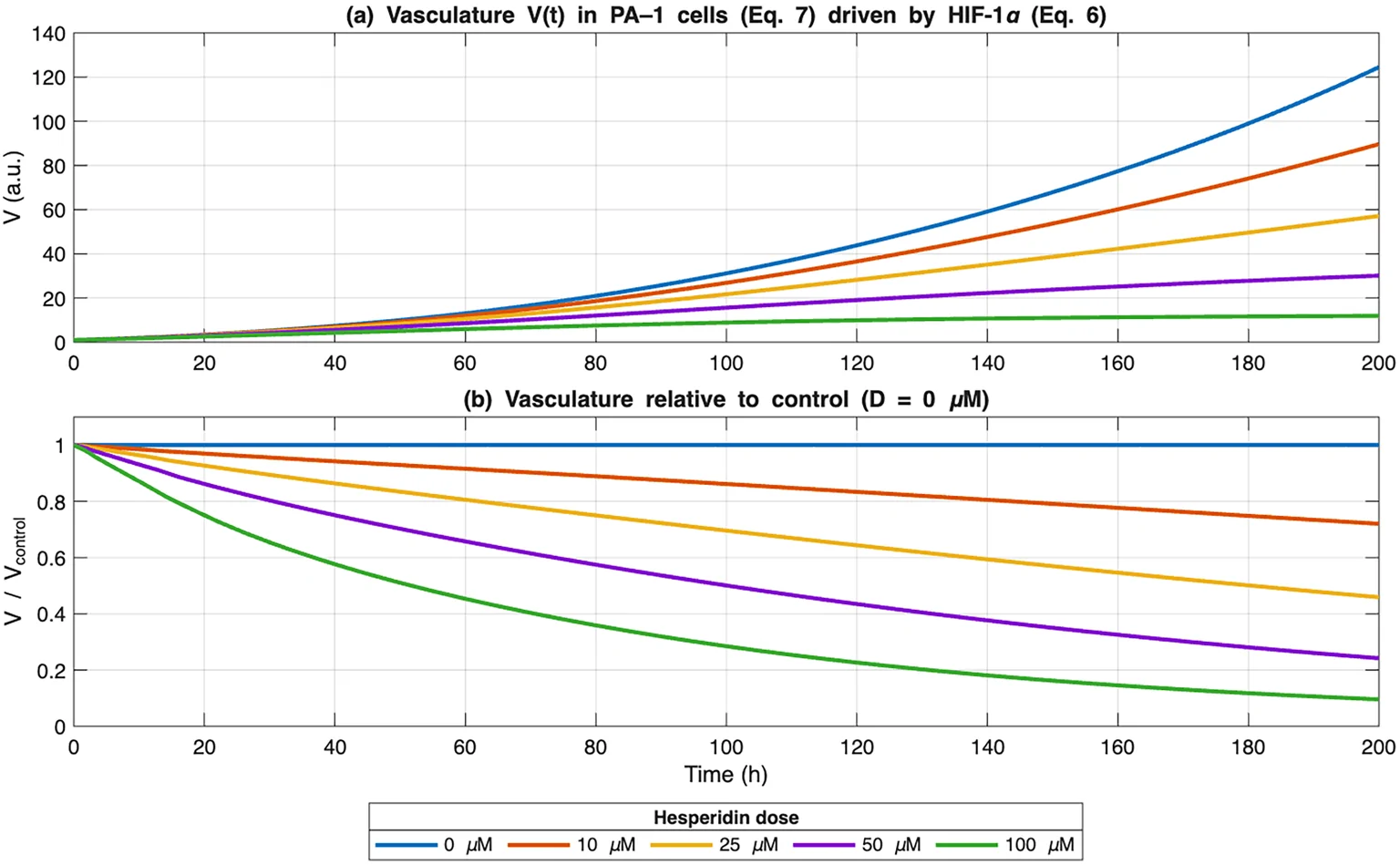
Vasculature dynamics under hesperidin modulation in the ovarian cancer microenvironment model. (a) Simulated vasculature dynamics V(t) over 200 hours at hesperidin concentrations of 0–100 µM. (b) Vasculature levels normalized to untreated control (D=0 µM).

**Figure 20.**
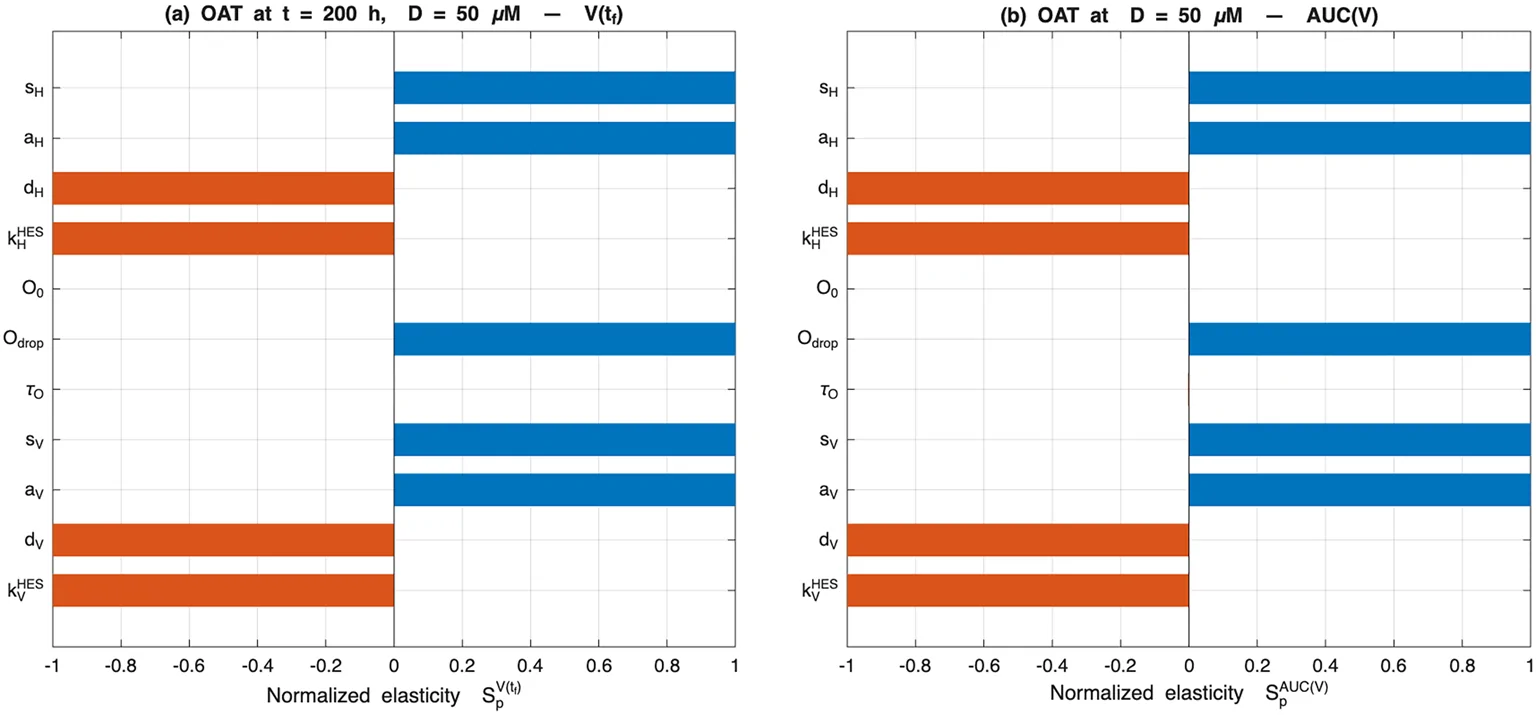
OAT sensitivity analysis for vasculature dynamics driven by HIF−1α regulation under hesperidin exposure (D=50 µM). Panels illustrate the normalized elasticities of model parameters with respect to (a) the final vasculature level ($V(t_{f}=200\;\text{h})$) and (b) the integrated angiogenic response AUC(V). Positive elasticities (blue bars) denote parameters whose increase enhances the vascular outcome, while negative elasticities (orange bars) correspond to parameters that suppress vascularization.

**Figure 21.**
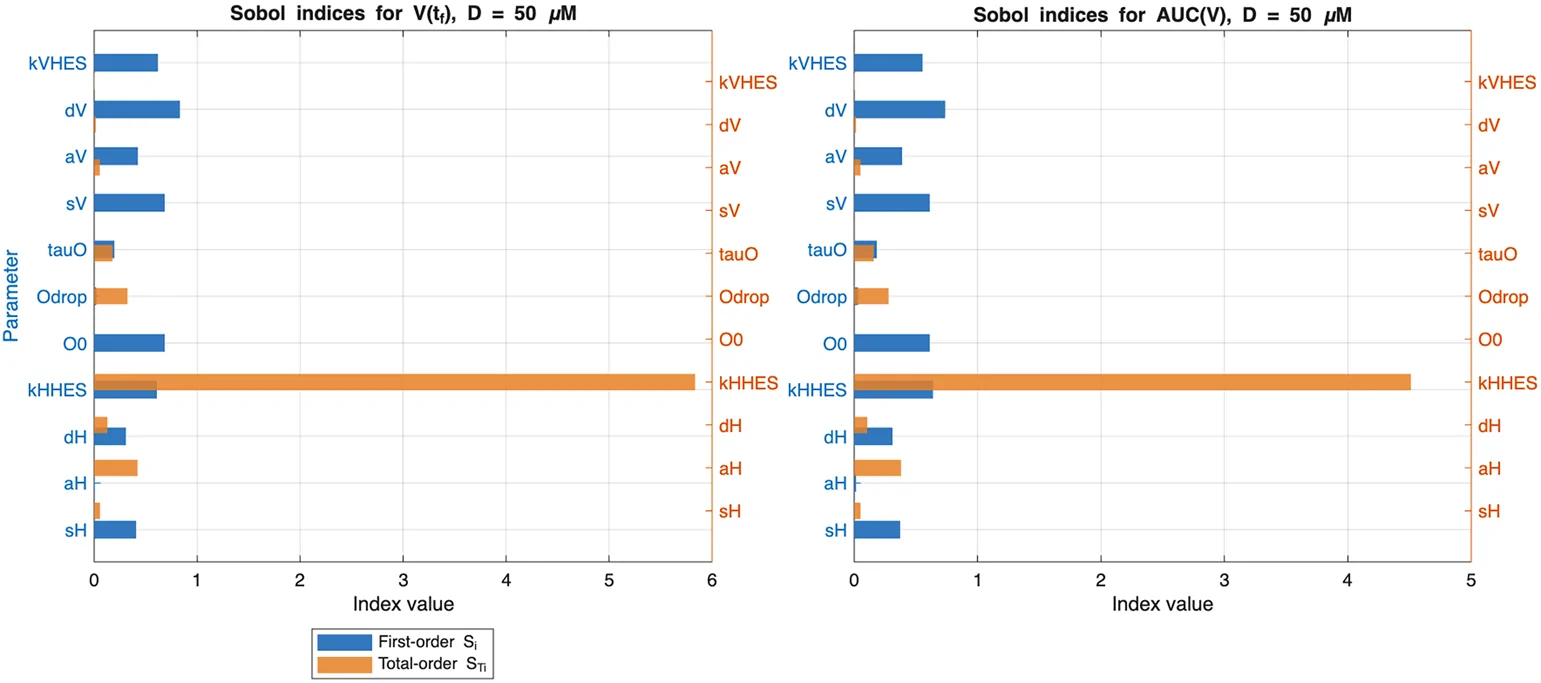
Sobol’ (Saltelli) global sensitivity analysis for vasculature dynamics driven by HIF−1α regulation under hesperidin exposure (D=50 µM). The figure shows first-order ($S_{i}$, blue) and total-order ($S_{Ti}$, orange) Sobol’ indices for (a) the final vasculature level $V(t_{f})$ = 200 h) and (b) the integrated angiogenic response AUC(V). Higher $S_{i}$ values indicate parameters exerting direct influence, while $S_{Ti}$ captures both main and interaction effects.

Simulation of CAF dynamics (figure 22) using equation (2.8) showed that hesperidin suppresses fibroblast expansion within the TME. Under control conditions, F(t) increased steadily owing to tumour-driven activation and ECM-associated stromal recruitment. Hesperidin reduced both the growth rate and steady-state levels of CAFs, with 50–100 µM producing the strongest attenuation. This effect was driven by the suppression term $k_{F}^{\mathrm{HES}}DF$, consistent with interference in TGF−β and platelet-derived-growth-factor-mediated CAF activation. Reduced CAF proliferation corresponded to diminished stromal stiffening and paracrine signalling. The model captures the indirect anti-stromal effect of hesperidin through inhibition of tumour-mediated fibroblast activation and matrix remodelling, reflecting its putative role in reducing desmoplastic and pro-tumour stromal signalling within the TME. OAT analysis (figure 23) showed that CAF behaviour was mainly controlled by $r_{T}$, $a_{F}$, $d_{F}$ and $k_{F}^{\mathrm{HES}}$, indicating stable and interpretable dynamics. Sobol’ analysis (figure 24) further confirmed that growth-related parameters ($r_{T}$, $K_{T}$, $s_{F}$, $a_{F}$) dominate global sensitivity, while $k_{F}^{\mathrm{HES}}$ consistently moderates fibroblast expansion, supporting robustness of the CAF–tumour subsystem.

**Figure 22.**
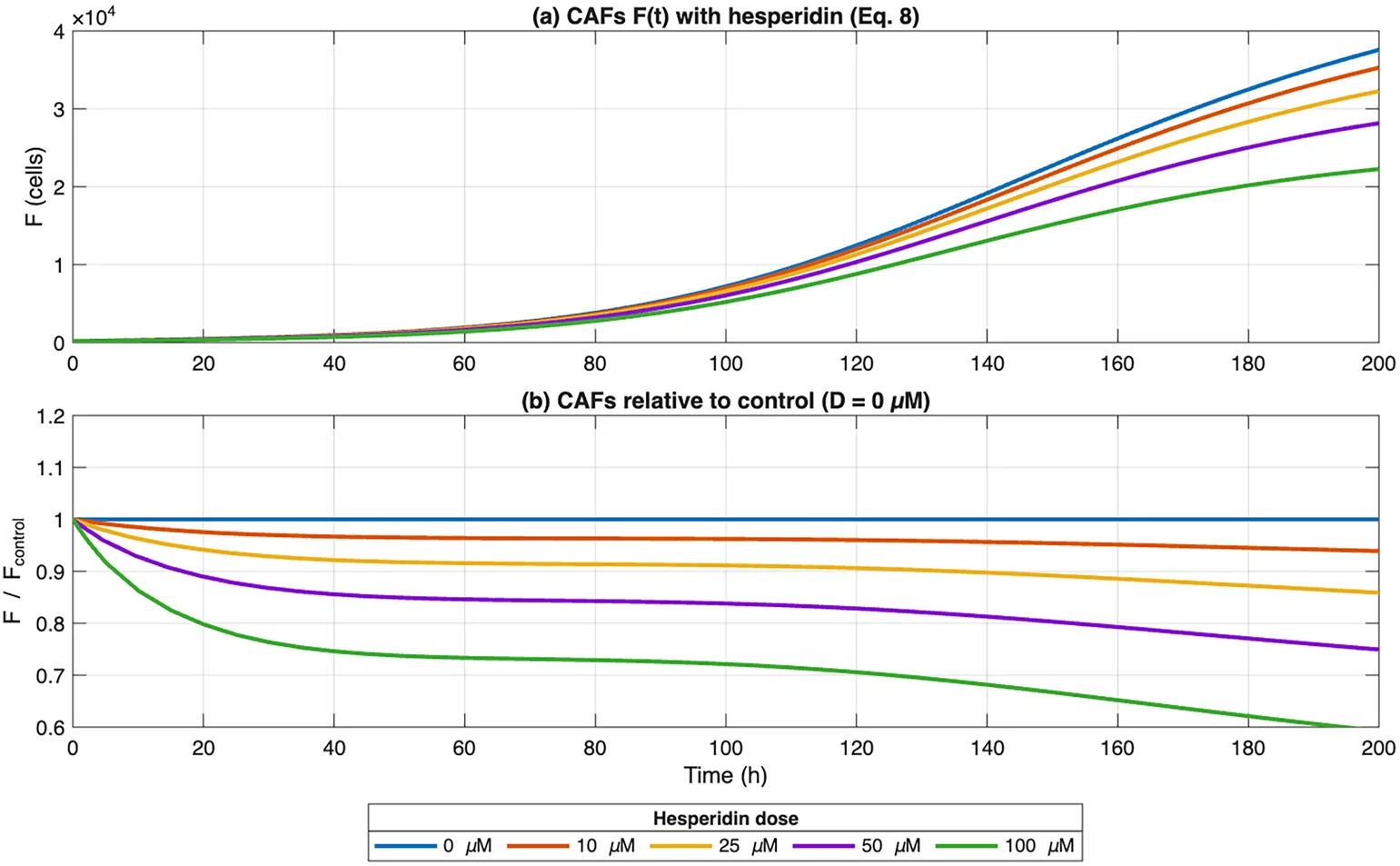
CAF dynamics under hesperidin modulation in the ovarian cancer microenvironment model. (a) Simulated CAF populations F(t) over 200 hours at hesperidin concentrations of 0–100 µM. (b) CAF levels normalized to untreated control (D=0 µM).

**Figure 23.**
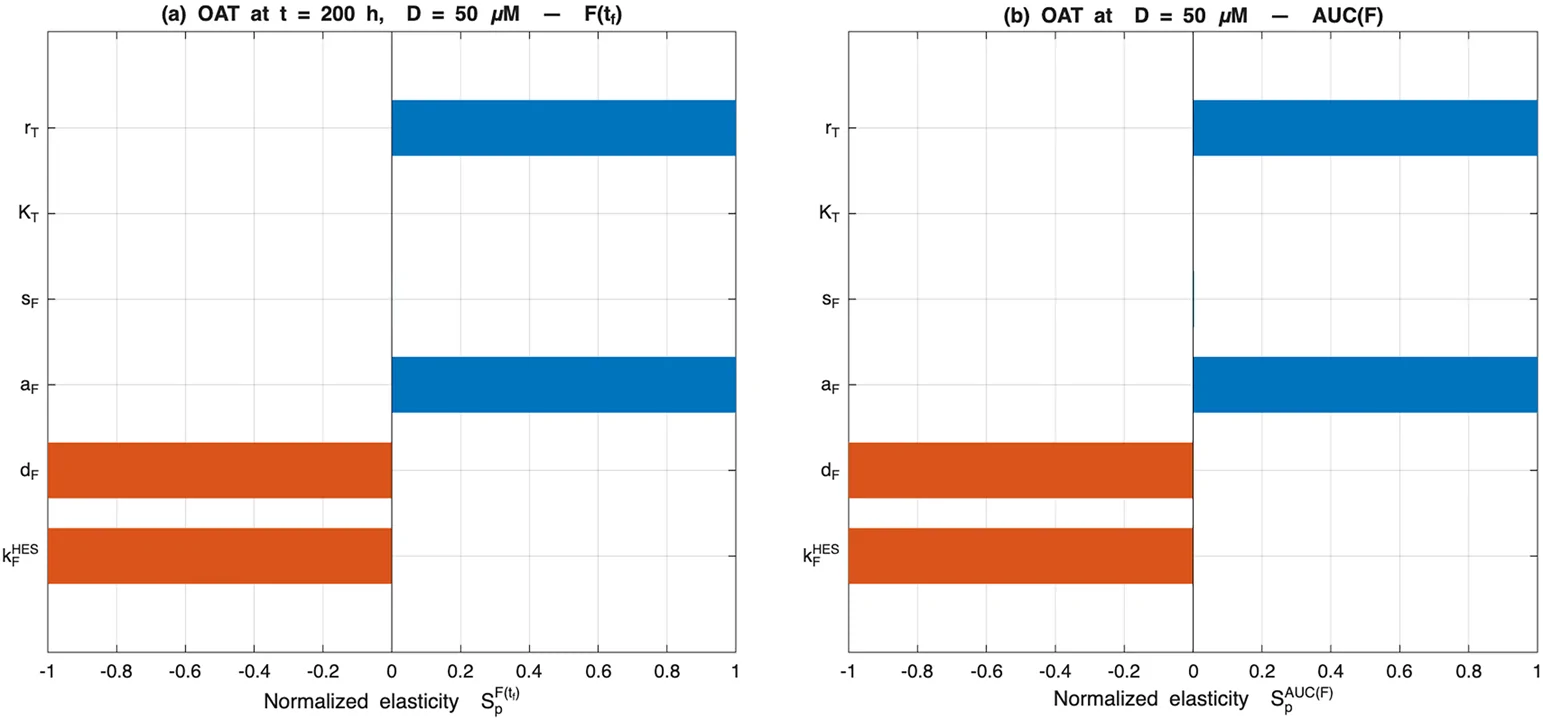
OAT sensitivity analysis for CAF dynamics coupled to tumour growth and hesperidin exposure (D=50 µM). Panels show normalized elasticities of model parameters with respect to (a) the final fibroblast population $F(t_{f}=200\;\text{h})$ and (b) the cumulative fibroblast activity AUC(F). Blue bars represent parameters whose increase promotes fibroblast expansion, whereas orange bars indicate inhibitory or suppressive effects.

**Figure 24.**
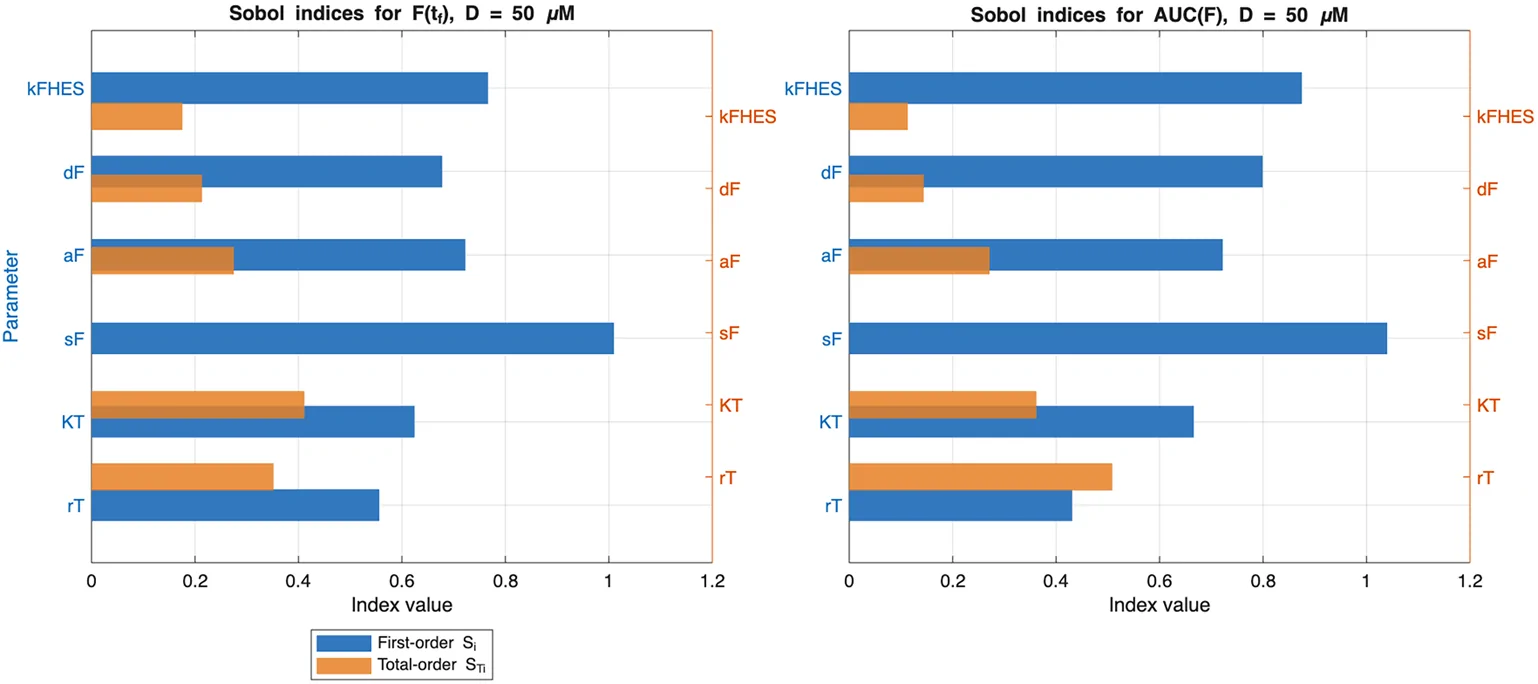
Sobol’ (Saltelli) global sensitivity analysis for CAF dynamics coupled to tumour logistic growth under hesperidin exposure (D=50 µM). The figure presents first-order ($S_{i}$, blue) and total-order ($S_{Ti}$, orange) Sobol’ indices for (a) the final fibroblast population $F(t_{f}=200\;\text{h})$ and (b) the integrated fibroblast activity AUC(F). High $S_{i}$ values indicate parameters that directly drive CAF variability, whereas $S_{Ti}$ reflects both main and interaction effects.

Simulation of ECM stiffness dynamics (equation (2.9)) showed a clear link between CAF activity and matrix remodelling (figure 25). In the control condition (D=0 µM), stiffness S(t) increased continuously owing to CAF-driven deposition and the positive feedback term $\beta_{2}FS$. Hesperidin (50–100 µM) disrupted this feedback by suppressing CAF growth (equation (2.8)), resulting in reduced ECM accumulation and lower steady-state stiffness. This reflects downstream normalization of mechanical properties through inhibition of CAF activation. The results indicate that hesperidin may reduce CAF-mediated ECM reinforcement and mechanical feedback within the TME, consistent with a mechano-modulatory effect on stromal remodelling. OAT analysis (figure 26) showed dominant sensitivity to deposition parameters $\beta_{1}$ and $\beta_{2}$, opposed by degradation and hesperidin-related inhibitory terms, confirming robust coupling between CAF dynamics and matrix mechanics. Sobol’ analysis (figure 27) indicated a stable, hierarchical pattern dominated by the CAF activation parameter $a_{F}$, with hesperidin suppression ($k_{F}^{\mathrm{HES}}$) acting as a secondary stabilizer limiting excessive matrix build-up.

**Figure 25.**
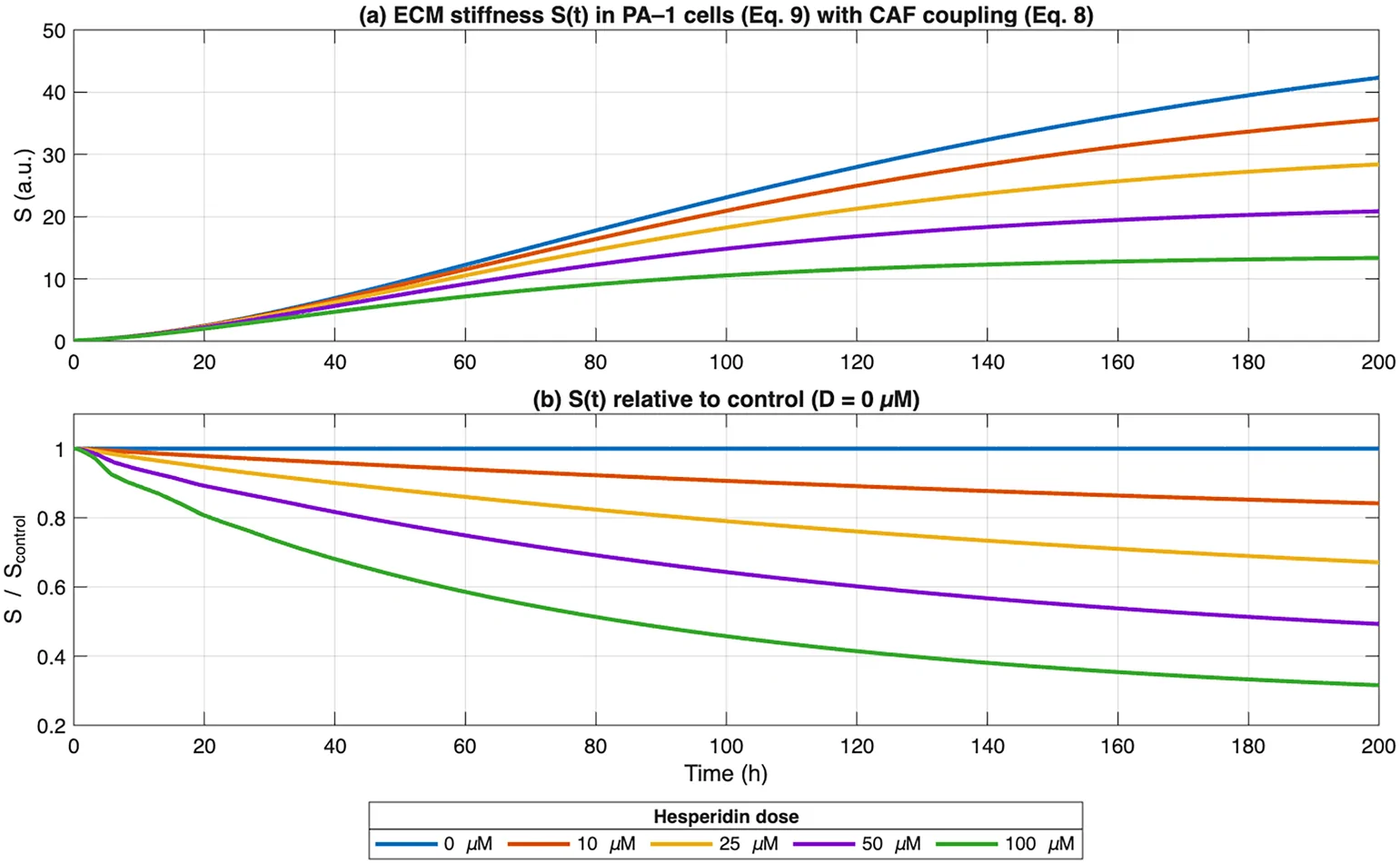
ECM stiffness dynamics under hesperidin modulation in the ovarian cancer microenvironment model. (a) Simulated ECM stiffness S(t) over 200 hours at hesperidin concentrations of 0–100 µM. (b) ECM stiffness levels normalized to untreated control (D=0 µM).

**Figure 26.**
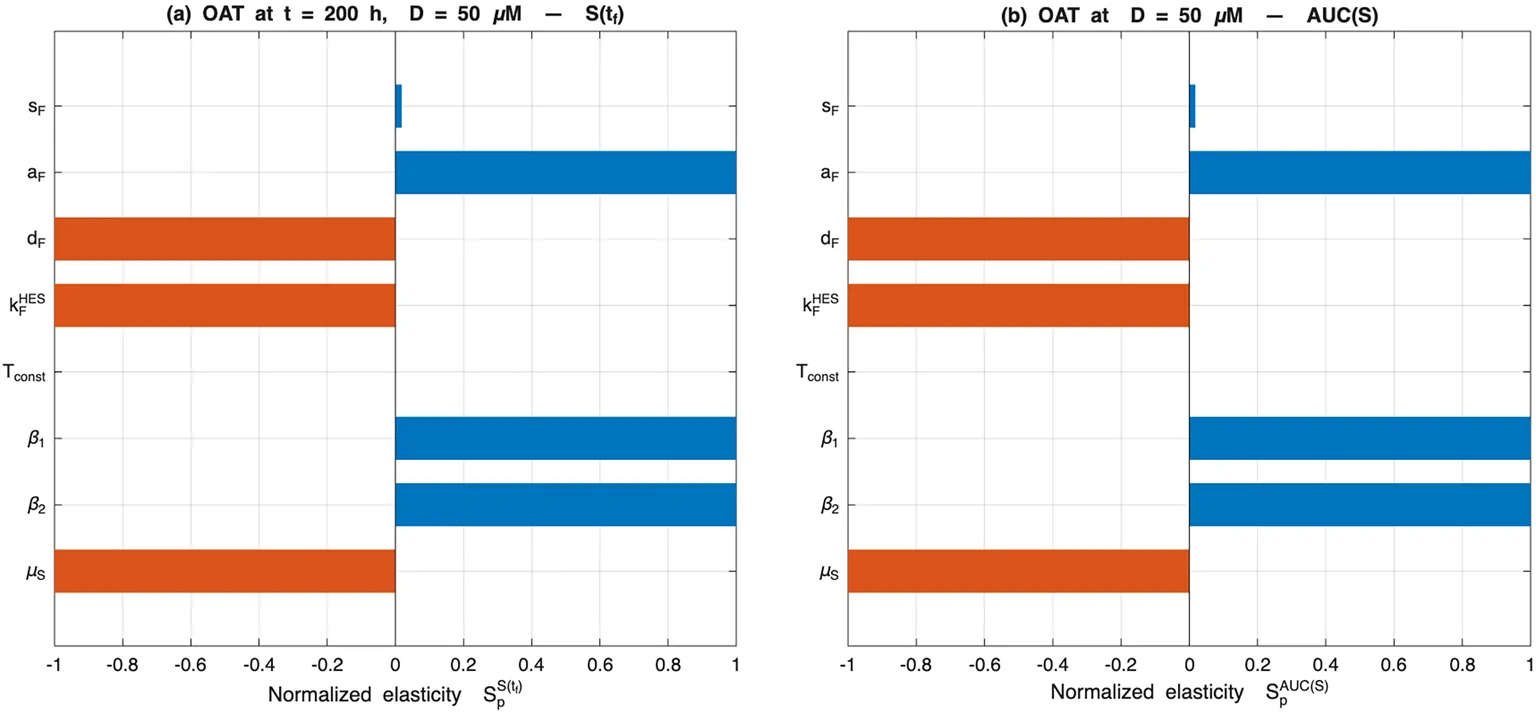
OAT local sensitivity analysis of ECM stiffness S(t) coupled to CAF dynamics under hesperidin exposure (D=50 µM). Panels display normalized elasticities ($S_{p}^{S(t_{f})}$ and $S_{p}^{\text{AUC}(S)}$) for (a) final ECM stiffness at $t_{f}=200\;\text{h}$ and (b) cumulative stiffness response. Positive (blue) bars denote parameters whose increase augments ECM stiffness, whereas negative (orange) bars indicate parameters that reduce it.

**Figure 27.**
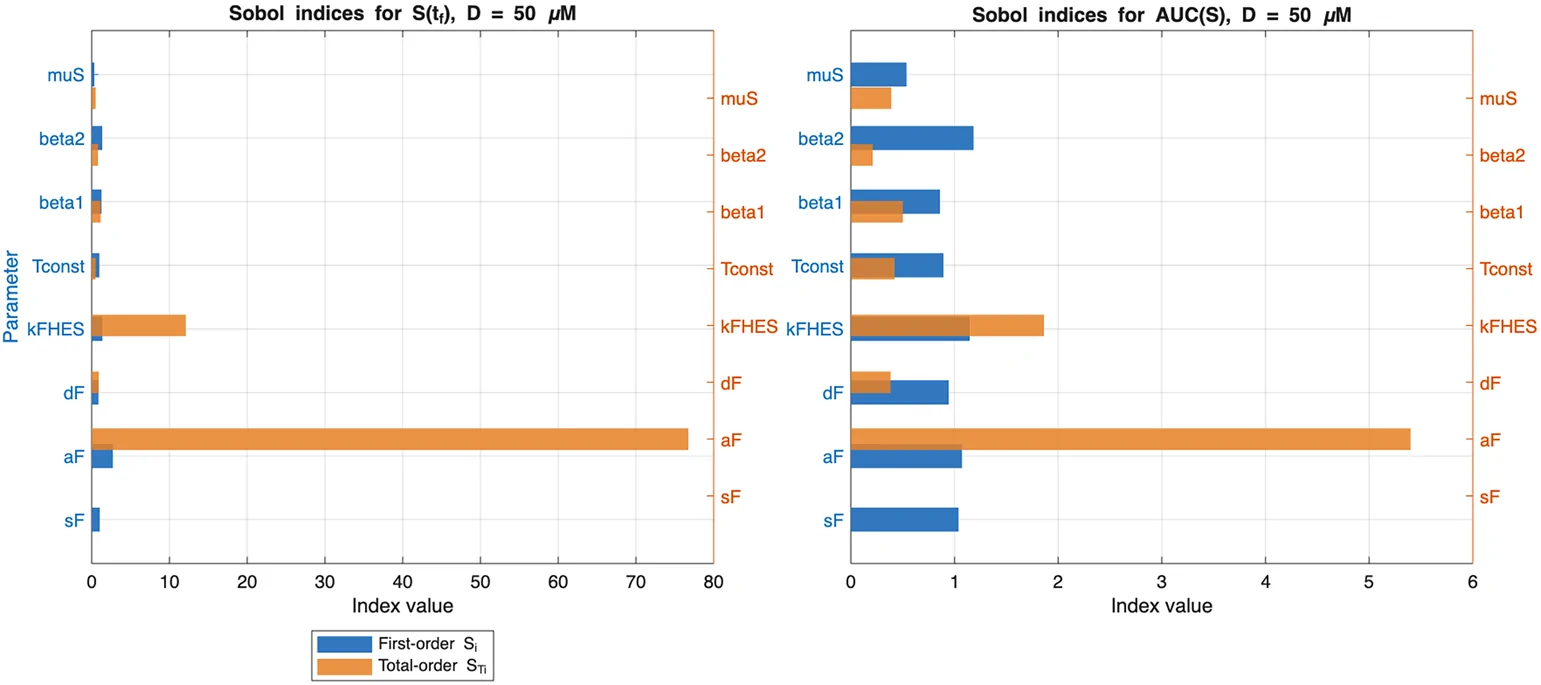
Sobol’ (Saltelli) global sensitivity analysis for ECM stiffness S(t) coupled to CAF dynamics under hesperidin exposure (D=50 µM). The plots display first-order ($S_{i}$, blue) and total-order ($S_{Ti}$, orange) Sobol’ indices for (a) the final ECM stiffness $S(t_{f}=200\;\text{h}$) and (b) the cumulative stiffness response AUC(S). Parameters with high $S_{i}$ indicate dominant direct influence, while $S_{Ti}$ captures the combined main and interaction effects.

Simulation of MMP activity (figure 28) showed that hesperidin reduces ECM remodelling in a dose-dependent manner. In the control condition, M(t) increased steadily owing to continuous production by tumour cells and CAFs. Hesperidin (50–100 µM) slowed this accumulation, leading to lower steady-state MMP levels, primarily through reduced CAF proliferation (equation (2.8)) and diminished tumour-derived proteolytic signalling. The normalized curves $M/M_{\mathrm{control}}$ declined to 0.6–0.8 at the highest doses, indicating indirect suppression of matrix degradation via microenvironmental modulation rather than direct enzymatic inhibition. These results suggest that hesperidin contributes to reduced ECM degradation and mechanical softening of the TME. OAT analysis (figure 29) showed that tumour proliferation and CAF activation were the dominant positive drivers of MMP activity, while decay terms ($d_{F}$, $\delta_{M}$) and hesperidin suppression ($k_{F}^{\mathrm{HES}}$) stabilized the system. Sobol’ analysis (figure 30) confirmed that production parameters ($\gamma_{F}$, $\gamma_{T}$, $s_{F}$, $a_{F}$) and clearance ($\delta_{M}$) dominate global sensitivity, with $k_{F}^{\mathrm{HES}}$ and $d_{F}$ providing robust stabilizing effects against excessive proteolysis.

**Figure 28.**
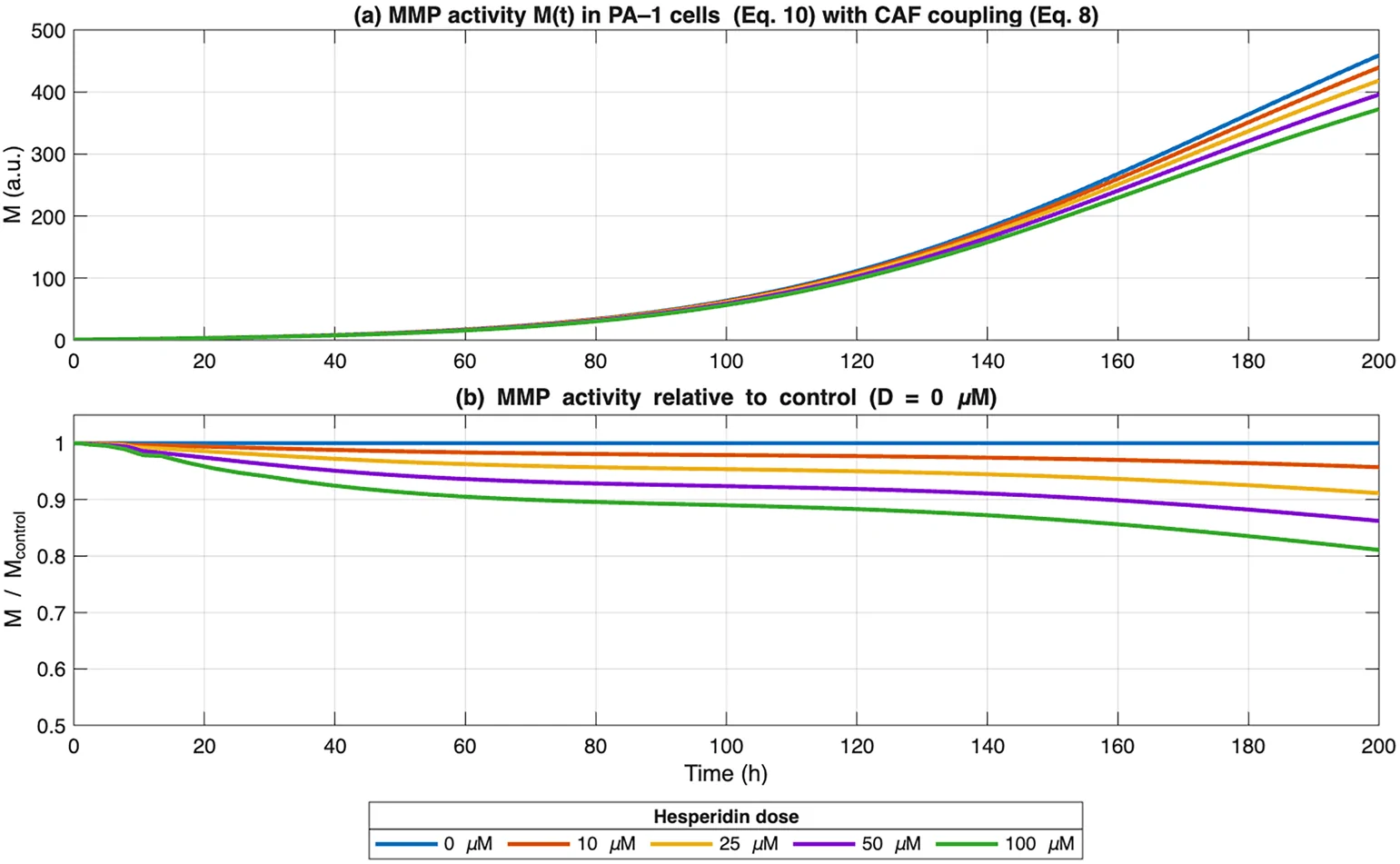
MMP activity dynamics under hesperidin modulation in the ovarian cancer microenvironment model. (a) Simulated MMP activity M(t) over 200 hours at hesperidin concentrations of 0–100 µM. (b) MMP activity levels normalized to untreated control (D=0 µM).

**Figure 29.**
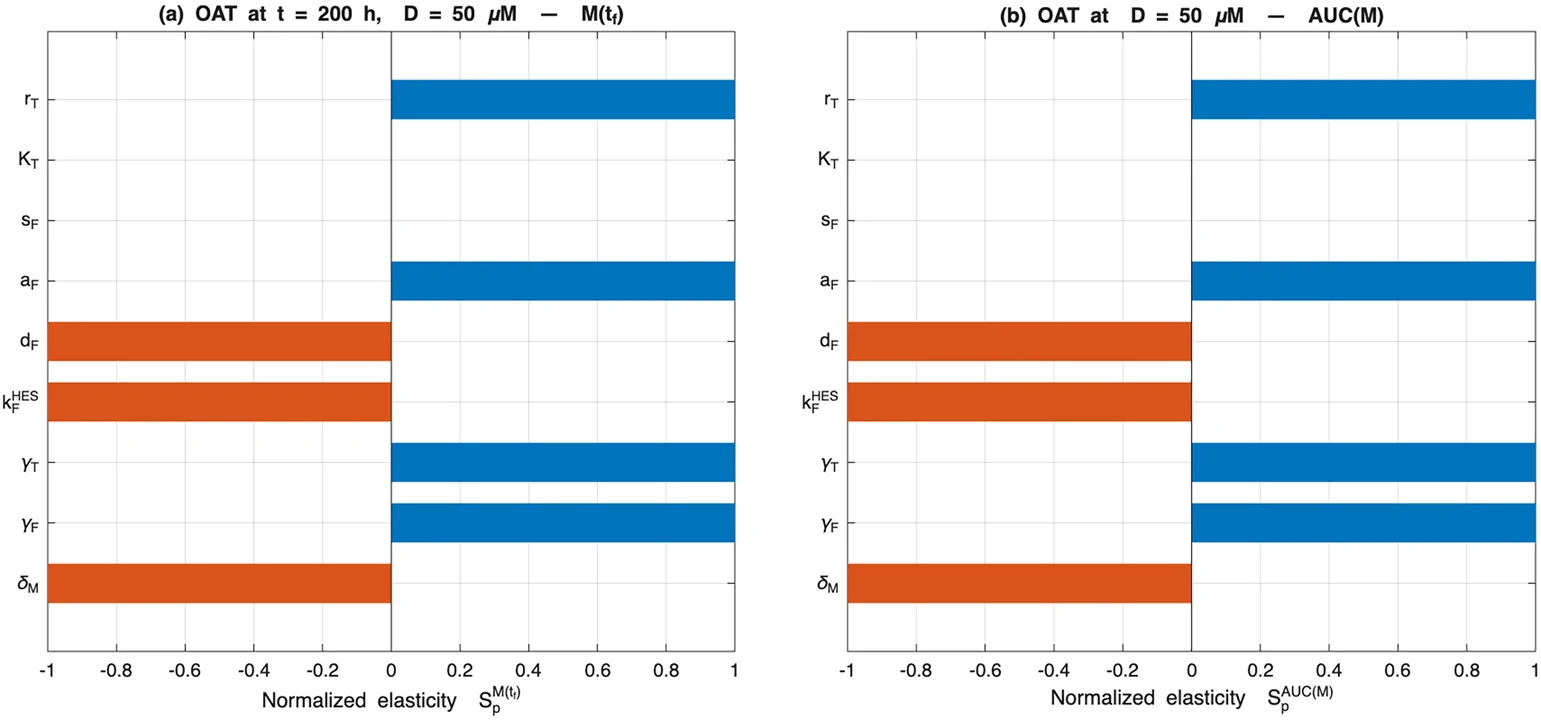
OAT sensitivity analysis for MMP activity M(t) coupled to CAF and tumour dynamics under hesperidin exposure (D=50 µM). The normalized elasticities $S_{p}^{M(t_{f})}$ (a) and $S_{p}^{\text{AUC}(M)}$ (b) quantify the relative influence of each parameter on the final MMP level and its cumulative activity. Blue bars indicate parameters whose increase amplifies MMP output, while orange bars correspond to inhibitory effects.

**Figure 30.**
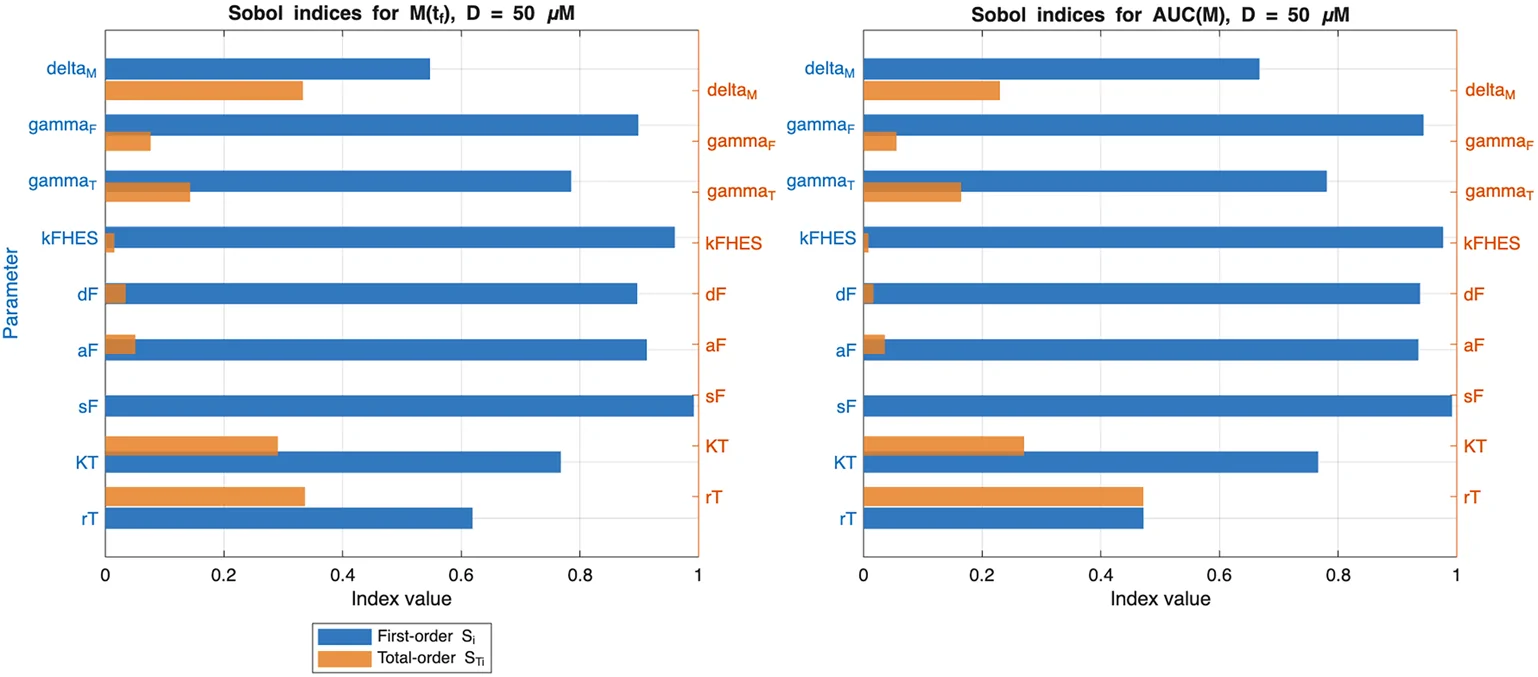
Sobol’ (Saltelli) global sensitivity analysis for MMP activity M(t) under hesperidin (D=50 µM), with tumour–CAF coupling and logistic tumour growth. Panels report first-order ($S_{i}$, blue) and total-order ($S_{Ti}$, orange) indices for (a) the terminal level $M(t_{f}=200\;h)$ and (b) the cumulative response AUC(M). Parameters probed were tumour kinetics ($r_{T}$, $K_{T}$), CAF fluxes ($s_{F}$, $a_{F}$, $d_{F}$), hesperidin sensitivity ($k_{F}^{\mathrm{HES}}$), MMP production rates from tumour and CAFs ($\gamma_{T}$, $\gamma_{F}$) and MMP clearance ($\delta_{M}$).

Simulation of glucose concentration G(t) (figure 31) showed an overall rise to steady state under all conditions. In the control group, glucose declined owing to continuous consumption by tumour cells, effector T cells and CAFs. Increasing hesperidin doses (10–100 µM) reduced glucose use, resulting in higher G(t) and elevated normalized levels $G/G_{\mathrm{control}}$ after 200 hours. This reflects decreased metabolic demand and altered nutrient distribution in the TME under hesperidin exposure. OAT analysis (figure 32) indicated that glucose dynamics was mainly driven by metabolic flux parameters ($s_{G}$, $k_{u}$, $k_{c}$, $d_{G}$), while hesperidin-associated terms ($\theta_{TG}$, $\theta_{EG}$) acted as stabilizing feedback limiting excessive depletion. Sobol’ analysis (figure 33) confirmed strong structural stability, with sensitivity concentrated in metabolic turnover parameters and minimal contributions from immune or hesperidin-related terms, indicating a robust metabolic subsystem.

**Figure 31.**
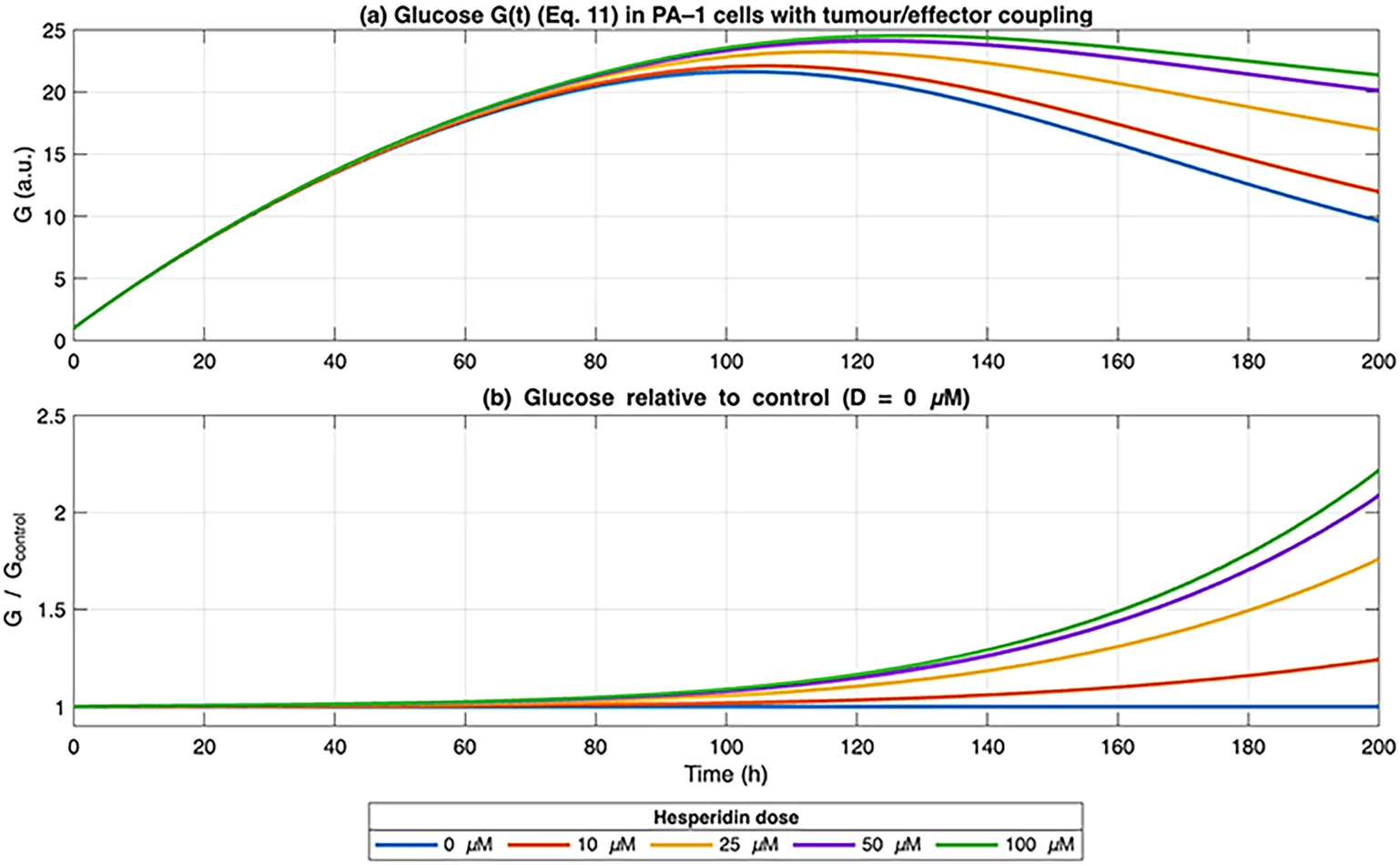
Glucose dynamics under hesperidin modulation in the ovarian cancer microenvironment model. (a) Simulated glucose levels G(t) over 200 hours at hesperidin concentrations of 0–100 µM. (b) Glucose levels normalized to untreated control (D=0 µM).

**Figure 32.**
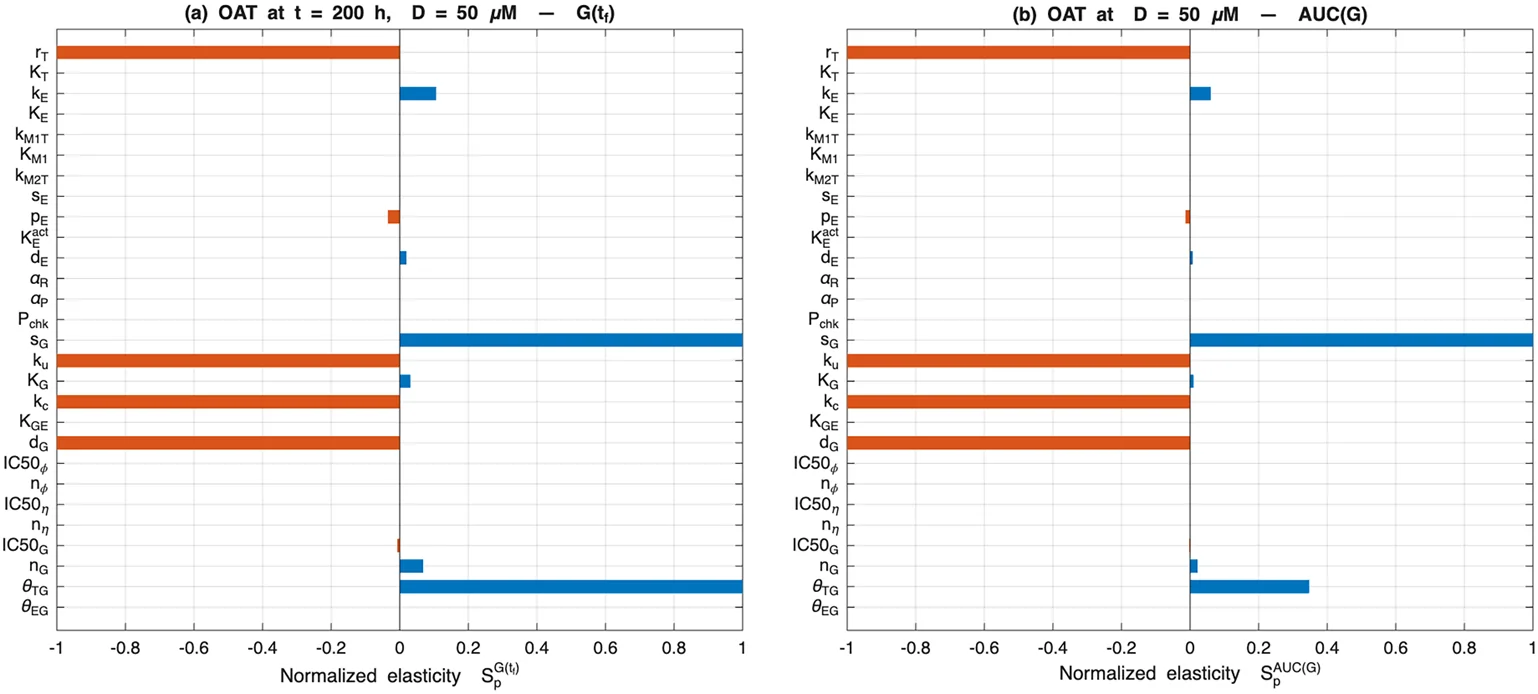
OAT sensitivity analysis for glucose dynamics G(t) in the tumour–effector–metabolic module under hesperidin (D=50 µM). Panels show normalized elasticities (blue = positive and orange = negative) for (a) terminal glucose $G(t_{f}=200\;h)$ and (b) cumulative exposure AUC(G). Parameters scanned included tumour kinetics ($r_{T},K_{T},k_{E},K_{E}$), effector cell turnover ($s_{E},p_{E},d_{E},K_{E}^{\text{act}}$), glucose flux constants ($s_{G},k_{u},k_{c},K_{G},K_{GE},d_{G}$) and hesperidin Hill terms ($IC50_{\phi },n_{\phi },IC50_{\eta },n_{\eta },IC50_{G},n_{G}$) with uptake-tempering coefficients ($\theta_{TG},\theta_{EG}$).

**Figure 33.**
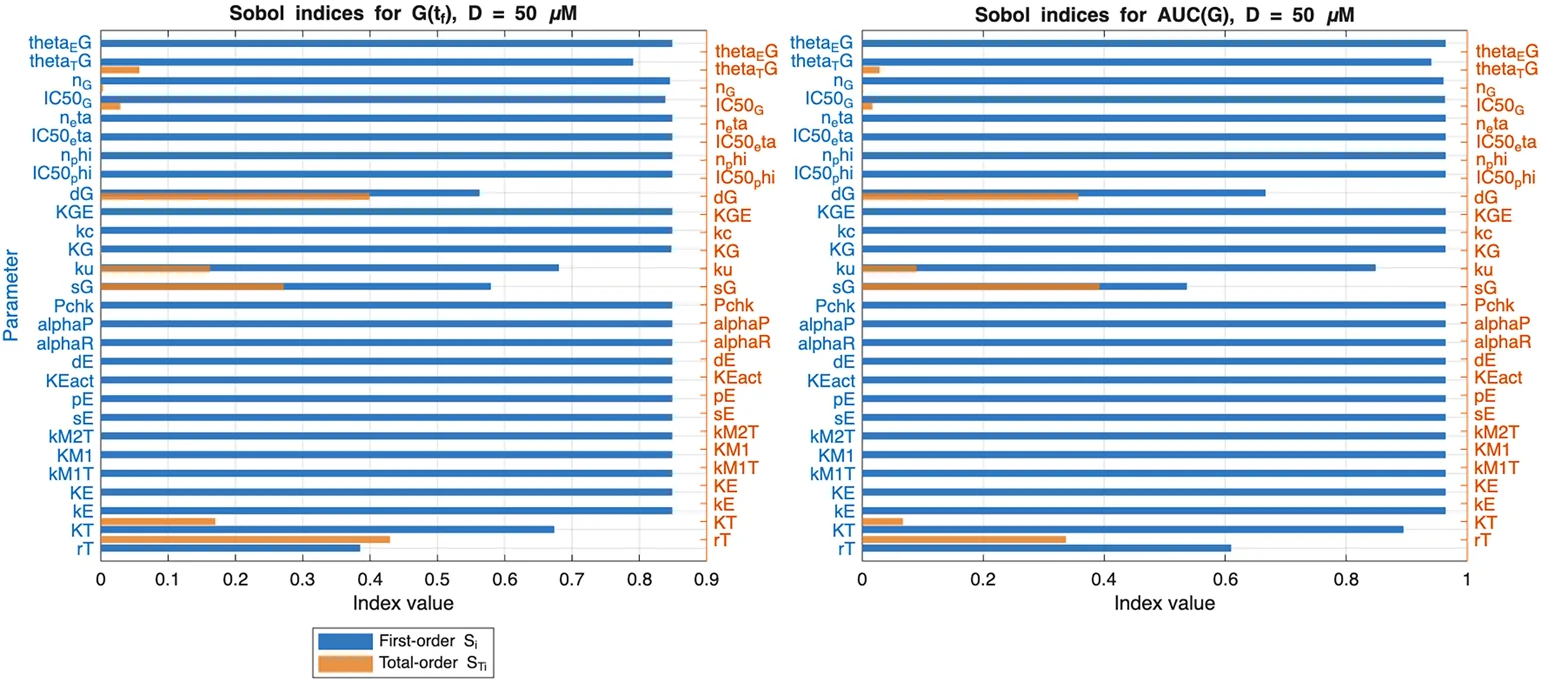
Sobol’ (Saltelli) global sensitivity analysis for glucose dynamics G(t) in the tumour–effector–metabolic subsystem under hesperidin (D=50 µM). Panels show first-order ($S_{i}$, blue) and total-order ($S_{Ti}$, orange) Sobol’ indices for (a) terminal glucose concentration $G(t_{f}=200\;h)$ and (b) cumulative glucose exposure AUC(G). The analysis covers tumour growth and killing parameters ($r_{T},K_{T},k_{E},K_{E}$), effector cell kinetics ($s_{E},p_{E},d_{E},K_{E}^{act}$), glucose flux constants ($s_{G},k_{u},k_{c},K_{G},K_{GE},d_{G}$) and hesperidin modulation terms ($IC50_{\phi },n_{\phi },IC50_{\eta },n_{\eta },IC50_{G},n_{G},\theta_{TG},\theta_{EG}$).

Simulation of lactate dynamics (figure 34) showed a strong dose-dependent reduction of lactate accumulation under hesperidin. In the control condition (D=0µM), L(t) increased steadily owing to continuous tumour- and CAF-driven production. Hesperidin reduced both the accumulation rate and final lactate levels, with 50–100 µM producing the largest decrease. This effect arose from inhibited CAF-derived lactate production and enhanced clearance, yielding a two- to threefold reduction in $L/L_{\mathrm{control}}$. These results highlight the potential ability of hesperidin to counteract tumour-driven glycolytic acidification and stromal lactate feedback within the simulated microenvironment. OAT analysis (figure 35) indicated that lactate behaviour was mainly governed by $r_{T}$, $d_{F}$, $k_{uT}$, $d_{G}$, $d_{L}$ and $k_{L}^{\mathrm{HES}}$, with other parameters contributing minimally. Sobol’ analysis (figure 36) confirmed stable system structure, with dominant sensitivity to lactate clearance and hesperidin-driven removal ($d_{L}$, $k_{L}^{\mathrm{HES}}$), and notable interaction-driven effects from CAF- and glucose-related terms along the coupled metabolic axis.

**Figure 34.**
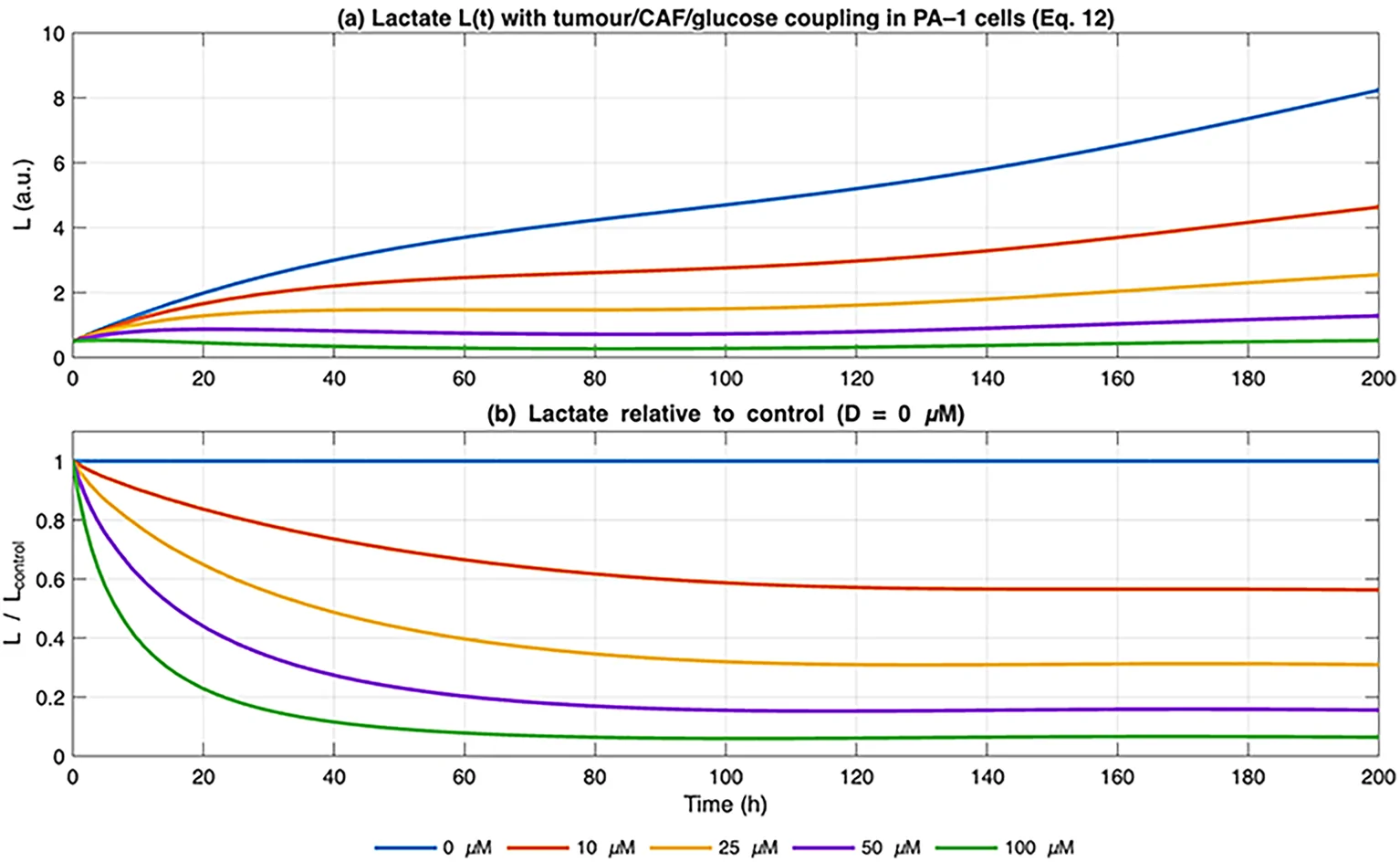
Lactate dynamics under hesperidin modulation in the ovarian cancer microenvironment model. (a) Simulated lactate levels L(t) over 200 hours at hesperidin concentrations of 0–100 µM. (b) Lactate levels normalized to untreated control (D=0 µM).

**Figure 35.**
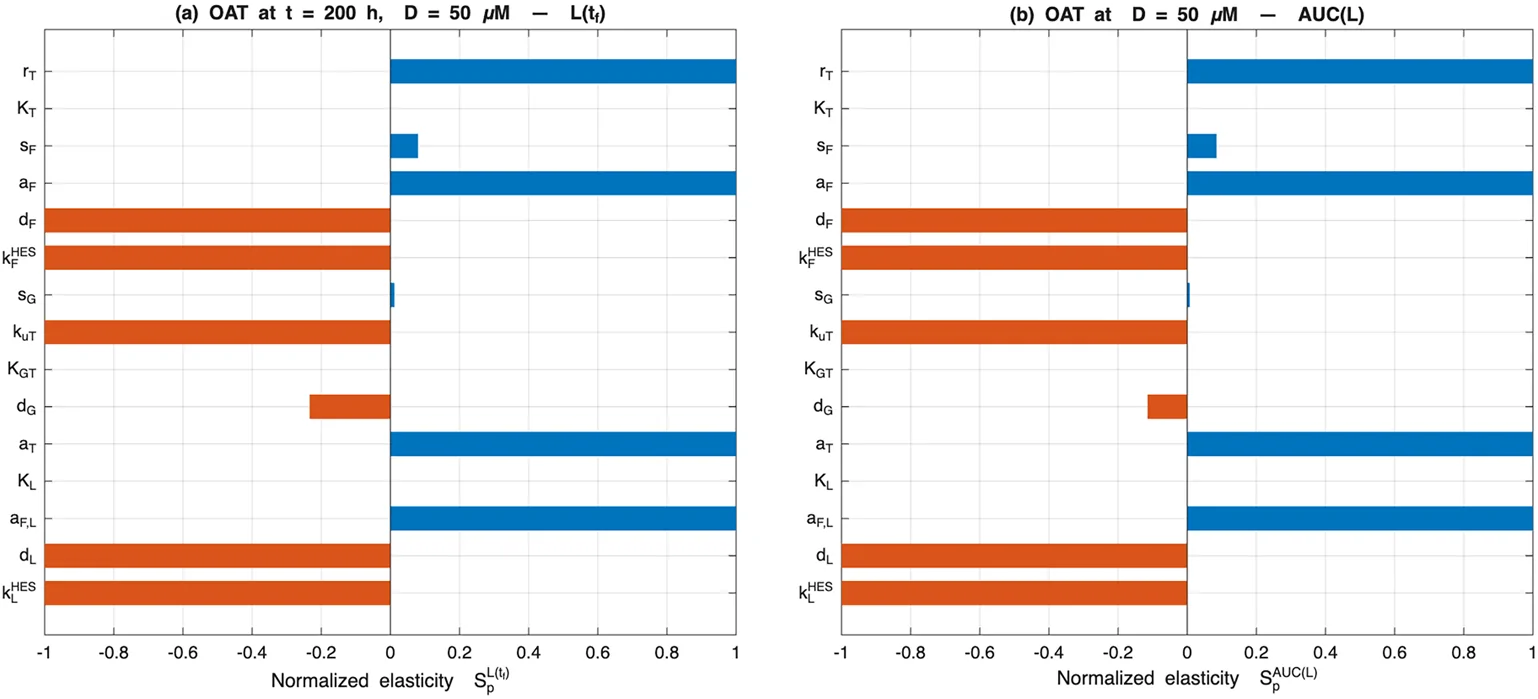
OAT sensitivity analysis of lactate dynamics under hesperidin treatment (D=50 µM). Panel (a) shows the normalized elasticities $S_{p}^{L(t_{f})}$ for the terminal lactate concentration $L(t_{f})$; panel (b) presents the elasticities $S_{p}^{\mathrm{AUC}(L)}$ for the time-integrated lactate burden. Positive bars (blue) indicate that increasing a parameter increases the output, whereas negative bars (orange) indicate a suppressive influence.

**Figure 36.**
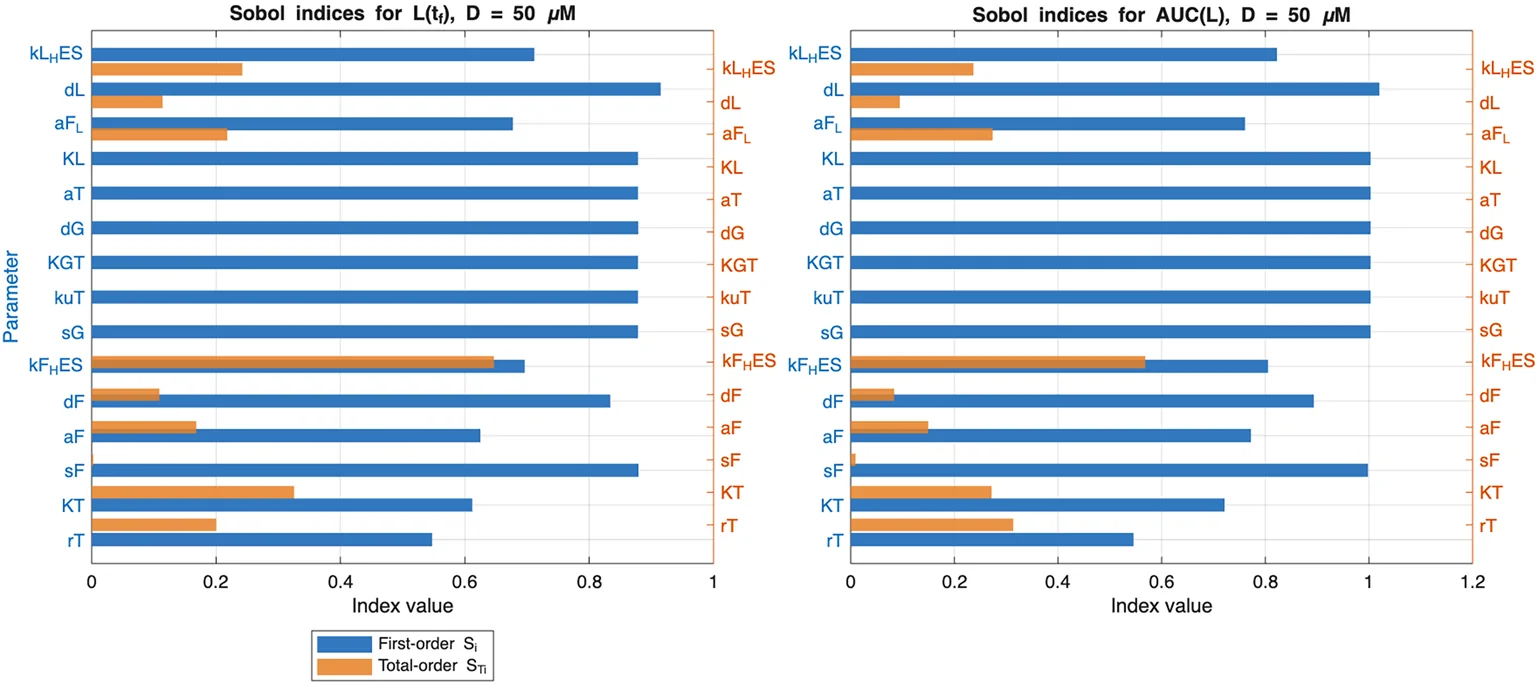
Sobol’ (Saltelli) global sensitivity analysis or lactate dynamics (equation (2.12)) under a hesperidin dose of 50 µM. (a) First-order ($S_{i}$, blue) and total-order ($S_{Ti}$, orange) Sobol’ indices for the terminal lactate concentration $L(t_{f})$ and (b) corresponding indices for the integrated lactate burden AUC(L). The analysis spans tumour growth parameters ($r_{T},K_{T}$), CAF dynamics ($s_{F},a_{F},d_{F},k_{F}^{\mathrm{HES}}$), glucose-related terms ($s_{G},k_{uT},K_{GT},d_{G}$) and lactate-specific parameters ($a_{T},K_{L},a_{F,L},d_{L},k_{L}^{\mathrm{HES}}$).

Simulation of NRF2 dynamics (figure 37) showed that untreated conditions produced a continuous rise in NRF2 activity owing to increasing ROS levels (figure 38). Hesperidin reduced NRF2 activation in a dose-dependent manner, with effects appearing from 25 µM onward, leading to lower steady-state values. This reflects hesperidin’s capacity to limit NRF2 overactivation during oxidative stress. This dynamics suggests the compound’s predicted antioxidant and chemosensitizing effects through modulation of oxidative-stress-responsive transcription. OAT analysis (figure 39) indicated that NRF2 behaviour was highly sensitive only to $d_{N}$ and $k_{N}^{\mathrm{HES}}$, while all other kinetic and ROS-related parameters had minimal influence, confirming dominance of degradation and hesperidin-driven suppression. Sobol’ analysis (figure 40) showed global sensitivity concentrated in $s_{N}$, $a^{\text{ROS}}$ and ROS-profile parameters, with $k_{N}^{\mathrm{HES}}$ producing the strongest nonlinear and interaction effects, establishing it as the primary regulatory factor for hesperidin-mediated NRF2 control.

**Figure 37.**
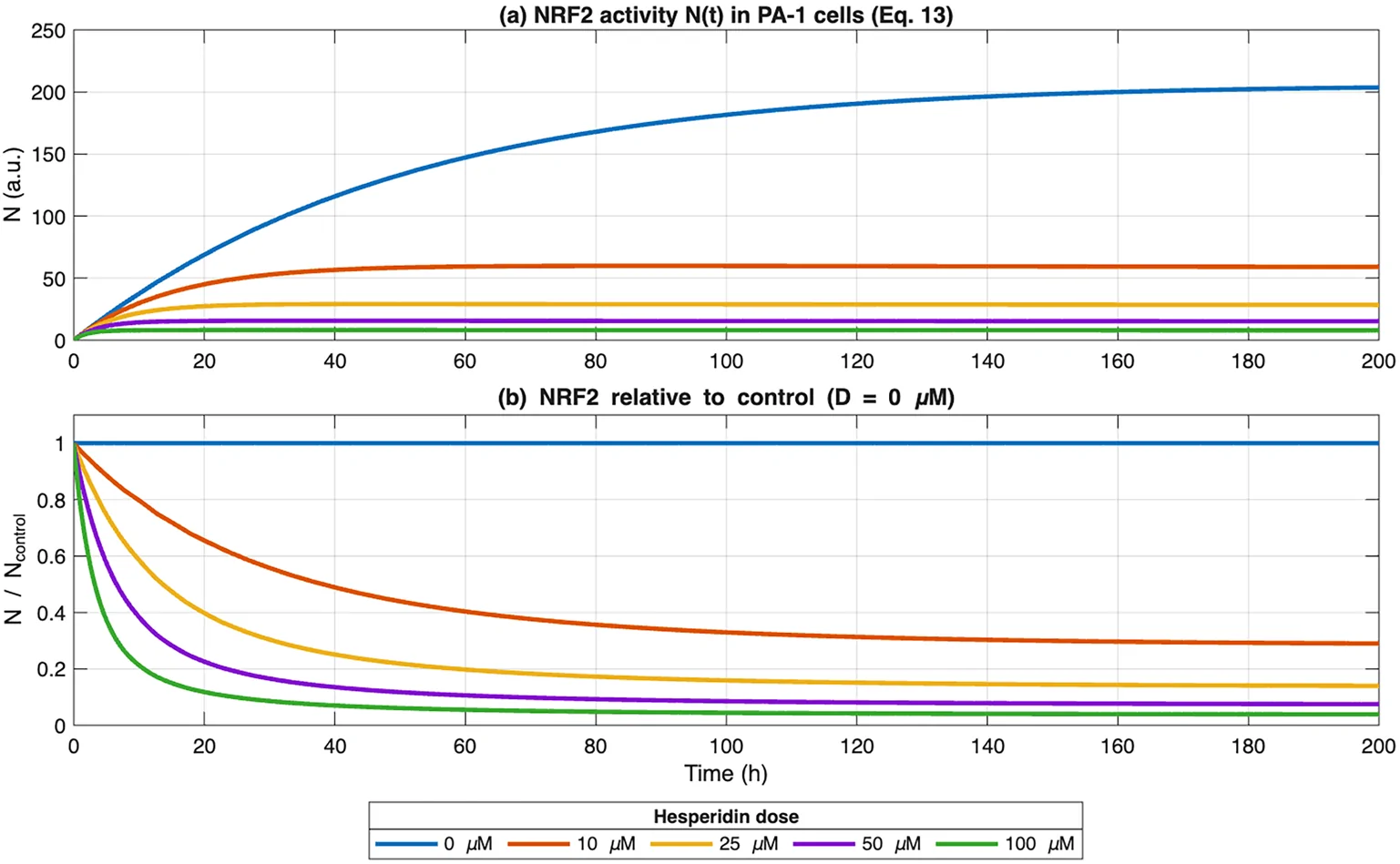
NRF2 dynamics under hesperidin modulation in the ovarian cancer microenvironment model. (a) Simulated NRF2 levels N(t) over 200 hours at hesperidin concentrations of 0–100 µM. (b) NRF2 levels normalized to untreated control (D=0 µM).

**Figure 38.**
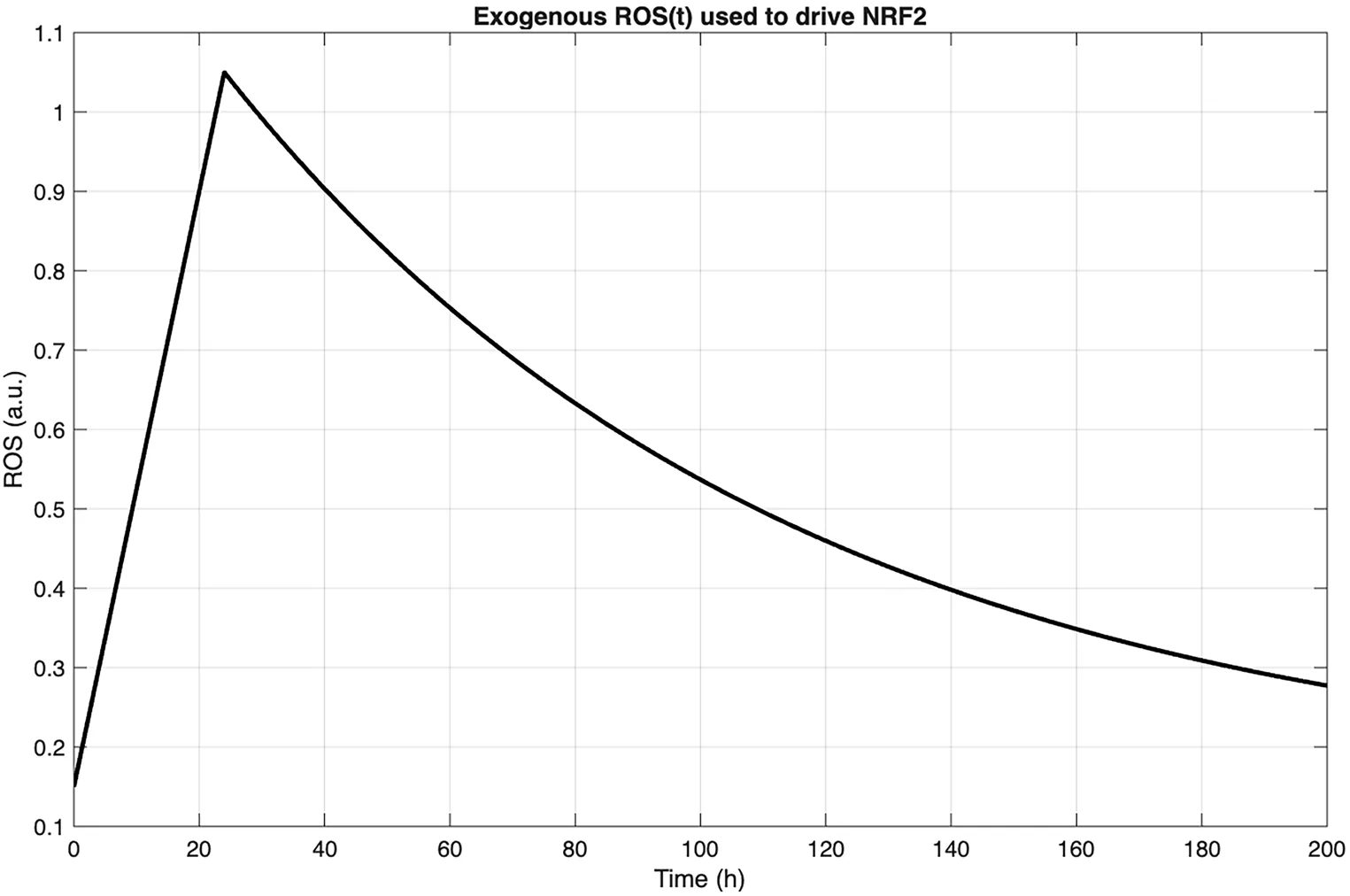
Exogenous ROS function R(t) used to drive NRF2 activation in the ODE model. The ROS profile follows a transient oxidative-stress pulse, with a rapid rise during the first 24 hours simulating hypoxia- or metabolism-induced stress, followed by a gradual decay representing antioxidant recovery and redox homoeostasis restoration within the TME.

**Figure 39.**
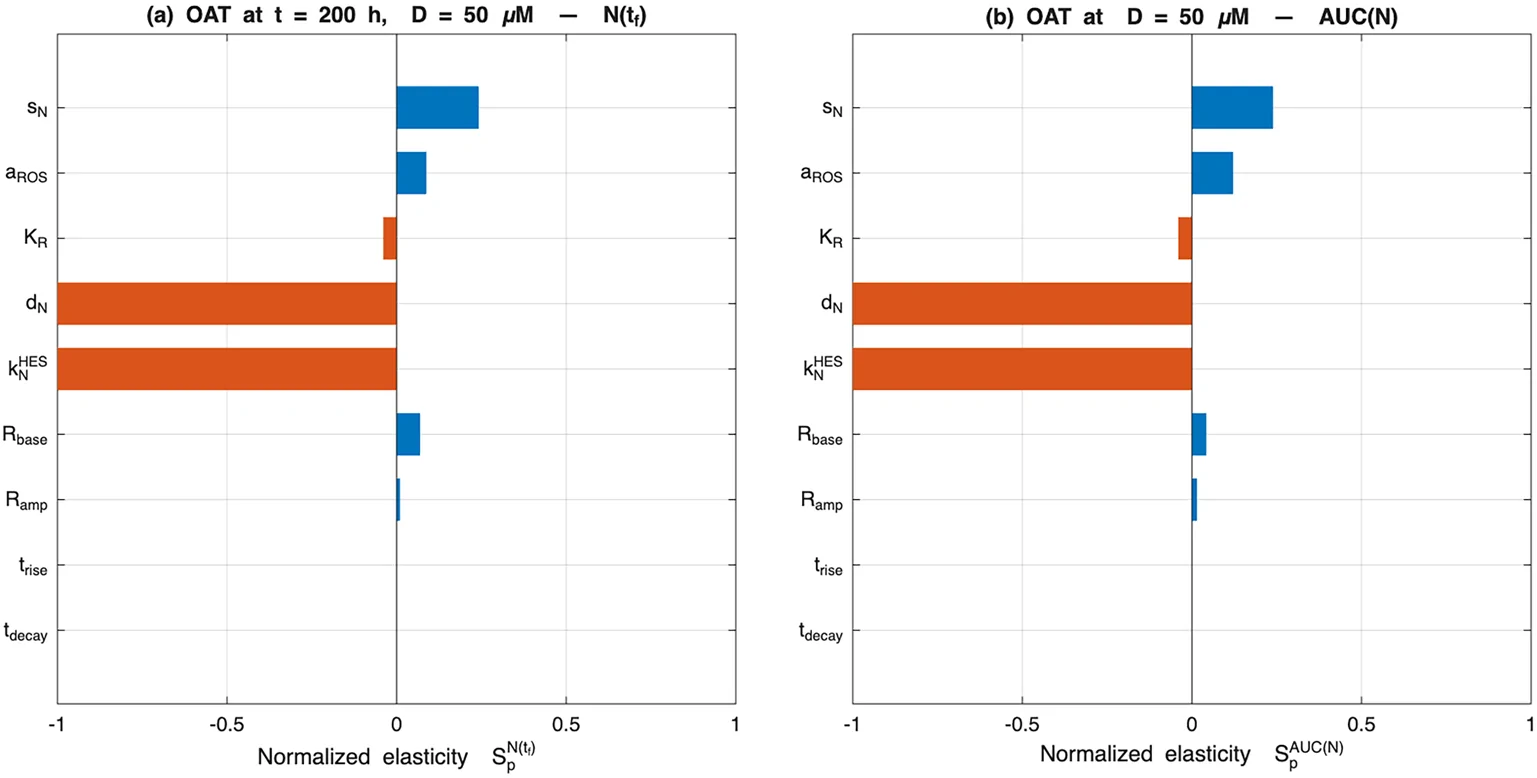
OAT sensitivity analysis (NRF2 redox regulation under hesperidin, D=50 µM). Normalized elasticities are shown for (a) the terminal NRF2 level N(tf) and (b) the area-under-the-curve AUC(N), evaluated at t=200 h. Blue bars denote parameters for which an increase produces a positive change in the output, while orange bars indicate parameters with a negative influence.

**Figure 40.**
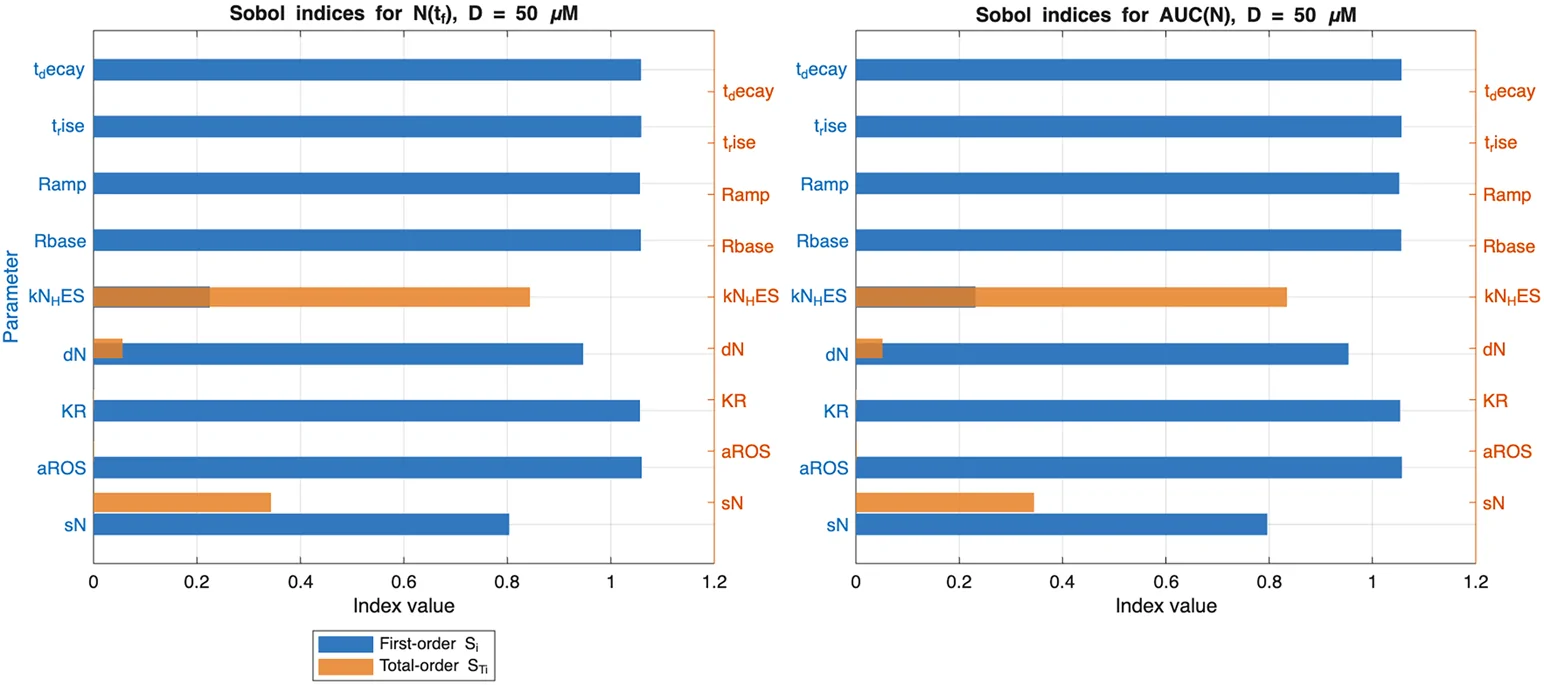
Sobol’ (Saltelli) global sensitivity analysis for NRF2 dynamics under hesperidin at D=50 µM. Panel (a) reports the first-order ($S_{1}$, blue) and total-order ($S_{t_{i}}$, orange) Sobol’ indices for $N(t_{x})$ at t=200 h, while panel (b) shows the corresponding indices for AUC(N) over 0–200 hours.

Overall, the metabolic and redox modules indicate that the most informative output is not only the predicted direction of hesperidin-mediated change, but also the identification of sensitive control points within the system. Glucose dynamics was governed mainly by supply, uptake, and clearance parameters, lactate dynamics by clearance and hesperidin-associated removal terms and NRF2 dynamics by deactivation and ROS-dependent activation parameters. These results suggest candidate biomarkers and regulatory nodes that may be prioritized in future experimental validation.

### 3.2. Sensitivity analysis

This section provides the results of the sensitivity analysis, highlighting the effect of parameter changes on model performance and stability.

Taken together, the OAT and Sobol’ analyses indicate that the dominant determinants of model behaviour are not uniformly distributed across all parameters. Instead, the most influential parameters consistently clustered around several biological processes, including tumour growth kinetics, immune-cell turnover, CAF-mediated stromal regulation, lactate metabolism, hypoxia signalling and hesperidin-associated modulation terms. Parameters controlling tumour proliferation and immune-cell dynamics strongly influenced tumour burden and immune-related output, whereas CAF-, ECM- and MMP-associated parameters emerged as major regulators of stromal remodelling. Similarly, lactate-related parameters exerted substantial effects across metabolic and immune modules, supporting the concept that metabolic adaptation may act as a central coordinating mechanism within the TME. Hypoxia- and angiogenesis-related output were primarily governed by HIF−1α regulatory parameters, highlighting oxygen-sensing pathways as important control points within the model. Collectively, these findings suggest that tumour growth, immune regulation, stromal remodelling, metabolic adaptation and hypoxia signalling represent the principal biological leverage points governing the simulated response to hesperidin. The integrated response analysis revealed coordinated dose-dependent changes across multiple biological subsystems (figure 41). The most pronounced responses were observed in immune regulation, where the effector-to-regulatory T-cell ratio (E/R) and M1/M2 macrophage ratio increased approximately threefold at 100 µM hesperidin. Simultaneously, strong suppression of hypoxia- and angiogenesis-related output was observed, with HIF-1α and vasculature indices decreasing to 5% and 10% of control values, respectively. Marked reductions were also detected in lactate accumulation and NRF2 activity, both declining to less than 10% of baseline levels. Stromal-remodelling markers exhibited more moderate responses, with CAF abundance, ECM stiffness and MMP activity decreasing to 59%, 32% and 81% of control values, respectively. By contrast, tumour burden was only modestly reduced (3% decrease), indicating that the dominant effects of hesperidin within the model were exerted through immune, angiogenic, metabolic and stromal regulatory pathways rather than through direct suppression of tumour growth.

**Figure 41.**
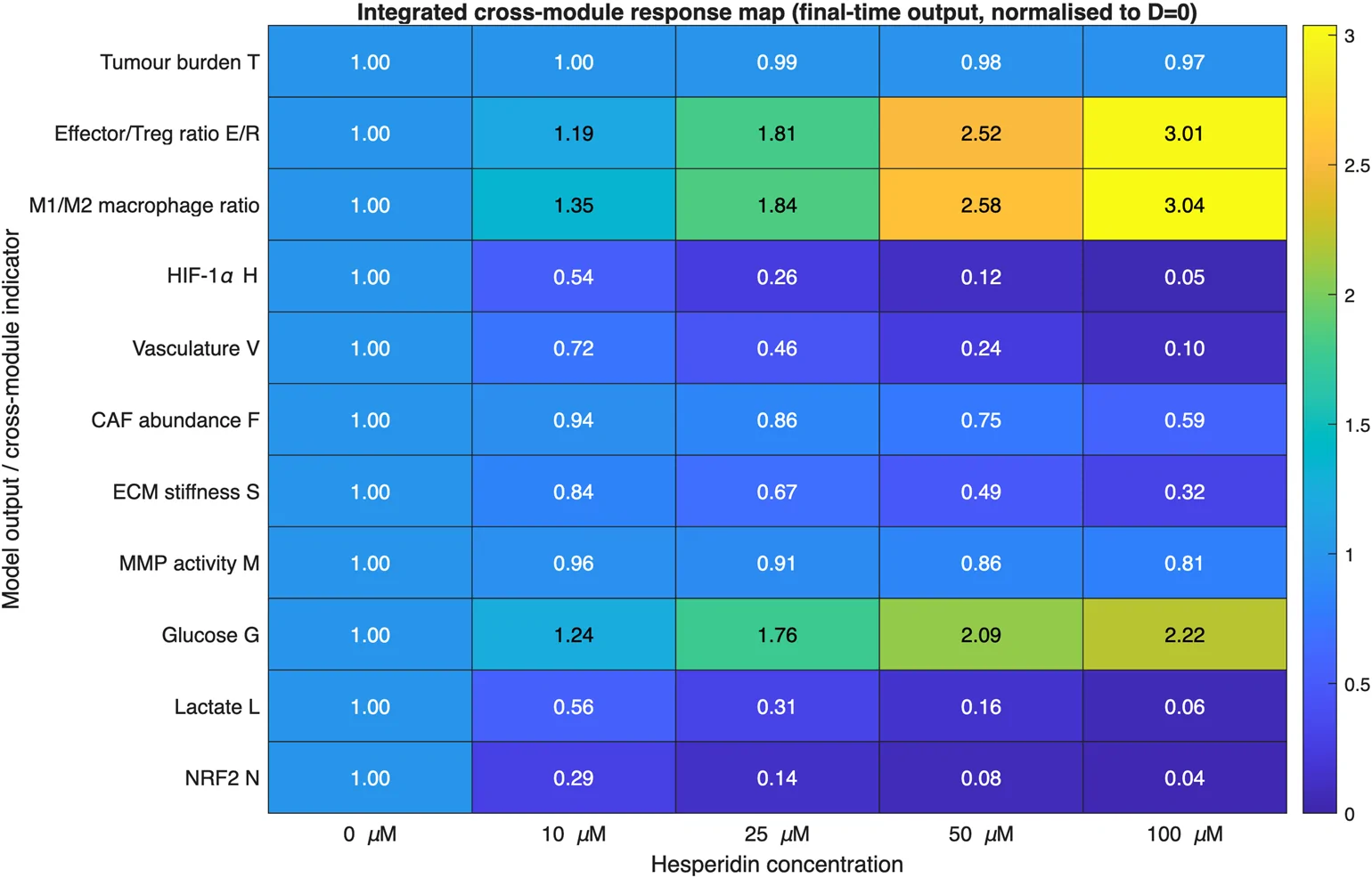
Integrated cross-module response map under hesperidin exposure. Normalized final-time output from tumour, immune, stromal, angiogenic, metabolic and redox modules across increasing hesperidin concentrations. The heatmap illustrates coordinated system-level responses arising from interactions among multiple biological subsystems within the mechanistic model. Values are expressed relative to the untreated condition (D=0 µM), with 1.0 corresponding to the control state. Blue colours indicate output below control levels (decrease), green colours indicate values moderately above control and yellow colours indicate the strongest increases relative to control.

## 4. Discussion

The logistic tumour-growth model (equation (2.1)) shows that intrinsic proliferation parameters ($r_{T}$, $K_{T}$) and immune-mediated killing ($k_{E}\;E$) govern PA-1 expansion. Within the model, incorporating hesperidin as a dose-dependent enhancer of cytotoxic T-cell activity resulted in reduced tumour burden. This aligns with reports that hesperidin modulates PI3K/Akt, MAPK/ERK and apoptosis/angiogenesis pathways, and that flavonoids enhance $\text{CD}8^{+}$ T-cell activity and regulate PD−1/PD−L1 and TGF−β signalling in the TME [68].

The simulated effector T-cell dynamics showed that hesperidin enhances tumour-driven effector proliferation via the Hill function $D_{\eta }(D)$, producing a 2–2.5-fold increase in E(t) at higher doses. This aligns with evidence that hesperidin restores $\text{CD}8^{+}$ T-cell activation under oxidative and immunosuppressive stress by reducing ROS and normalizing cytokine output such as IFN−γ and IL−2 [13,69]. Hesperidin also suppresses the PD−1/PD−L1 axis and TGF−β signalling, relieving Treg-mediated inhibition [70]. Within the proposed model, these effects translate into increased $E/E_{\text{control}}$, reflecting improved anti-tumour immune surveillance.

Within the model, the predicted reduction of Tregs indicates disruption of metabolic–cytokine support (lactate, TGF−β/IL−10). Tumour-resident Tregs depend on such adaptations for immune evasion [71], and flavonoids are recognized as adjuvants capable of limiting this suppression. The model shows dose-dependent separation of effector and suppressor axes, suggesting hesperidin-enabled windows for immune reactivation. This supports exploring combinations with checkpoint inhibitors or adoptive T-cell therapy, particularly in TME settings enriched in Tregs and lactate.

The model predicts that hesperidin increases $M_{1}$ macrophages in a dose-dependent manner by enhancing effector-driven activation and reducing lactate-mediated suppression. The model predicts a twofold rise at 50–100 µM, indicating repolarization from $M_{2}$ to $M_{1}$. This is consistent with evidence that hesperidin and related flavonoids modulate macrophage polarization through JAK2/STAT3 and PI3K/Akt pathways [13,72], supporting its possible future potential as a myeloid-modulating adjunct.

The model predicts a reduction in $M_{2}$ macrophages abundance under increasing hesperidin exposure via reduced lactate-/cytokine-driven polarization ($b_{L2}$, $g_{C2}$) and enhanced effector-mediated elimination and $M_{2}\;\to M_{1}$ repolarization ($a_{E2}$, $k_{pol}$). This mirrors its inhibition of NF−κB, a driver of IL-10/ARG1 expression and the $M_{2}$ phenotype [73,74]. As a result, angiogenesis, ECM remodelling and PD-L1 expression are reduced. The model suggests hesperidin shifts the TME towards an immune-active state and may synergize with immunotherapies, particularly when applied early [75].

In the presented model, the hesperidin dose-dependently suppresses hypoxia-induced HIF−1α, consistent with known mechanisms [76]. Under oxygen deficit, HIF−1α increases through the activation term $a_{H}\frac{O_{0}-O(t)}{O_{0}}H$; hesperidin counters this via $k_{H}^{\mathrm{HES}}DH$. Empirical studies similarly show reduced HIF−1α and VEGF [6,76]. Lower HIF−1α reduces angiogenesis, glycolysis and survival in hypoxic niches. Thus, hesperidin acts as a hypoxia-modulating agent that limits adaptation pathways supporting immune evasion and treatment resistance [77].

Vascular simulations show that hesperidin suppresses angiogenesis through direct inhibition and via reduced HIF−1α. Hypoxia-induced HIF−1α drives vasculature expansion ($a_{V}H$), while hesperidin counters this via $k_{V}^{\mathrm{HES}}DV$. Experimental work shows reduced vascular density and VEGF signalling in breast and lung cancer models [6,78]. Higher doses (50–100 µM) yielded sustained reductions in $V/V_{\mathrm{control}}$, suggesting that the model predicts attenuation of vascular support for tumour growth, which may contribute to microenvironment normalization [79,80].

Modelled CAF dynamics showed that hesperidin attenuates fibroblast activation through the suppression term $k_{F}^{\mathrm{HES}}DF$. Without treatment, tumour-derived signals ($a_{F}T$) drive CAF expansion, ECM deposition and stromal reinforcement [81]. Higher hesperidin doses reduce CAF growth, shifting the stroma towards a less pro-tumour state. This aligns with evidence that natural compounds can modulate fibroblast activation or senescence [13,82].

The model demonstrates that reduced CAF activity weakens ECM deposition terms ($\beta_{1}F$, $\beta_{2}FS$), disrupting the CAF–ECM stiffening loop. This matches evidence that CAF-driven collagen cross-linking increases stiffness, fostering malignancy, immune exclusion and resistance [48]. Matrix rigidity also affects macrophage polarization and immune-cell infiltration [83]. Thus, hesperidin may modulate not only cellular but also biomechanical TME features, improving mechanotransduction and drug penetration [84].

Within the model, suppressing CAFs lowers MMP activity ($\gamma_{F}F$), slowing ECM degradation and invasive potential. This is consistent with findings that hesperidin reduces MMP-2 and MMP-9 in tumour models [6,85]. Reduced MMP output produces a more stable matrix, aligning with literature on ECM breakdown and invasion [13,86]. While effective, the model collapses all MMP isoforms into one variable, limiting mechanistic resolution.

The model predicts reduced tumour and effector T-cell glucose use under increasing hesperidin exposure ($k_{u}$ and $k_{c}$), elevating extracellular glucose and improving energy availability for immune cells. This corresponds to evidence that hesperetin reduces GLUT1/GLUT4 and limits cancer glucose absorption [87]. Because nutrient competition strongly influences tumour–immune dynamics [88], the results suggest hesperidin combines direct tumour metabolic inhibition with microenvironmental normalization.

The model predicts reduced lactate accumulation under increasing hesperidin exposure by suppressing tumour glycolysis ($\alpha_{T}T\;G/(G+K_{L})$) and CAF-derived lactate ($\alpha_{F}F$), while enhancing clearance ($k_{L}^{\mathrm{HES}}D\;L$). This agrees with evidence that lactate drives acidosis, immune suppression and invasion [89]. Elevated lactate impairs T-cell and NK-cell function and promotes Tregs and myeloid-derived suppressor cells [90]. Thus, hesperidin may relieve metabolic stress and disrupt the acidified invasive niche.

NRF2 simulations show that higher hesperidin doses suppress ROS-driven NRF2 activation, tempering antioxidant signalling that supports tumour survival and chemoresistance. Flavonoids can activate NRF2 in healthy tissue but curb its overactivation in tumours [13,91,92]. Thus, hesperidin may act as a redox normalizer, restoring oxidative balance and potentially sensitizing tumour cells to ROS-based therapies.

The hesperidin concentrations investigated in the present framework (10-100 µM) were selected to explore the qualitative response of the model across a biologically relevant range commonly used in mechanistic *in vitro* studies. These concentrations should not be interpreted as clinically achievable tumour exposures because the framework does not include pharmacokinetic processes such as absorption, distribution, metabolism, excretion or tumour-specific drug accumulation. Consequently, the simulated dose-response relationships represent exploratory system-level responses to increasing biological modulation rather than predictions of therapeutic efficacy *in vivo*.

The integrated response analysis further highlights the interconnected nature of the modelled TME. Although individual subsystems were analysed separately, the strongest responses emerged across multiple coupled modules simultaneously. In particular, reductions in lactate were accompanied by increases in the E/R and $M_{1}/M_{2}$ ratios, together with suppression of HIF-1α, angiogenesis and stromal remodelling. These coordinated changes are biologically plausible, as lactate is increasingly recognized as a central signalling molecule linking tumour metabolism with immune suppression, macrophage polarization, hypoxia signalling and tumour progression within the TME. Consequently, perturbations affecting metabolic pathways may propagate across several biological compartments and generate system-level responses that are not apparent when individual pathways are considered in isolation [89,90,93,94].

Importantly, several trends reported above, including reduced tumour burden, suppression of immunosuppressive populations, attenuation of hypoxia signalling, reduced angiogenesis and modulation of metabolic and redox pathways, arise from biological assumptions explicitly incorporated into the governing equations. Therefore, these responses should not be interpreted as independent experimental discoveries. Rather, the principal contribution of the framework lies in revealing how tumour, immune, stromal, metabolic and redox subsystems interact and in identifying the processes that exert the greatest influence on overall system behaviour. Sensitivity analysis is widely used in systems biology to identify influential mechanisms, regulatory bottlenecks and candidate targets for experimental investigation rather than solely to evaluate model output [95]. Across the analysed modules, sensitivity analyses consistently identified tumour growth kinetics, immune-cell turnover, CAF-mediated stromal remodelling, lactate regulation and hesperidin-associated modulation parameters as dominant determinants of the predicted response. These findings emerged from the integrated behaviour of the coupled tumour–immune–stromal network and therefore provide systems-level insight beyond the assumptions embedded within any single equation. Consequently, the framework should be viewed primarily as a tool for mechanistic exploration, hypothesis generation and identification of candidate pathways and biomarkers for future experimental validation in ovarian cancer models.

Notably, several of the most influential parameters identified by the sensitivity analyses correspond to processes that also dominated the integrated response analysis, including lactate regulation, CAF-associated stromal remodelling, hypoxia signalling and immune-cell dynamics. This convergence suggests that these pathways act as major regulatory hubs within the model and may represent biologically relevant intervention points for future experimental investigation.

The framework therefore generates several experimentally testable hypotheses, including the prediction that lactate modulation may simultaneously alter Treg abundance and macrophage polarization, that suppression of CAF activity may reduce both ECM stiffness and MMP activity, and that attenuation of HIF−1α signalling may produce coordinated effects on angiogenesis and metabolic adaptation.

Although more complex nonlinear formulations may be appropriate for certain biological interactions, the present framework intentionally favoured parsimonious functional forms to maintain model interpretability and avoid introducing additional poorly constrained parameters. Future studies may evaluate alternative saturation kinetics as additional experimental data become available.

### 4.1. Limitations

The study is not without its limitations, which are presented below.

The proposed framework should not be interpreted as a direct representation of a PA-1 monoculture system. Rather, it represents a simplified *in silico* ovarian tumour ecosystem centred on PA-1 tumour cells and incorporating additional immune, stromal, angiogenic, metabolic and ECM compartments. Consequently, the simulated non-tumour populations reflect conceptual microenvironmental processes rather than cellular components directly present in standard PA-1 culture conditions.

Another important limitation is the absence of pharmacokinetic and pharmacodynamic modelling. Hesperidin exposure was represented as an external model input, and the investigated concentration range (10–100 µM) was selected for exploratory mechanistic purposes rather than derived from measured tumour concentrations. Consequently, the framework cannot determine whether these exposure levels are achievable *in vivo*, nor can it predict optimal dosing regimens, bioavailability, tissue distribution, metabolic conversion or systemic clearance. Integration of PK/PD models and experimentally measured tumour concentrations will therefore be necessary before any quantitative therapeutic conclusions can be drawn.

The logistic-growth model provides a simplified tumour-proliferation framework but omits spatial heterogeneity, clonal diversity and context-specific behaviour that shape treatment response. Similarly, the effector T-cell variable collapses multiple functional subsets and excludes exhaustion, trafficking delays and infiltration barriers. Treg modelling uses generic cytokine and lactate parameters rather than ovarian-cancer-specific data, and compresses complex TGF−β/IL−10 and lactate signalling into simplified terms ($a_{R}C$, $b_{R}L$), assuming uniform mixing despite spatially variable Treg niches. Macrophage modelling similarly reduces TAM heterogeneity to binary $M_{1}/M_{2}$ states [96], assumes instantaneous dose-dependent modulation, and does not include relevant signalling networks (e.g. GAS6/AXL/MerTK) or stromal factors—ECM stiffness, hypoxia gradients and vascular structure—that critically influence $M_{2}$ persistence [97–100]. The hypoxia module captures hesperidin-induced downregulation of HIF−1α but treats oxygen tension and redox balance as lumped variables, omitting diffusion, mitochondrial activity and prolyl-hydroxylase-domain-dependent degradation [77]. Crosstalk with NF−κB and NRF2 is only phenomenological, and kinetic constants are derived from heterogeneous literature rather than PA-1–specific oxygen-gradient data [79,101]. Vasculature is represented as a single scalar, ignoring spatial heterogeneity, vessel maturation and alternative angiogenesis modes such as co-option [102,103]. CAFs are treated as a homogeneous population [104], and ECM stiffness is reduced to a single scalar, omitting anisotropy, fibre alignment and spatially varying mechanics known to drive mechanotransduction and therapy resistance [48]. The MMP module collapses multiple isoforms (MMP-2, MMP-9 and MT1-MMP) into a single variable and does not represent spatial heterogeneity or TIMP-mediated regulation essential for modelling invasion fronts [52,105,106]. Similarly, glucose is modelled as a well-mixed nutrient pool, overlooking steep metabolic gradients, transporter heterogeneity and adaptive fuel switching [107,108]. Hesperidin’s metabolic effects are simplified to Hill-type reductions in uptake without PK/PD or bioavailability considerations. Lactate is also modelled as a uniform scalar, omitting diffusion, shuttling (MCT1/MCT4), lactylation and cell-type–specific production [109]. Finally, the NRF2 module treats downstream targets (HO-1 and NQO1) and KEAP1 feedback as absent and does not incorporate tumour-specific NRF2 isoforms or *in vivo* pharmacokinetics, which may modify hesperidin’s redox-modulatory efficacy [110]. An important limitation is that the model was not calibrated or validated against longitudinal PA-1 experimental measurements. Although the selected parameters were constrained by published biological evidence and sensitivity analyses demonstrated stable model behaviour, quantitative predictions should be interpreted cautiously. Future studies should integrate transcriptomic, proteomic, metabolic and phenotypic data from PA-1 cells or patient-derived ovarian cancer models to enable parameter estimation, calibration and external validation. Formal structural and practical parameter identifiability analyses were not performed. Consequently, multiple parameter combinations may potentially generate similar system behaviour, and future calibration studies will be required to determine uniquely identifiable parameter sets.

Although extensive efforts were made to associate parameter values with published biological evidence, not all processes represented in the model have experimentally established kinetic constants. Consequently, some parameters were derived from biologically plausible ranges reported in related modelling studies and evaluated through sensitivity analyses. Future experimental calibration will further improve quantitative parameter estimation.

Furthermore, several hesperidin-dependent mechanisms were incorporated into the governing equations based on published experimental evidence. Consequently, some predicted responses reflect the biological assumptions embedded within the model structure and should be interpreted as mechanistic hypotheses rather than experimentally validated quantitative outcomes. In addition, several state variables were represented as relative biological indices and proxy measures rather than directly measurable physical quantities, which limits direct quantitative comparison between simulated output and experimental observations. Consequently, the primary value of the present framework lies in mechanistic exploration, hypothesis generation and identification of influential biological pathways rather than quantitative prediction of treatment efficacy.

The relatively large number of state variables and parameters represents an additional limitation of the present framework. Although the model was intentionally designed to capture multiple interconnected biological processes reported to be influenced by hesperidin, the resulting dimensionality increases parameter uncertainty and may introduce identifiability challenges. Consequently, multiple parameter combinations could potentially reproduce similar qualitative behaviours. While local and global sensitivity analyses were used to identify the dominant determinants of system behaviour, future studies should investigate formal parameter identifiability, model reduction strategies and calibration against experimental datasets to determine the minimum level of complexity required to reproduce the principal biological mechanisms represented here. Although the framework incorporates multiple biological subsystems, simplifications were applied wherever possible; for example, immune-cell populations were aggregated into representative compartments, CAF heterogeneity was omitted, multiple MMP isoforms were combined into a single variable and angiogenesis, metabolism, redox regulation and ECM remodelling were represented using lumped state variables to preserve interpretability and computational tractability.

An additional limitation arises from the representation of biological processes operating on different characteristic time scales within a common 200-hour simulation window. Immune-cell activation and metabolic responses may occur within hours to days, whereas ECM remodelling, stromal adaptation and angiogenic restructuring frequently evolve over longer periods. Consequently, the model should be interpreted primarily as a framework for analysing relative interactions among biological subsystems rather than as a source of precise temporal predictions. Future studies incorporating experimental calibration and extended simulation horizons may further investigate the influence of time-scale separation on system behaviour.

Formal model validation against independent experimental or clinical datasets was not performed; therefore, the present framework should be regarded as a tool for mechanistic exploration and hypothesis generation rather than quantitative prediction. The framework is not patient-specific and does not incorporate patient-derived genomic, transcriptomic, proteomic, imaging or clinical data. Consequently, it cannot capture inter-patient heterogeneity or support personalized prediction of tumour progression, treatment response or clinical outcome.

## 5. Conclusion

The ODE-based mechanistic framework shows that hesperidin can potentially modulate tumour–stroma–immune interactions and help restore TME equilibrium, indicating the potential to enhance therapy effectiveness in solid tumours. These findings should be interpreted as qualitative mechanistic hypotheses rather than quantitatively validated predictions, highlighting candidate pathways and interactions for future experimental investigation in ovarian cancer models. This study highlights how natural compounds may influence key TME pathways and supports their exploration in preclinical and potentially in future translational settings. Despite current modelling constraints, the proposed approach offers a useful framework for identifying ovarian cancer therapeutic targets and predicting system-level effects of TME-modifying agents.

## Figures and Tables

**Table 5.** Biological rationale and evidence supporting hesperidin-modulated mechanisms incorporated into the model.

model term	biological interpretation	supporting evidence	evidence level
$k_{T}^{\mathrm{HES}}$	reduction of tumour viability and induction of apoptosis	hesperidin reduces ovarian cancer cell viability and induces apoptosis through ER-stress signalling pathways; enhanced cytotoxicity has also been reported in ovarian cancer cells [111,112].	ovarian cancer
$k_{E}^{\mathrm{HES}}$	enhanced effector T-cell anti-tumour activity	immunomodulatory activity, enhancement of anti-tumour immune responses and regulation of inflammatory cytokines have been reported in multiple experimental models [113].	cancer-general
$k_{R}^{\mathrm{HES}}$	reduction of Treg-mediated immunosuppression	hesperidin has been reported to regulate inflammatory and immunosuppressive signalling pathways [113].	cancer-general
$k_{\mathrm{pol}}$	promotion of M2→M1 macrophage repolarization	macrophage-modulating and anti-inflammatory activity has been reported in cancer and inflammatory disease models [113].	cancer-general
$k_{F}^{\mathrm{HES}}$	suppression of CAF activation and stromal remodelling	evidence supports hesperidin-mediated inhibition of fibroblast activation and tumour-supportive stromal signalling [53].	fibroblast/tumour-stroma evidence
$k_{H}^{\mathrm{HES}}$	inhibition of HIF−1α signalling	HIF−1α is a key driver of ovarian cancer progression; hesperidin and related flavonoids suppress hypoxia-associated pathways [54,55].	ovarian cancer pathway support
$k_{V}^{\mathrm{HES}}$	reduction of VEGF-dependent angiogenesis	anti-angiogenic activity of hesperidin and suppression of VEGF signalling have been reported in ovarian and other cancer models [14,54,55].	ovarian cancer pathway support
$k_{L}^{\mathrm{HES}}$	rduction of lactate accumulation and metabolic stress	suppression of glycolytic metabolism and lactate production has been reported in tumour models [56].	other cancer type; colorectal/metabolic evidence
$k_{N}^{\mathrm{HES}}$	modulation of NRF2 activity and oxidative-stress responses	hesperidin regulates NRF2/HO−1 signalling and antioxidant responses [61,114].	cancer-general
$k_{O}^{\mathrm{HES}}$	indirect reduction of ROS through NRF2-mediated antioxidant activity	NRF2 -dependent antioxidant pathways are established regulators of oxidative stress in ovarian cancer and other tumour systems [57,62].	ovarian cancer + cancer-general

**Table 6.** Summary of model state variables and biological interpretation.

variable	description	units	biological interpretation
T	tumour-cell abundance	scaled cells	ovarian tumour cell population (PA-1 reference context)
E	effector T-cell abundance	scaled cells	cytotoxic anti-tumour immune response
R	regulatory T-cell abundance	scaled cells	immunosuppressive T-cell compartment (Tregs)
$M_{1}$	M1 macrophage abundance	scaled cells	pro-inflammatory, anti-tumour macrophages
$M_{2}$	M2 macrophage abundance	scaled cells	immunosuppressive, pro-tumour macrophages
F	CAF abundance	scaled cells	activated stromal fibroblast population
H	HIF-1α activity	arb. units	relative hypoxia-signalling activity
V	vasculature index	arb. units	relative tumour vascularization and angiogenic capacity
S	ECM stiffness index	arb. units	relative biomechanical stiffness of the TME
M	MMP activity	arb. units	aggregate ECM-degrading proteolytic activity
G	glucose availability index	arb. units	relative glucose concentration within the TME
L	lactate accumulation index	arb. units	relative glycolytic lactate burden
N	NRF2 activity	arb. units	relative antioxidant and redox-regulatory signalling activity
O	ROS activity	arb. units	relative oxidative-stress signal driving NRF2 activation
C	suppressive cytokine signal	arb. units	aggregate immunosuppressive cytokine activity (e.g. TGF-β and IL-10)
P	immune checkpoint signal	arb. units	aggregate inhibitory immune-checkpoint activity (e.g. PD-1/PD-L1 axis)
D	hesperidin concentration	µM	external model input representing constant hesperidin exposure

*Note:* Cellular variables (T, E, R, $M_{1}$, $M_{2}$ and F) represent effective scaled abundances rather than exact cell counts. Signalling, angiogenic, metabolic, stromal and redox variables are expressed in relative arbitrary units (arb. units) and should be interpreted as semi-quantitative state indices. Variables O, C and P are auxiliary biological signals used to couple model subsystems, whereas D represents the externally imposed hesperidin exposure.

## Data Availability

The MATLAB code supporting this work has been archived and is publicly accessible on Zenodo (http://doi.org/10.5281/zenodo.17664294).
